# Quinazoline-4(3*H*)-one-7-carboxamide Derivatives as Human Soluble Epoxide Hydrolase Inhibitors with Developable 5-Lipoxygenase Activating Protein Inhibition

**DOI:** 10.1021/acsomega.2c04039

**Published:** 2022-10-05

**Authors:** Sümeyye Turanlı, Azize Gizem Ergül, Paul M. Jordan, Abdurrahman Olğaç, Burcu Çalışkan, Oliver Werz, Erden Banoglu

**Affiliations:** † Department of Pharmaceutical Chemistry, Faculty of Pharmacy, Gazi University, Taç Sok. No: 3 Yenimahalle, 06560 Ankara, Turkey; ‡ Department of Pharmaceutical/Medicinal Chemistry, Institute of Pharmacy, Friedrich Schiller University Jena, Philosophenweg 14, D-7743 Jena, Germany

## Abstract

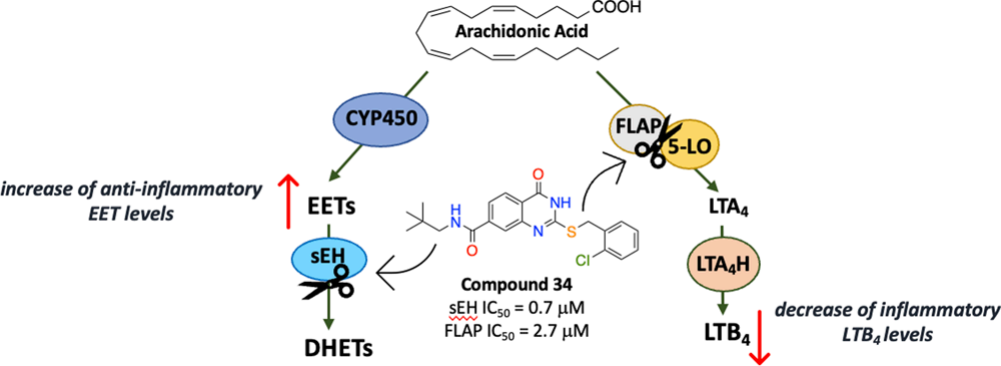

Soluble epoxide hydrolase (sEH) metabolizes epoxyeicosatrienoic acids (EETs), which are endowed with beneficial biological activities as they reduce inflammation, regulate endothelial tone, improve mitochondrial function, and decrease oxidative stress. Therefore, inhibition of sEH for maintaining high EET levels is implicated as a new therapeutic modality with broad clinical applications for metabolic, renal, and cardiovascular disorders. In our search for new sEH inhibitors, we designed and synthesized novel amide analogues of the quinazolinone-7-carboxylic acid derivative **5**, a previously discovered 5-lipoxygenase-activating protein (FLAP) inhibitor, to evaluate their potential for inhibiting sEH. As a result, we identified new quinazolinone-7-carboxamides that demonstrated selective sEH inhibition with decreased FLAP inhibitor properties. The tractable SAR results indicated that the amide and thiobenzyl fragments flanking the quinazolinone nucleus are critical features governing the potent sEH inhibition, and compounds **34**, **35**, **37**, and **43** inhibited the sEH activity with IC_50_ values of 0.30–0.66 μM. Compound **34** also inhibited the FLAP-mediated leukotriene biosynthesis (IC_50_ = 2.91 μM). In conclusion, quinazolinone-7-carboxamides can be regarded as novel lead structures, and newer analogues with improved efficiency against sEH along with or without FLAP inhibition can be generated.

## 1 Introduction

**Figure 1:**
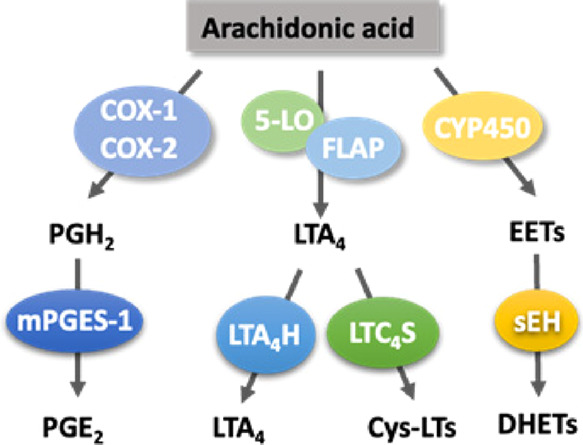
COX, LO, and CYP450-mediated AA metabolism and the potential therapeutic targets.

Arachidonic acid (AA) metabolism is conferred by three main enzymatic pathways producing various bioactive lipid mediators that regulate a wide range of inflammatory processes and also maintain homeostasis (Figure 1).^1^ The most studied cyclooxygenase (COX) and 5-lipoxygenase (5-LO) branches, which are associated with the generation of inflammatory mediators such as prostaglandin (PG)E_2_ and leukotriene (LT)B_4_, have been in the longstanding focus of big pharma for the development of best-selling nonsteroidal anti-inflammatory drugs (NSAIDs) and anti-asthmatics, respectively.^2,3^ However, the cytochrome (CYP)P450 branch has later attracted the attention of researchers because of its potential as a novel molecular target.^4^

The CYP P450 branch of the AA metabolism generates both the epoxyeicosatrienoic acids (EETs) and their corresponding (di)hydroxy metabolites, that is, (di)hydroxyeicosatrienoic acids (DHETs) and 20-hydroxyeicosatetraenoic acid (20-HETE), which have opposing biological actions, that is, vasodilator and anti-inflammatory vs vasoconstrictor and pro-inflammatory, respectively.^5,6^ Therefore, the imbalance between these fatty acids and changes in their levels have been related to numerous pathological conditions such as hypertension, inflammation, and neurodegeneration.^5^ EETs are critical regulators of homeostasis, relative to other metabolites in the same pathway (Figure 1), because of their complex biology in which they suppress inflammation, regulate vascular tone, lower blood pressure, and stabilize mitochondria, reducing the formation of reactive oxygen species (ROS) and endoplasmic reticulum (ER) stress.^7^ For this reason, new therapeutic approaches target the CYP450 branch as a means to increase the amount of these beneficial EETs to generate effective therapeutic modalities for associated disorders. Because the degradation of beneficial EETs to detrimental DHETs is carried out by the action of soluble epoxide hydrolase (sEH, EC 3.3.2.10), the discovery of sEH inhibitors became an intriguing proof-of-concept for this emerging target space with the aim of enhancing and prolonging the biological functions of endogenous EETs.^8−10^

**Figure 2:**
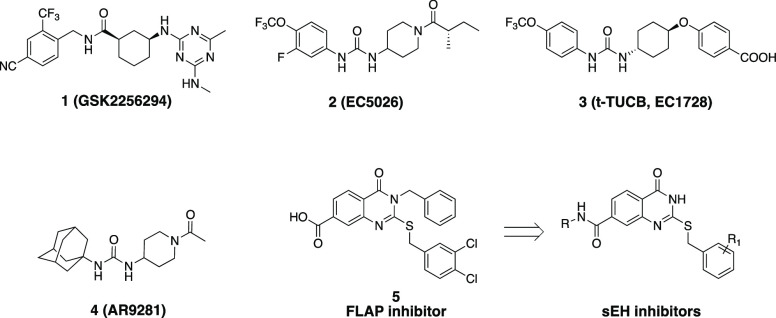
Examples of advanced sEH inhibitors (**1**–**4**) and evolution of potent sEH inhibitor quinazolinone-amides (**6**) from the FLAP inhibitor **5**.

sEH possesses both C-terminal hydrolase and N-terminal phosphatase activities where the hydrolase domain carries out the hydrolysis of beneficial EETs to rather harmful DHETs (Figure 1).^11,12^ Amino acid sequences of sEH including the catalytic Asp335, Tyr383, and Tyr466 residues are highly conserved among different species such as humans, mice, rats, and pigs (Figure S1). Although the biological role of the N-terminal phosphatase action is still under debate,^13,14^ various drug companies and many research groups aim at targeting the sEH hydrolase domain toward anti-inflammatory and anti-hypertensive drug development, as exemplified with compounds **1**–**4**, as shown in Figure 2.^15−18^ However, no sEH inhibitor has yet reached clinical use, as reviewed elsewhere.^19,20^ Nevertheless, numerous preclinical studies along with a few clinical trials revealed the far-reaching therapeutic potential of sEH inhibitors against a variety of disorders such as metabolic, renal, cardiovascular, and neurodegenerative diseases.^8^

In the light of the crystallographic fragment screening and the available structure–activity relationship (SAR) data, a number of critical molecular features were elucidated for the development of effective sEH inhibitors.^21−24^ For instance, the central urea or amide functions were shown to be primary pharmacophores for optimum inhibitor–protein interactions along with the mainly hydrophobic and bulky fragments flanking the primary pharmacophore, which apparently stabilizes the inhibitor at the active site of sEH by space-filling properties.^23^ However, these structural features often cause most of the reported sEH inhibitors to have poor physicochemical features with unfavorable biocompatibility properties, precluding their further development as clinical candidates. Therefore, there is a clear need to define new scaffolds to expand the chemical space and enable continued development of improved sEH inhibitors.

As part of our research efforts to develop novel anti-inflammatory drug candidates aiming at distinct targets within the AA cascade, that is, 5-LO-activating protein (FLAP) and microsomal prostaglandin E_2_ synthase-1 (mPGES-1),^25−29^ we previously identified a quinazolinone-7-carboxylic acid derivative (**5**) as a novel FLAP inhibitor^30^ without any activity against sEH. The available SAR data with sEH persuaded us to develop amide derivatives of compound **5**, aiming to find new candidates with improved affinity toward sEH. Thus, we conducted the diversification of the compound **5** scaffold, and we report here our success in identifying new quinazolinone-7-carboxamides that potently inhibit sEH with or without FLAP inhibitor activity. We propose that this new chemotype is prone to further development of selective sEH or dual sEH/FLAP inhibitors as potential anti-inflammatory agents.

## 2 Results and Discussion

### 2.1 Chemistry

**Scheme 1:**
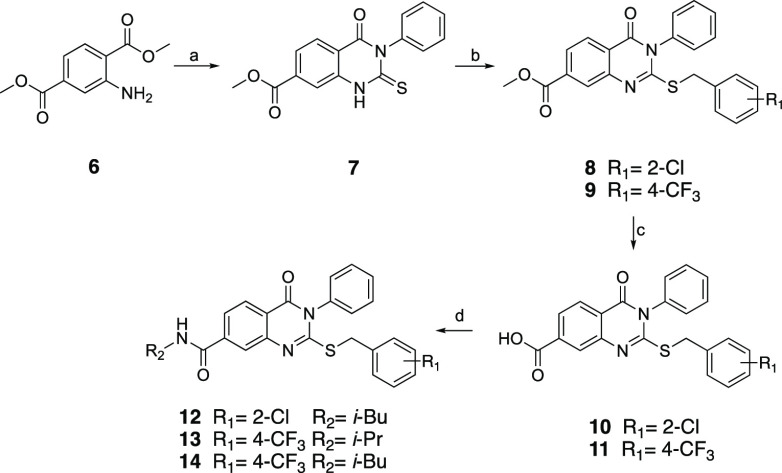
Reaction Conditions and Reagents: (a) Phenyl Isothiocyanate, Pyridine, 8 h, 100 °C; (b) Alkyl Halide Derivative, Cs_2_CO_3_, DMF, 3 h, rt; (c) 20% NaOH Solution, Isopropanol, 2 h, Reflux; (d) CDI, Amine Derivative, Anhydrous Dioxane, Reflux

The reaction of dimethyl aminoterephthalate **6** with the phenyl isothiocyanate in refluxing pyridine generated the 3-phenyl-2-thioxoquinazoline-4-one (**7**) intermediate,^31^ which was reacted with the appropriate benzyl halide to produce the corresponding benzylated derivatives **8** and **9**. Subsequently, hydrolysis of the ester group yielded the carboxy intermediates **10, 11**, which was subsequently coupled with the appropriate amines to afford the expected amide derivatives **12**–**14** (Scheme 1).

**Scheme 2:**
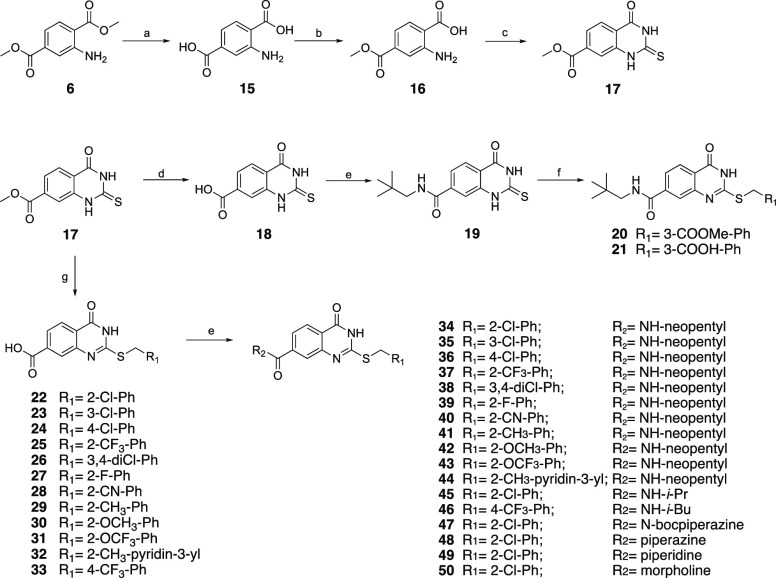
Reaction Conditions and Reagents: (a) LiOH•H_2_O, THF:H_2_O, 2 h, Reflux; (b) Trimethylchlorosilane, MeOH, 4 h, Reflux; (c) (i) SOCl_2_, 2 h, Reflux; (ii) NH_4_SCN, Acetone, 3 h, rt; (d) 1 N NaOH Solution, IPA, 2 h, Reflux; (e) CDI, Amine Derivative, Anhydrous Dioxane, Reflux; (f) Alkyl Halide Derivative, K_2_CO_3_, Acetone, 3 h, Reflux; (g) Alkyl Halide Derivative, 1 N NaOH Solution, Ethanol, 3 h, Reflux

The synthetic route to the desired 2-benzylthio-dihydrohydroquinazoline-7-carboxamides (**20**, **21**, **34–50**) is outlined in Scheme 2. Dimethyl aminoterephthalate **6** was first hydrolyzed to 2-aminoterephthalic acid **15**. The selective esterification of carboxylic acid located at 4 position by use of trimethylchlorosilane in methanol generated the monoester **16**.^32^ Compound **16** was first treated with thionyl chloride to produce acid chloride, which was reacted with ammonium isothiocyanate to form the common quinazoline intermediate **17** after spontaneous ring closure.^33^ Treatment of **17** with sodium hydroxide followed by neopentylamine provided the amide derivative **19**, which was then alkylated to afford desired compounds **20** and **21**. For the synthesis of target compounds **34**–**50**, intermediate **17** was simultaneously alkylated and hydrolyzed with appropriate alkyl halides in the presence of sodium hydroxide to yield 7-carboxyquinazolinones (**22**–**33**), which were then conveniently used to synthesize corresponding 7-carboxamide analogues (**34**–**50**) by reaction with appropriate amines (Scheme 2).

**Scheme 3:**
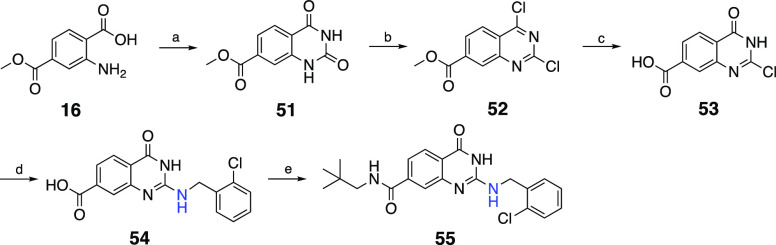
Reaction Conditions and Reagents: (a) Urea, Acetic Acid, 5 h, Reflux; (b) POCl_3_, DIEA, Toluene, 6 h, 90 °C; (c) 1 N NaOH Solution, 2 h, rt; (d) 2-Chlorobenzylamine, DIEA, Ethanol, 3 h, Reflux; (e) CDI, Neopentylamine, Anhydrous Dioxane, Reflux

The 2-aminobenzyl congener (**55**) of compound **34** was obtained starting from **16**, as outlined in Scheme 3. Hence, intermediate **16** was reacted with urea to generate quinazolinedione **51**, which was subsequently treated with phosphorus oxychloride in the presence of *N*,*N*-diisopropylethylamine to obtain 2,4-dichloroquinazoline **52**.^34^ Then, **52** was first treated with sodium hydroxide to yield 2-chloro-4-oxo-quinazoline **53**, which was then sequentially converted to 2-aminobenzyl (**54**) and carboxamide analogues (**55**) in good yield.

### 2.2 Biological Evaluation and SAR

We previously identified compound **5**, having a 4-oxo-quinazoline-7-carboxylic acid scaffold, as a new chemotype for inhibition of cellular leukotriene (LT) biosynthesis by targeting FLAP (IC_50_ = 0.87 μM).^30^ However, compound **5** lacks the typical urea or amide pharmacophores of common sEH inhibitors, which are essential to establish hydrogen bonds with key residues at the central region of the sEH active site such as Tyr383, Tyr466, and Asp355.^4^ In addition, there are strong implications that multitarget inhibitors within the AA cascade might demonstrate a better risk–benefit profile compared to single-target inhibitors,^35^ and a combination of sEH inhibition and the inhibition of LT biosynthesis has been shown to be beneficial in several occasions.^36−39^ Therefore, we took advantage of the presence of the free carboxyl group in **5** and installed an amide backbone with the aim of introducing a sEH inhibitory potency in the newly designed analogues (Table 1). Accordingly, we implemented different amide pendants to the quinazoline ring, combined with variations on the S-benzyl and N^3^-benzyl moieties of the quinazolinone ring in **5**.

**Table 1:**
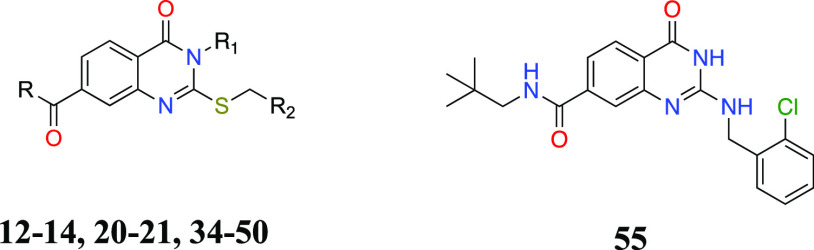

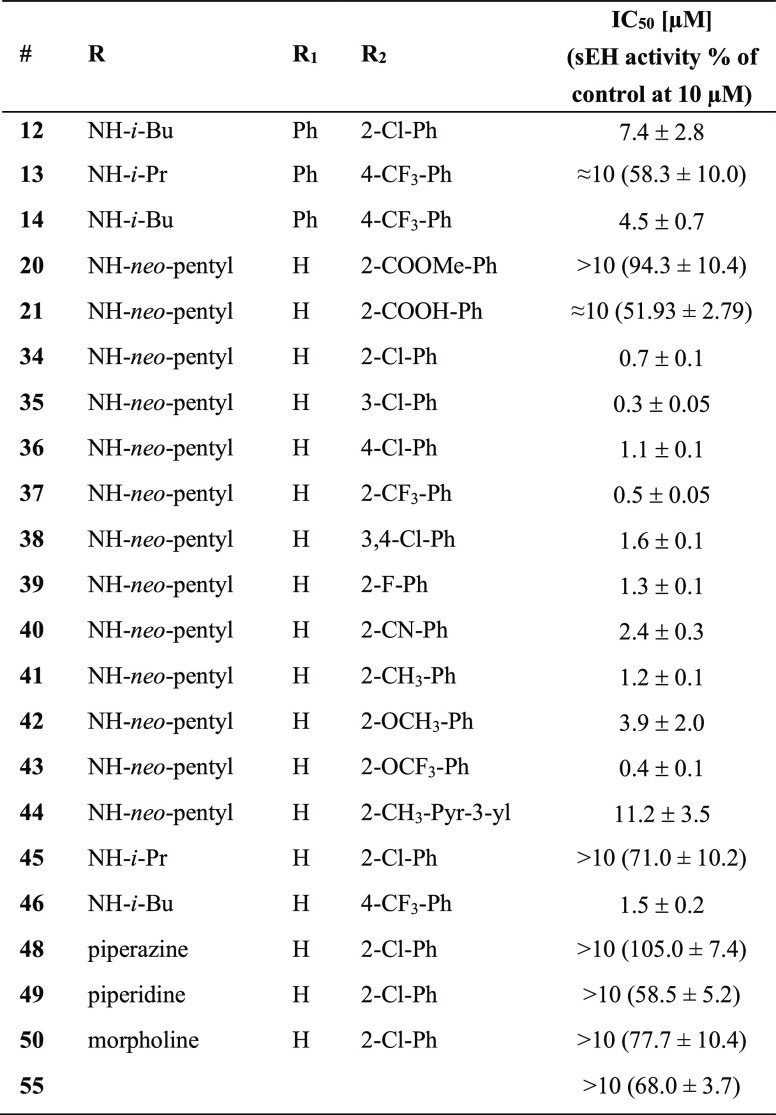
In Vitro Inhibitory Activities of Newly Identified sEH Inhibitors in a Cell-Free Assay^a^

a Data (means ± SEM, *n* = 3 determinations) are given as residual activity in the percentage of control (100%, vehicle, uninhibited control) or IC_50_ values.

As we initially envisaged, amidation of the free carboxyl group in **5** generated new analogues with inhibitory activity against human sEH with IC_50_ values in the range of 0.3 to 11.2 μM (Table 1); however, the inhibition potential was strongly dependent on the nature of amide groups as well as the modifications on the benzyl functions. For example, comparison of compound **14** (IC_50_ = 4.5 μM) and **46** (IC_50_ = 1.5 μM) indicates that a free NH at 3-position of the quinazoline moiety is preferred for the sEH inhibition because compound **14** with a N^3^-phenyl substituent was threefold less potent. The subsequent finding was the implementation of larger aliphatic groups at the amide part because we observed a tendency for sEH in which the neopentyl is preferred over isobutyl or isopropyl at the amide part, indicating that the larger hydrophobic moiety improved the inhibitory potency (**34** with neopentyl, IC_50_ = 0.7 μM vs **45** with *i*-pr, IC_50_ > 10 μM). Tertiary amide counterparts of **34** prepared with cyclic amines such as piperazine (**48**), piperidine (**49**), and morpholine (**50**) were also inactive against sEH (IC_50_ > 10 μM), suggesting that the presence of an amide NH with H-bond donor properties is important for the observed sEH inhibitory potency.

From this point on, we kept the neopentyl substituent at the amide part for later SAR studies because of its good impact on potency and modified the thiobenzyl moiety to explore the substituent effect on the inhibitory activity. The relocation of 2-chlorine in **34** at the 3-position was beneficial (**35**, IC_50_ = 0.3 μM) while chlorine at the 4-position caused a decrease in the inhibitory potency (**36**, IC_50_ = 1.1 μM). When the di-substitution pattern was explored, the 3,4-dichloro analogue (**38**) was also found to be less potent than **35** with IC_50_ = 1.6 μM, altogether suggesting that chlorine is preferred at the *ortho*-or *meta*-position of the phenyl ring for effective sEH inhibition.

We next examined the necessity of the 2-chlorine by replacing it with a variety of substituents. By replacing with a large and polar carboxyl group (**21**) or its ester (**20**), a loss of activity was apparent (IC_50_ > 10 μM). However, incorporation of smaller fluorine (**39**), linear cyano (**40**), or larger methyl (**41**) and methoxy (**42**) groups instead of 2-chlorine moderately restored the inhibitory activity in this series with IC_50_ in the range of 1.2 to 3.9 μM, indicating steric as well as electronic substituent effects of the 2-substituent that influence the inhibitory activity. Finally, replacement of 2-chlorine by bulkier-hydrophobic trifluoromethyl (**37**, IC_50_ = 0.5 μM) or trifluoromethoxy (**43**, IC_50_ = 0.4 μM) groups was found to be more suitable, suggesting the significance of the 2-substituted S-benzyl group for inhibition of sEH activity. In addition, we briefly examined the introduction of a heteroaromatic group at this location, such as 2-methylpyridin in **44** (IC_50_ = 11 μM), which was found to be detrimental for the inhibitory potency.

Encouraged by the promising sEH inhibitor profile, we also investigated whether these compounds may still maintain FLAP inhibition using selected compounds such as the parent quinazoline-7-carboxylic acids (**10**, **22)** and quinazoline-7-carboxamides (**34**–**38**, **45, 55**) within the series (Table 2). The selected compounds were screened for inhibition of LT biosynthesis at 1 and 10 μM using a FLAP-dependent cell-based assay in intact human neutrophils.^40^ Compound **10** with a free carboxylic acid and N^3^-phenyl substitution significantly inhibited cellular LT formation only at 10 μM, while the corresponding analogue without the N^3^-phenyl (**22**) was found to be less potent at both 1 and 10 μM. Installation of a neopentyl-carboxamide function to **22** to produce **34** again showed potent inhibition of LT formation at 10 μM, although the activity was negligible at 1 μM. All other selected neopentyl amides furnished with different thiobenzyl side arm (**35**–**38**) as well as the isopropyl amide analogue (**45**) also showed inhibition of LT biosynthesis at 10 μM, although the potency was decreased. Lastly, replacement of thiobenzyl of **22** with the N-benzyl in **55** was beneficial and increased the inhibitory potency at 10 μM; however, it again lacked efficiency at 1 μM, indicating that the right and left ends of the molecules can be further explored for improved FLAP inhibition. The calculated IC_50_ values for compounds **10**, **34**, and **55** with almost complete LT biosynthesis inhibitory properties at 10 μM were between 2 and 3 μM, indicating that they are relatively weak FLAP inhibitors (Table 2). Considering that dual sEH and FLAP inhibitors may have enhanced anti-inflammatory properties, the developable pharmacological profile of these compounds as dual sEH and FLAP inhibitors may be of interest for future studies.

**Table 2:** In Vitro Inhibitory Activities of Selected Compounds toward FLAP-Mediated Cellular LT Formation^a^

cmpd.	5-LO product formation in neutrophils, remaining activity (% of control) at		IC_50_ [μM]
	1 μM	10 μM	
**10**	81.4 ± 5,8	1.3 ± 0.3	2.1 ± 0.4
**22**	102.5 ± 5.4	50.3 ± 15.5	≈10
**34**	84.5 ± 4.5	4.5 ± 1.2	2.9 ± 0.6
**35**	93.5 ± 11.4	62.1 ± 12.2	>10
**36**	89.5 ± 8.9	29.7 ± 7.2	<10
**37**	97.6 ± 4.0	20.9 ± 12.1	<10
**38**	104.8 ± 3.1	34.9 ± 10.6	<10
**45**	96.6 ± 6.7	70.3 ± 21.4	>10
**55**	77.9 ± 12.1	8.9 ± 3.6	2.7 ± 0.8

a Compounds were tested in human neutrophils challenged with 2.5 μM Ca^2+^-ionophore A23187. Data are given as percentage of control at 1 and 10 μM inhibitor concentrations (means ± SEM, *n* = 4–5). IC_50_ values for LT formation of selected compounds were obtained in intact neutrophils challenged with 2.5 μM ionophore plus 20 μM arachidonic acid.

### 2.3 Molecular Modeling

**Figure 3:**
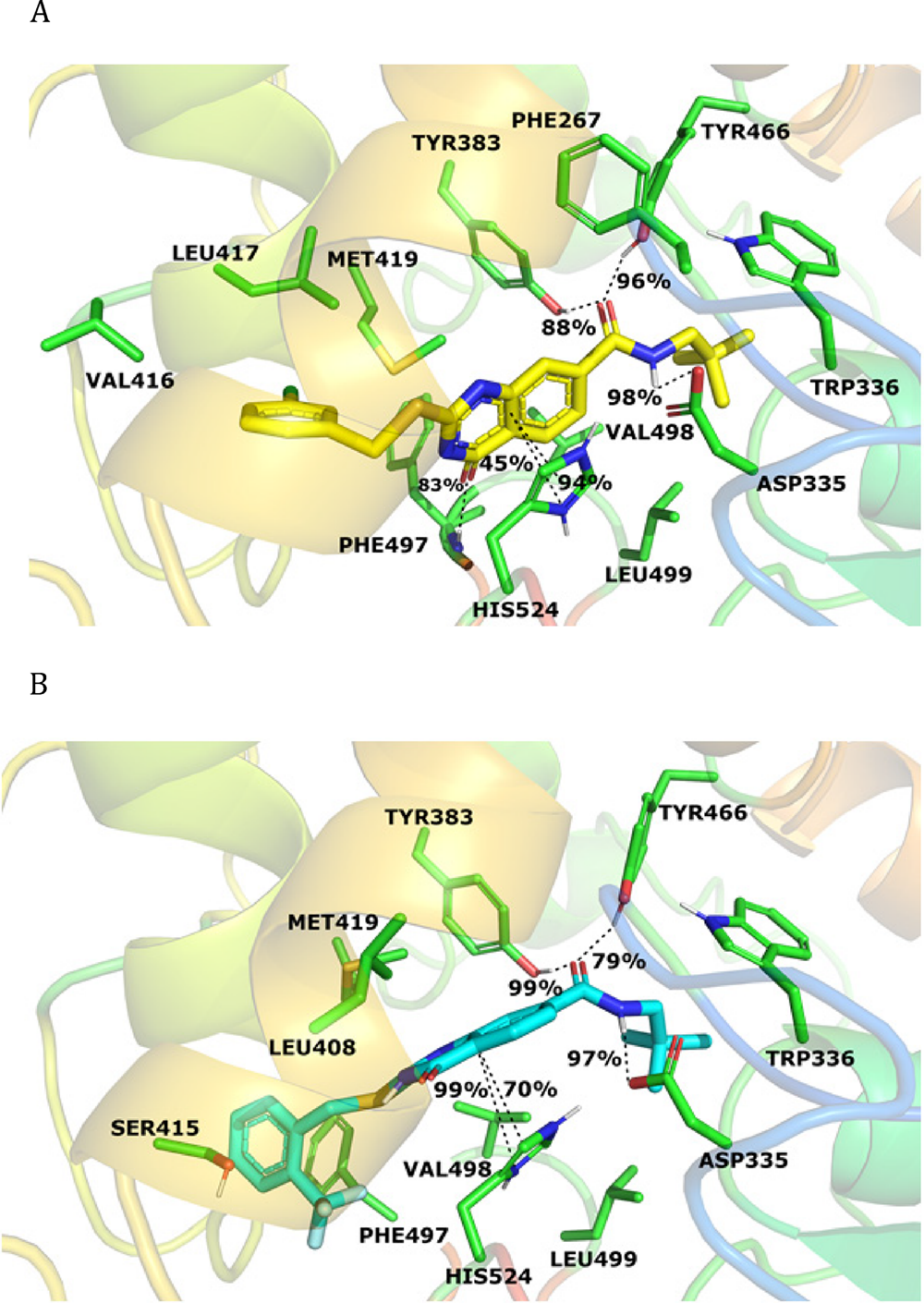
Protein–ligand interactions of (A) compound **35** and (B) compound **37** at the sEH active site with their occupancy values calculated during the simulation time of 200 ns.

A number of crystal structures of enzyme-inhibitor complexes as well as computational studies illustrate the sEH hydrolase active site as a hydrophobic L-shaped pocket with long and short branches, which are connected through a bottleneck in which the catalytic triad comprising Tyr383, Tyr566, Asp335, as well as two stabilizing His524 and Asp496 residues reside.^22,41^ In light of these reported active site properties, we performed molecular docking studies together with 200 ns molecular dynamics simulations to explore the predicted binding interactions of **35** and **37** within the sEH active site (Figure 3A, B) using the sEH crystal structure (PDB code: 6YL4).^42^

Both compounds occupy both short and long branches of the binding area with stabilizing H-bonding interactions between the amide moiety and the catalytic triad located at the bottleneck of the L-shaped hydrophobic tunnel (Figure 3A, B). As envisaged, the oxygen atom of the amide function established strong H-bonds with Tyr383 and Tyr466, while the amide NH formed H-bonding interactions with Asp335, as indicated for urea- or amide-type inhibitors.^22^ The lack of activity in tertiary amide analogues **48**–**50** highlights the importance of this hydrogen bond interaction for amide-type inhibitors at the catalytic site. Moreover, both compounds align properly with their aromatic rings in a stacking orientation to the imidazole ring of His524, a hot interaction spot for >70% of the reported sEH inhibitors,^23^ underlining the significance of the presence of an aromatic group in the close vicinity of F497, which potentially works as a gate and regulates access to the active site for bulky inhibitors. Accordingly, the quinazolinone rings of **35** and **37** bind in similar orientations at the bottleneck of the active site toward the short branch, establishing stable π–π interactions with the His524 (94% for **35**; 70% for **37**), and the binding is further stabilized by additional cation−π interactions with the same residue (45% for **35**; 99% for **37**). In addition, the 2-thiobenzyl group of the two inhibitors effectively fills the hydrophobic interior of the short branch, having van der Waals interactions with surrounding hydrophobic amino acids such as Val416, Leu417, Met419, and Phe497. Lastly, the neo-pentyl group of the amide function binds close to the bottleneck toward the Trp336 and does not fully occupy the available pocket, leaving some unused space at the end of the long branch. Therefore, this region can be considered as a potential direction that can be explored by installing relevant bulky substituents to establish new polar and/or hydrophobic interactions. These findings are in good agreement with our preliminary SAR because the compound with *i*-pr (**45**, IC_50_ = >10 μM) at the amide part caused a diminished potency as compared to the neo-pentyl counterpart (**34**, IC_50_ = 0.66 μM).

The FLAP binding interactions of **35** and **37** were also evaluated considering the essential amino acids in the binding site of the protein, as previously described for the FLAP inhibitor **5**.^30^ Because the N-benzyl ring of **5** forms strong π–π interactions with Phe123, removal of this group in **35** and **37** could be partly responsible for the activity loss against FLAP. In addition, amidification of the free carboxyl resulted in loss of ionic interactions, which was previously observed between the carboxyl group of **5** and the Lys116 at the FLAP binding site and apparently contributed to the diminished activity of **35** and **37** against FLAP. As seen in Figure S4, the quinazolinone ring of both **35** and **37** also forms π–π interactions with Phe123 (16%), which pulls the molecules away from the polar interaction area with Lys116. Therefore, compounds **35** and **37** cannot sufficiently exploit the ionic interactions with the critical polar groups available to them at the FLAP binding area, which therefore cannot complement the binding pocket adequately.

Calculated physicochemical and ADME properties of hit compound **5**, **35**, and **37** (Table S1) indicate that these compounds are expected to show good human oral and intestinal absorption profiles. While compound 5 has a higher cLogP value (>5), its derivatives have lower cLogP values (<5) and show better physicochemical profiles in agreement with Lipinski’s Rule of 5^43^ and Veber’s Rules.^44^ In addition, compounds **35** and **37** are expected to exhibit better intestinal absorption because of increased Caco-2 cell permeability values.

## 3 Conclusions

Within this work, we prepared a new series of carboxamide derivatives of the in-house FLAP inhibitor quinazolinone-7-carboxylic acid (**5**), leading to identification of novel sEH inhibitors. Our results indicate that the neopentyl group is the most effective amide substituent in these carboxamides, resulting in potent inhibition of human sEH. However, closely related carboxamides with smaller alkyl groups such as *i*-pr or *i*-bu or cyclic amines were ineffective, implying that the interactions of this peculiar amide part might be prone to future optimizations for improved sEH inhibitors. The substitution pattern of the farther phenyl at the thiobenzyl part was also crucial for controlling the inhibitory potency and can be further investigated to enhance the observed hydrophobic and/or van der Waals interactions. In addition, these compounds carry a potential to be developed as efficient FLAP inhibitors along with the sEH inhibition as demonstrated with compounds **10**, **34**, and **55**. Considering that the sEH and FLAP binding sites’ sequence order and 3D binding cavity shapes are highly different, that is, both binding sites are highly lipophilic with most of the polar residues located at the terminals of the FLAP and at the central catalytic region of sEH binding sites, the dual sEH/FLAP inhibition is reported to have higher efficacy and better safety profile, and the high sEH inhibitory potency of compounds along with the developable FLAP inhibition is encouraging and might possibly guide the development of novel candidate compounds for the treatment of inflammatory disorders and pain.

## 4 Experimental Section

### 4.1 Chemistry

All starting materials, reagents, and solvents were purchased from Sigma Aldrich Chemicals (Sigma Aldrich Corp., St. Louis, MO, USA), Merck Chemicals (Merck KGaA, Darmstadt, Germany), and ABCR (abcr GmbH, Karlsruhe, Germany). The reactions were monitored by thin layer chromatography (TLC) using Merck silica gel plates. ^1^H and ^13^C NMR spectra were recorded in DMSO-d_6_ on a Varian Mercury 400 MHz spectrometer and Bruker Avance Neo 500 MHz using tetramethylsilane as the internal standard. All chemical shifts were recorded in δ (ppm) and coupling constants were reported in Hertz. High-resolution mass spectra (HRMS) data were collected using a Waters LCT Premier XE Mass Spectrometer (high sensitivity orthogonal acceleration time-of-flight) operating using the ESI (+) or ESI (−) method, also coupled to an AQUITY Ultra-Performance Liquid Chromatography system (Waters Corporation) using a UV detector monitoring at 254 nm. Purity for all final compounds was >95%, according to the UPLC–MS method using (A) water +0.1% formic acid and (B) acetonitrile +0.1% formic acid; flow rate = 0.3 mL/min, Column: Aquity BEH C18 column (2.1 × 100 mm, 1.7 mm). Flash chromatography was performed on RediSep silica gel columns (12 g and 24 g) using a Combiflash Rf Automatic Flash Chromatography System (Teledyne-Isco, Lincoln, NE, USA) or a Reveleris PREP Purification System (Buchi, New Castle, DE, USA). Preparative chromatography was performed on Buchi US15C18HQ-250/212-C18 silica gel columns with the Reveleris PREP Purification System. Melting points of the synthesized compounds were determined using an SMP50 automated melting point apparatus (Stuart, Staffordshire, ST15 OSA, UK). Experimental data for all intermediates compounds can be found in the Supporting Information.

#### 4.1.1 2-((2-Chlorobenzyl)thio)-4-oxo-3-phenyl-3,4-dihydroquinazoline-7-carboxylic Acid (**10**)

Compound **8** (1.5 mmol, 1 eq) was dissolved in isopropanol (5 mL), and 20% NaOH solution (2 mL) was added and heated under reflux for 2 h. At the end of the time, the reaction mixture was diluted with water and acidified with acetic acid, the solid was filtered and dried. Yield 70%; mp 277.8–279.8 °C. ^1^H NMR (400 MHz, DMSO-d_6_): δ_H_ 4.53 (2H, s), 7.26–7.30 (2H, m), 7.41–7.45 (3H, m), 7.52–7.55 (3H, m), 7.64–7.66 (1H,m), 7.94 (1H, dd, *J* = 8.2, 1.4 Hz), 8.14–8.18 (2H, m); ^13^C NMR (100 MHz, DMSO-d_6_): δ_C_ 33.92, 122.48, 125.64, 127.01, 127.12, 127.26, 129.22, 129.27, 129.31, 129.43, 129.94, 131.61, 133.32, 134.07, 135.48, 136.47, 146.92, 157.71, 160.20, 166].39. HRMS *m/z* calcd for C_22_H_16_ClN_2_O_3_S [M + H]^+^ 423.0570, found: 423.0555. CAS #1053971-96-2.

#### 4.1.2 4-Oxo-3-phenyl-2-((4-(trifluoromethyl)benzyl)thio)-3,4-dihydroquinazoline-7-carboxylic Acid (**11**)

Compound **11** was synthesized according to the synthesis method in Compound **10**. Yield 92%; mp 261 °C (decomp). ^1^H NMR (400 MHz, DMSO-d_6_): δ_H_ 4.52 (2H, s), 7.46–7.49 (2H, m), 7.54–7.58 (3H, m), 7.65–7.70 (4H, m), 7.96 (1H, dd, *J* = 8.4, 1.6 Hz), 8.16–8.18 (2H, m), 13.57 (1H, bs). ^13^C NMR (100 MHz, DMSO-d_6_): δ_C_ 35.0, 122.59, 124.18 (q, ^1^*J*_C–F_ = 271.3 Hz), 125.17 (q, ^3^*J*_C–F_ = 3.8 Hz), 125.71, 127.06, 127.19, 127.73 (q, ^2^*J*_C–F_ = 32 Hz), 129.31, 129.51, 130.01, 135.59, 136.46, 142.32, 142.33, 146.98, 157.80, 160.28, 166.48. HRMS *m/z* calcd for C_22_H_16_F_3_N_2_O_3_S [M + H]^+^ 457.0834, found: 457.0816. CAS #1050945-34-0.

##### 4.1.2.1 Method A: General Synthesis Method of Quinazolinone Based Amides

The acid derivative (0.582 mmol, 1 eq) and CDI (105.2 mg, 0.64 mmol, 1.1 eq) in 3 mL of anhydrous dioxane were heated under reflux with stirring for 45 min. Amine derivate (0.8725 mmol, 1.5 eq) was then added and heated under reflux for 2 h. The mixture was diluted with water and the precipitated solid was filtered under vacuum. The crude product was purified by flash chromatography on silica gel (12 g), eluting with a gradient of 0–60% hexane in ethyl acetate or 0–60% dichloromethane in ethyl acetate.

#### 4.1.3 *N*-Isobutyl-4-oxo-3-phenyl-2-((2-chlorobenzyl)thio)-3,4-dihydroquinazoline-7-carboxamide (**12**)

It was prepared according to Method A. The crude product was purified by flash chromatography on silica gel (12 g), eluting with a gradient of 0–60% hexane in ethyl acetate. Yield 65%; mp 230.8–232.7 °C. ^1^H NMR (400 MHz, DMSO-d_6_): δ_H_ 0.93 (6H, d, *J* = 6.4 Hz), 1.90–1.93 (1H, m), 3.15 (2H, t, *J* = 6.4 Hz), 4.56 (2H, s), 7.29–7.33 (2H, m), 7.43–7.50 (3H, m), 7.55–7.57 (3H, m), 7.68–7.70 (1H, m), 7.91 (1H, dd, *J* = 8.4, 1.6 Hz), 8.15 (1H, d, *J* = 8.4 Hz), 8.18 (1H, d, *J* = 1.6 Hz), 8.86 (1H, t, *J* = 6.0 Hz). ^13^C NMR (100 MHz, DMSO-d_6_): δ_C_ 20.27, 28.06, 34.03, 46.94, 121.26, 124.52, 124.76, 124.86, 127.43, 129.37, 129.43, 129.54, 130.04, 131.79, 133.41, 133.92, 135.60, 140.42, 147.02, 157.47, 160.38, 165.17. HRMS *m/z* calcd for C_26_H_25_ClN_3_O_2_S [M + H]^+^ 478.1356, found: 478.1361.

#### 4.1.4 *N*-Isopropyl-4-oxo-3-phenyl-2-((4-(trifluoromethyl)benzyl)thio)-3,4-dihydroquinazoline-7-carboxamide (**13**)

It was prepared according to Method A. The crude product was purified by flash chromatography on silica gel (12 g), eluting with a gradient of 0–60% hexane in ethyl acetate. Yield 70%; mp 212.5–213.3 °C. ^1^H NMR (400 MHz, DMSO-d_6_): δ_H_ 1.20 (6H, d, *J* = 6.8 Hz), 4.11–4.16 (1H, m), 4.52 (2H, s), 7.44–7.47 (2H, m), 7.51–7.55 (3H, m), 7.63–7.68 (4H, m), 7.89 (1H, dd, *J* = 8.0, 1.6 Hz), 8.11–8.13 (2H, m), 8.57 (1H, d, *J* = 8.0 Hz). ^13^C NMR (100 MHz, DMSO-d_6_): δ_C_ 22.16, 34.94, 41.26, 121.13, 124.11 (q, ^1^*J*_C–F_ = 270.0 Hz), 124.49, 124.76, 125.20 (q, ^3^*J*_C–F_ = 3.8 Hz), 126.65, 127.76 (q, ^2^*J*_C–F_ = 31.5 Hz), 129.30, 129.44, 129.91, 135.57, 140.43, 142.04, 142.06, 146.90, 157.37, 160.31, 164.11. HRMS *m/z* calcd for C_26_H_23_F_3_N_3_O_2_S [M + H]^+^ 498.1463, found: 498.1453.

#### 4.1.5 *N*-Isobutyl-4-oxo-3-phenyl-2-((4-(trifluoromethyl)benzyl)thio)-3,4-dihydroquinazoline-7-carboxamide (**14**)

It was prepared according to Method A. The crude product was purified by flash chromatography on silica gel (12 g), eluting with a gradient of 0–60% hexane in ethyl acetate. Yield 50%; mp 214.7–216.6 °C. ^1^H NMR (400 MHz, DMSO-d_6_): δ_H_ 0.92 (6H, d, *J* = 6.8 Hz), 1.90 (1H, m), 3.14 (2H, t, *J* = 6.0 Hz), 4.52 (2H, s), 7.45–7.49 (2H, m), 7.53–7.59 (3H, m), 7.65–7.70 (4H, m), 7.90 (1H, dd, *J* = 8.2, 1.4 Hz), 8.13–8.15 (2H, m), 8.82 (1H, t, *J* = 6.0 Hz). ^13^C NMR (100 MHz, DMSO-d_6_): δ_C_ 20.25, 28.07, 35.08, 46.95, 121.27, 124.50, 124.77, 125.21 (q, ^1^*J*_C–F_ = 270.5 Hz), 125.27 (q, ^3^*J*_C–F_ = 3.8 Hz), 126.87, 127.84 (q, ^2^*J*_C–F_ = 31.3 Hz), 129.38, 129.56, 130.03, 130.07, 135.66, 140.44, 142.20, 147.03, 157.48, 160.41, 165.21. HRMS *m/z* calcd for C_27_H_25_F_3_N_3_O_2_S [M + H]^+^ 512.1620, found: 512.1627.

#### 4.1.6 Methyl 3-(((7-(neopentylcarbamoyl)-4-oxo-3,4-dihydroquinazolin-2-yl)thio)methyl)benzoate (**20**)

To the solution of compound **19** (0.3 mmol, 1 eq) in acetone, methyl 3-(bromomethyl)benzoate (0.37 mmol, 1.2 eq) and K_2_CO_3_ (0.92 mmol, 3 eq) were added and refluxed for 3 h. At the end of the period, the acetone was removed in vacuo and the mixture was extracted with ethyl acetate. The organic layer was dried, filtered, and evaporated. The crude product was purified by flash chromatography on silica gel (12 g), eluting with a gradient of 0–30% dichloromethane in ethyl acetate. Yield 31%; mp 269.6–271.4 °C. ^1^H NMR (400 MHz, DMSO-d_6_): δ_H_ 0.93 (9H, s), 3.15 (2H, d, *J* = 6.4 Hz), 3.84 (3H, s), 4.59 (2H, s), 7.49 (1H, t, *J* = 8.0 Hz), 7.80 (1H, d, *J* = 8.0 Hz), 7.82–7.86 (2H, m), 8.06 (1H, s), 8.08 (1H, d, *J* = 8.4 Hz), 8.18 (1H, s), 8.67 (1H, t, *J* = 6.4 Hz), 12.75 (1H, s). ^13^C NMR (100 MHz, DMSO-d_6_): δ_C_ 27.50, 32.73, 32.98, 50.23, 52.16, 121.57, 124.34, 124.84, 126.25, 128.04, 128.96, 129.66, 130.07, 134.09, 138.45, 140.62, 148.11, 155.83, 160.83, 165.84, 166.01. HRMS *m/z* calcd for C_23_H_26_N_3_O_4_S [M + H]^+^ 440.1644, found: 440.1632.

#### 4.1.7 3-(((7-(Neopentylcarbamoyl)-4-oxo-3,4-dihydroquinazolin-2-yl)thio)methyl)benzoic Acid (**21**)

It was synthesized from **19** and 3-(bromomethyl)benzoic acid according to method that was used to prepare compound 20. The crude material was purified by preparative LC, eluting with a gradient of acetonitrile in water (0–80%) to give compound **21**. Yield 42%; mp 250.6–252.6 °C. ^1^H NMR (400 MHz, DMSO-d_6_): δ_H_ 0.91 (9H, s), 3.14 (2H, d, *J* = 6.0 Hz), 4.59 (2H, s), 7.45 (1H, t, *J* = 7.2 Hz), 7.75 (1H, d, *J* = 7.2 Hz), 7.83 (2H, d, *J* = 8.0 Hz), 8.03 (1H, s), 8.09 (2H, d, *J* = 8.0 Hz), 8.66 (1H, t, *J* = 6.0 Hz). ^13^C NMR (100 MHz, DMSO-d_6_): δ_C_ 27.94, 33.16, 33.50, 50.66, 122.00, 124.80, 125.16, 126.69, 128.68, 129.18, 130.41, 131.63, 133.98, 138.44, 141.01, 148.53, 156.47, 161.38, 166.27, 167.58. HRMS *m/z* calcd for C_22_H_24_N_3_O_4_S [M + H]^+^ 426.1488, found: 426.1487.

#### 4.1.8 2-((2-Chlorobenzyl)thio)-N-neopentyl-4-oxo-3,4-dihydroquinazoline-7-carboxamide (**34**)

It was prepared according to Method A. The crude product was purified by flash chromatography on silica gel (12 g), eluting with a gradient of 0–45% hexane in ethyl acetate. Yield 30%; mp 238.4–239.8 °C. ^1^H NMR (400 MHz, DMSO-d_6_): δ_H_ 0.90 (9H, s), 3.13 (2H, d, *J* = 6.4 Hz), 4.60 (2H,s), 7.28–7.33 (2H, m), 7.46–7.49 (1H, m), 7.68–7.70 (1H, m), 7.81 (1H, dd, *J* = 8.0, 1.0 Hz), 8.05–9.08 (2H, m), 8.64 (1H, t, *J* = 6.4 Hz), 12.72 (1H, bs); ^13^C NMR (100 MHz, DMSO-d_6_): δ_C_ 27.95, 32.09, 33.16, 50.67, 122.01, 124.87, 125.27, 126.70, 127.87, 129.92, 130.00, 132.10, 133.85, 134.98, 141.05, 148.54, 156.10, 161.25, 166.26; HRMS *m/z* calcd for C_21_H_23_ClN_3_O_2_S [M + H]^+^ 416.1200, found: 416.1205.

#### 4.1.9 2-((3-Chlorobenzyl)thio)-N-neopentyl-4-oxo-3,4-dihydroquinazoline-7-carboxamide (**35**)

It was prepared according to Method A. The crude product was purified by flash chromatography on silica gel (12 g), eluting with a gradient of 0–50% dichloromethane in ethyl acetate. Yield 44%; mp 217.1–218.4 °C. ^1^H NMR (400 MHz, DMSO-d_6_): δ_H_ 0.91 (9H, s), 3.14 (2H, d, *J* = 6.4 Hz), 4.52 (2H, s), 7.30–7.37 (2H, m), 7.47 (1H, d, *J* = 7.2 Hz), 7.59 (1H, s), 7.83 (1H, dd, *J* = 8.4, 1.2 Hz), 8.04 (1H, d, *J* = 1.2 Hz), 8.08 (1H, d, *J* = 8.4 Hz), 8.65 (1H, t, *J* = 6.4 Hz), 12.73 (1H, s). ^13^C NMR (100 MHz, DMSO-d_6_): δ_C_ 27.41, 32.63, 32.72, 50.12, 121.50, 124.31, 124.66, 126.16, 127.18, 127.76, 128.98, 130.23, 132.78, 140.02, 140.51, 148.01, 155.72, 160.71, 165.70. HRMS *m/z* calcd for C_21_H_23_ClN_3_O_2_S [M + H]^+^ 416.1200, found: 416.1216.

#### 4.1.10 2-((4-Chlorobenzyl)thio)-N-neopentyl-4-oxo-3,4-dihydroquinazoline-7-carboxamide (**36**)

It was prepared according to Method A. The crude product was purified by flash chromatography on silica gel (12 g), eluting with a gradient of 0–60% dichloromethane in ethyl acetate. Yield 51%; mp 220.0–222.0 °C. ^1^H NMR (400 MHz, DMSO-d_6_): δ_H_ 0.90 (9H, s), 3.12 (2H, d, *J* = 6.4 Hz), 4.05 (2H, s), 7.36 (2H, d, *J* = 8.4 Hz), 7.50 (2H, d, *J* = 8.4 Hz), 7.81 (1H, d, *J* = 8.2 Hz), 8.01 (1H, s), 8.07 (1H, d, *J* = 8.2 Hz), 8.57–8.60 (1H, m), 12.67 (1H, s). ^13^C NMR (100 MHz, DMSO-d_6_): δ_C_ 27.41, 32.58, 32.64, 50.16, 121.47, 124.25, 124.70, 126.12, 128.32, 130.86, 131.84, 136.51, 140.51, 148.03, 155.78, 160.72, 165.72. HRMS *m/z* calcd for C_21_H_23_ClN_3_O_2_S [M + H]^+^ 416.1200, found: 416.1191.

#### 4.1.11 2-((2-(Trifluoromethyl)benzyl)thio)-N-Neopentyl-4-oxo-3,4-dihydroquinazoline-7-carboxamide (**37**)

It was prepared according to Method A. The crude product was purified by flash chromatography on silica gel (12 g), eluting with a gradient of 0–40% dichloromethane in ethyl acetate. Yield 45%; mp 217.9–219.3 °C. ^1^H NMR (400 MHz, DMSO-d_6_): δ_H_ 0.91 (9H, s), 3.14 (2H, d, *J* = 6.4 Hz), 4.71 (2H, s), 7.52 (1H, t, *J* = 7.6 Hz), 7.66 (1H, t, *J* = 7.6 Hz), 7.76 (1H, d, *J* = 7.6 Hz), 7.84 (2H, d, *J* = 8.0 Hz), 8.03 (1H, s), 8.10 (1H, d, *J* = 8.0 Hz), 8.66 (1H, t, *J* = 6.4 Hz), 12.77 (1H, s). ^13^C NMR (100 MHz, DMSO-d_6_): δ_C_ 27.41, 30.17, 32.63, 50.14, 121.53, 124.30 (q, ^1^*J*_C–F_ = 272 Hz), 124.43, 124.73, 126.11 (q, ^3^*J*_C–F_ = 5.6 Hz), 127.15 (q, ^2^*J*_C–F_ = 30 Hz), 128.23, 128.36, 131.94, 132.98, 135.11 (q, ^4^*J*_C–F_ = 1.3 Hz), 140.55, 147.94, 155.42, 160.72, 165.72. HRMS *m/z* calcd for C_22_H_23_F_3_N_3_O_2_S [M + H]^+^ 450.1463, found: 450.1461.

#### 4.1.12 2-((3,4-Dichlorobenzyl)thio)-N-neopentyl-4-oxo-3,4-dihydroquinazoline-7-carboxamide (**38**)

It was prepared according to Method A. The crude product was purified by flash chromatography on silica gel (12 g), eluting with a gradient of 0–40% dichloromethane in ethyl acetate. Yield 54%; mp 215.5–216.9 °C. ^1^H NMR (500 MHz, DMSO-d_6_): δ_H_ 0.93 (9H, s), 3.15 (2H, d, *J* = 6.4 Hz), 4.52 (2H, s), 7.51 (1H, dd, *J* = 8.3, 2.0 Hz), 7.59 (1H, d, *J* = 8.3 Hz), 7.81–7.85 (2H, m), 8.04 (1H, d, *J* = 1.4 Hz), 8.09 (1H, d, *J* = 8.3 Hz), 8.66 (1H, m), 12.75 (1H, s). ^13^C NMR (125 MHz, DMSO-d_6_): δ_C_ 27.96, 32.68, 33.19, 50.67, 122.06, 124.87, 125.24, 126.72, 130.00, 130.33, 131.05, 131.23, 131.74, 139.52, 141.09, 148.53, 156.14, 161.27, 166.27. HRMS *m/z* calcd for C_21_H_22_Cl_2_N_3_O_2_S [M + H]^+^ 450.0810, found: 450.0794.

#### 4.1.13 2-((2-Fluorobenzyl)thio)-N-neopentyl-4-oxo-3,4-dihydroquinazoline-7-carboxamide (**39**)

It was prepared according to Method A. The crude product was purified by flash chromatography on silica gel (12 g), eluting with a gradient of 0–40% dichloromethane in ethyl acetate. Yield 55%; mp 210.3–212.2 °C. ^1^H NMR (500 MHz, DMSO-d_6_): δ_H_ 0.93 (9H, s), 3.15 (2H, d, *J* = 6.2 Hz), 4.56 (2H, s), 7.16–7.24 (2H, m), 7.34–7.35 (1H, m), 7.63 (1H, td, *J* = 7.7, 1.4 Hz), 7.84 (1H, dd, *J* = 8.2, 1.5 Hz), 8.05 (1H, m), 8.09 (1H, d, *J* = 8.2 Hz), 8.66–8.68 (1H, t, *J* = 6.2 Hz), 12.75 (1H, s). ^13^C NMR (125 MHz, DMSO-d_6_): δ_C_ 27.64, 27.66, 27.98, 50.70, 115.86 (d, ^2^*J*_C–F_ = 21.0 Hz), 122.04, 124.49 (d, ^2^*J*_C–F_ = 14.3 Hz), 124.88, 125.00 (d, ^4^*J*_C–F_ = 3.3 Hz), 125.29, 126.72, 130.22 (d, ^3^*J*_C–F_ = 8.1 Hz), 132.00 (d, ^3^*J*_C–F_ = 3.4 Hz), 141.08, 148.57, 156.10, 160.96 (d, ^1^*J*_C–F_ = 244.3 Hz), 161.29, 166.31. HRMS *m/z* calcd for C_21_H_23_FN_3_O_2_S [M + H]^+^ 400.1495, found: 400.1501.

#### 4.1.14 2-((2-Cyanobenzyl)thio)-N-neopentyl-4-oxo-3,4-dihydroquinazoline-7-carboxamide (**40**)

It was prepared according to Method A. The crude product was purified by flash chromatography on silica gel (12 g), eluting with a gradient of 0–50% dichloromethane in ethyl acetate. Yield 40%; mp 190.3–192.2 °C. ^1^H NMR (500 MHz, DMSO-d_6_): δ_H_ 0.93 (9H, s), 3.15 (2H, d, *J* = 6.4 Hz), 4.69 (2H, s), 7.48 (1H, td, *J* = 7.7, 0.8 Hz), 7.68 (1H, td, *J* = 7.7, 1.2 Hz), 7.81 (2H, d, *J* = 8.2 Hz), 7.86 (1H, dd, *J* = 7.7, 0.8 Hz), 8.09 (2H, d, *J* = 8.2 Hz), 8.64 (1H, t, *J* = 6.4 Hz), 12.78 (1H, s). ^13^C NMR (125 MHz, DMSO-d_6_): δ_C_ 27.96, 32.45, 33.16, 50.68, 112.54, 118.01, 122.02, 124.86, 125.42, 126.71, 128.81, 131.11, 133.52, 133.84, 141.28, 141.50, 148.52, 155.62, 161.24, 166.45. HRMS *m/z* calcd for C_22_H_23_N_4_O_2_S [M + H]^+^ 407.1542, found: 407.1554.

#### 4.1.15 2-((2-Methylbenzyl)thio)-N-neopentyl-4-oxo-3,4-dihydroquinazoline-7-carboxamide (**41**)

It was prepared according to Method A. The crude product was purified by flash chromatography on silica gel (12 g), eluting with a gradient of 0–50% dichloromethane in ethyl acetate. Yield 62%; mp 207.4–209.4 °C. ^1^H NMR (500 MHz, DMSO-d_6_): δ_H_ 0.93 (9H, s), 2.40 (3H, s), 3.15 (2H, d, *J* = 6.3 Hz), 4.54 (2H, s), 7.16–7.23 (3H, m), 7.47 (1H, d, *J* = 7.5 Hz), 7.84 (1H, dd, *J* = 8.2, 1.6 Hz), 8.06 (1H, s), 8.10 (1H, d, *J* = 8.2 Hz), 8.67 (1H, t, *J* = 6.3 Hz), 12.71 (1H, s). ^13^C NMR (125 MHz, DMSO-d_6_): δ_C_ 19.34, 27.98, 32.51, 33.18, 50.71, 122.06, 124.84, 125.25, 126.60, 126.72, 128.25, 130.57, 130.80, 134.77, 137.16, 141.05, 148.67, 156.60, 161.29, 166.32. HRMS *m/z* calcd for C_22_H_26_N_3_O_2_S [M + H]^+^ 396.1746, found: 396.1750.

#### 4.1.16 2-((2-Methoxybenzyl)thio)-N-neopentyl-4-oxo-3,4-dihydroquinazoline-7-carboxamide (**42**)

It was prepared according to Method A. The crude product was purified by flash chromatography on silica gel (12 g), eluting with a gradient of 0–50% dichloromethane in ethyl acetate. Yield 32%; mp 230.2–232.1 °C. ^1^H NMR (400 MHz, DMSO-d_6_): δ_H_ 0.93 (9H, s), 3.16 (2H, d, *J* = 6.2 Hz), 3.85 (3H, s), 4.49 (2H, s), 6.91 (1H, td, *J* = 7.6, 0.8 Hz), 7.03 (1H, d, *J* = 7.6 Hz), 7.29 (1H, dd, *J* = 8.0, 1.6 Hz), 7.49 (1H, dd, *J* = 7.2, 1.6 Hz), 7.83 (1H, dd, *J* = 8.4, 1.6 Hz), 8.08–8.11 (2H, m), 8.69 (1H, t, *J* = 6.2 Hz), 12.67 (1H, s). ^13^C NMR (100 MHz, DMSO-d_6_): δ_C_ 27.51, 28.82, 32.74, 50.22, 55.56, 110.97, 120.32, 121.51, 124.31, 124.34, 124.75, 126.23, 129.19, 130.52, 140.51, 148.21, 156.51, 157.25, 160.83, 165.81. HRMS *m/z* calcd for C_22_H_26_N_3_O_3_S [M + H]^+^ 412.1695, found: 412.1688.

#### 4.1.17 2-((2-(Trifluoromethoxy)benzyl)thio)-N-neopentyl-4-oxo-3,4-dihydroquinazoline-7-carboxamide (**43**)

It was prepared according to Method A. The crude product was purified by flash chromatography on silica gel (12 g), eluting with a gradient of 0–40% dichloromethane in ethyl acetate. Yield 57%; mp 220.9–221.9 °C. ^1^H NMR (500 MHz, DMSO-d_6_): δ_H_ 0.93 (9H, s), 3.14 (2H, d, *J* = 6.3 Hz), 4.61 (2H, s), 7.37–7.44 (3H, m), 7.73 (1H, td, *J* = 7.7, 1.6 Hz), 7.83 (1H, dd, *J* = 8.2, 1.6 Hz), 8.02 (1H, s), 8.10 (1H, d, *J* = 8.2 Hz), 8.65 (1H, t, *J* = 6.3 Hz), 12.75 (1H, s). ^13^C NMR (125 MHz, DMSO-d_6_): δ_C_ 27.94, 28.40, 33.17, 50.68, 120.60 (^1^*J*_C–F_ = 255 Hz), 120.82, 122.02, 123.73, 124.86, 125.29, 126.72, 128.00, 130.08, 132.30, 141.16, 147.30, 148.52, 155.99, 161.28, 166.37. HRMS *m/z* calcd for C_22_H_23_F_3_N_3_O_3_S [M + H]^+^ 466.1412, found: 466.1414.

#### 4.1.18 2-(((2-Methylpyridin-3-yl)methyl)thio)-N-neopentyl-4-oxo-3,4-dihydroquinazoline-7-carboxamide (**44**)

It was prepared according to Method A. The crude product was purified by flash chromatography on silica gel (12 g), eluting with a gradient of 0–50% dichloromethane in ethyl acetate. Yield 52%; mp 202.3–203.8 °C. ^1^H NMR (500 MHz, DMSO-d_6_): δ_H_ 0.93 (9H, s), 2.61 (3H, s), 3.15 (2H, d, *J* = 6.3 Hz), 4.56 (2H, s), 7.19 (1H, dd, *J* = 7.7, 4.8 Hz), 7.83 (1H, dd, *J* = 8.2, 1.6 Hz), 7.87 (1H, dd, *J* = 7.7, 1.6 Hz), 8.04 (1H, s), 8.10 (1H, d, *J* = 8.2 Hz), 8.35 (1H, dd, *J* = 4.8, 1.6 Hz), 8.66 (1H, d, *J* = 6.3 Hz), 12.76 (1H, s). ^13^C NMR (125 MHz, DMSO-d_6_): δ_C_ 22.57, 27.98, 31.59, 33.19, 50.69, 121.92, 122.07, 124.87, 125.26, 126.73, 130.85, 138.00, 141.10, 148.33, 148.67, 156.21, 157.26, 161.26, 166.30. HRMS *m/z* calcd for C_21_H_25_N_4_O_2_S [M + H]^+^ 397.1698, found: 397.1684.

#### 4.1.19 2-((2-Chlorobenzyl)thio)-N-isopropyl-4-oxo-3,4-dihydroquinazoline-7-carboxamide (**45**)

Step 1: Compound **22** (0.346 mmol, 1 eq) in SOCl_2_ (2 mL) was refluxed for 3 h. The reaction mixture was then concentrated in vacuo. The obtained 2-((2-chlorobenzyl)thio)-4-oxo-3,4-dihydroquinazoline-7-carbonyl chloride was used in the next step. Step 2: Isopropylamine (0.346 mmol, 1 eq) was dissolved in DCM (3 mL), Et_3_N (0.865 mmol, 2.5 eq), and 2-((2-chlorobenzyl)thio)-4-oxo-3,4-dihydroquinazoline-7-carbonyl chloride (0.346 mmol, 1 eq) were added and stirred for 4 h under nitrogen gas. Distilled water was added to the reaction mixture and extracted with DCM, organic phase-dried over Na_2_SO_4_, and evaporated. The crude product was purified by flash chromatography on silica gel (12 g), eluting with a gradient of 0–10% dichloromethane in methanol. Yield 15%; mp 306.3 °C (decomp). ^1^H NMR (500 MHz, DMSO-d_6_): δ_H_ 1.20 (6H, d, *J* = 6.55 Hz), 4.12–4.16 (1H, m), 4.64 (2H, s), 7.33–7.36 (2H, m), 7.50–7.52 (1H, m), 7.69–7.72 (1H, m), 7.84 (1H, dd, *J* = 8.2, 1.5 Hz), 8.08 (2H, d, *J* = 8.4 Hz), 8.54 (1H, d, *J* = 7.7 Hz), 12.7 (1H, s); ^13^C NMR (125 MHz, DMSO-d_6_): δ_C_ 22.71, 32.07, 41.76, 122.02, 124.82, 125.23, 126.68, 127.94, 129.96, 130.01, 132.07, 133.86, 134.99, 140.88, 148.57, 156.13, 161.30, 164.82; HRMS *m/z* calcd for C_19_H_19_ClN_3_O_2_S [M + H]^+^ 388.1088, found: 388.1080.

#### 4.1.20 2-((4-(Trifluoromethyl)benzyl)thio)-N-isobutyl-4-oxo-3,4-dihydroquinazoline-7-carboxamide (**46**)

It was prepared according to Method A. The crude product was purified by flash chromatography on silica gel (12 g), eluting with a gradient of 0–25% dichloromethane in ethyl acetate. Yield 38%; mp 262.8–264.6 °C. ^1^H NMR (400 MHz, DMSO-d_6_): δ_H_ 0.88 (6H, d, *J* = 6.4 Hz), 1.82–1.89 (1H, m), 3.10 (2H, t, *J* = 6.6 Hz), 4.59 (2H, s), 7.66–7.72 (4H, m), 7.81 (1H, dd, *J* = 8.6, 1.4 Hz), 8.02 (1H, s), 8.06 (1H, d, *J* = 8.4 Hz), 8.73 (1H, t, *J* = 5.8 Hz), 12.73 (1H, bs); ^13^C NMR (100 MHz, DMSO-d_6_): δ_C_ 20.19, 28.01, 32.72, 46.86, 121.58, 124.26 (q, ^1^*J*_C–F_ = 270.0 Hz), 124.28, 124.69, 125.22 (q, ^3^*J*_C–F_ = 3.8 Hz), 126.25, 127.65 (q, ^2^*J*_C–F_ = 31.4 Hz), 129.88, 140.29, 142.66, 148.07, 155.69, 160.78, 165.25; HRMS *m/z* calcd for C_21_H_21_F_3_N_3_O_2_S [M + H]^+^ 436.1307, found: 436.1306.

#### 4.1.21 2-((2-Chlorobenzyl)thio)-7-(piperazine-1-carbonyl)quinazolin-4(3*H*)-one (**48**)

It was prepared according to Method A. The crude product was purified by flash chromatography on silica gel (12 g), eluting with a gradient of 0–15% dichloromethane in methanol. Yield 64%; mp 159.9–162.0 °C. ^1^H NMR (500 MHz, DMSO-d_6_): δ_H_ 2.73 (2H, bs), 2.84 (2H, bs), 3.28 (2H, bs), 3.63 (2H, bs), 4.58 (2H, s), 7.30–7.32 (2H, m), 7.35 (1H, dd, *J* = 8.1, 1.5 Hz), 7.48–7.50 (1H, m), 7.57 (1H, d, *J* = 1.5 Hz), 7.73–7.75 (1H, m), 8.05 (1H, d, *J* = 8.1 Hz). ^13^C NMR (125 MHz, DMSO-d_6_): δ_C_ 30.07, 31.16, 32.11, 120.89, 123.87, 124.18, 127.06, 127.77, 129.81, 129.83, 132.27, 133.85, 135.50, 142.19, 148.89, 157.71, 162.21, 168.29. HRMS *m/z* calcd for C_20_H_20_ClN_4_O_2_S [M + H]^+^ 415.0996, found: 415.1005.

#### 4.1.22 2-((2-Chlorobenzyl)thio)-7-(piperidine-1-carbonyl)quinazolin-4(3*H*)-one) (**49**)

It was prepared according to Method A. The crude product was purified by flash chromatography on silica gel (12 g), eluting with a gradient of 0–60% dichloromethane in ethyl acetate. Yield 30%; mp 175.6–177.2 °C. ^1^H NMR (500 MHz, DMSO-d_6_): δ_H_ 1.48–1.64 (6H, m), 3.26 (2H, bs), 3.63 (2H, bs), 4.60 (2H, s), 7.29–7.38 (2H, m), 7.37 (1H, dd, *J* = 8.0, 1.5 Hz), 7.48–7.50 (1H, m), 7.58 (1H, s), 7.73–7.75 (1H, m), 8.07 (1H, d, *J* = 8.0 Hz), 12.71 (1H, s). ^13^C NMR (125 MHz, DMSO-d_6_): δ_C_ 24.47, 25.67, 26.37, 32.13, 42.38, 48.38, 120.68, 124.11, 127.09, 127.79, 129.86, 129.91, 132.32, 133.89, 135.24, 143.10, 148.61, 156.32, 161.20, 168.00. HRMS *m/z* calcd for C_21_H_21_ClN_3_O_2_S [M + H]^+^ 414.1043, found: 414.1049.

#### 4.1.23 2-((2-Chlorobenzyl)thio)-7-(morpholine-4-carbonyl)quinazolin-4(3*H*)-one (**50**)

It was prepared according to Method A. The crude product was purified by flash chromatography on silica gel (12 g), eluting with a gradient of 0–60% dichloromethane in ethyl acetate. Yield 44%; mp 198.6–200.4 °C. ^1^H NMR (400 MHz, DMSO-d_6_): δ_H_ 3.33–3.68 (8H, m), 4.59 (2H, s), 7.28–7.33 (2H, m), 7.40 (1H, dd, *J* = 8.4, 1.2 Hz), 7.46–7.49 (1H, m), 7.62 (1H, s), 7.71–7.73 (1H, m), 8.07 (1H, d, *J* = 8.4 Hz), 12.67 (1H, s). ^13^C NMR (100 MHz, DMSO-d_6_): δ_C_ 31.62, 65.93, 120.37, 123.83, 124.04, 126.55, 127.25, 129.32, 129.35, 131.71, 133.33, 134.62, 141.65, 148.07, 155.83, 160.61, 167.73. HRMS *m/z* calcd for C_20_H_19_ClN_3_O_3_S [M + H]^+^ 416.0836, found: 416.0841.

#### 4.1.24 2-((2-Chlorobenzyl)amino)-N-neopentyl-4-oxo-3,4-dihydroquinazoline-7-carboxamide (**55**)

It was prepared according to Method A. The crude product was purified by flash chromatography on silica gel (12 g), eluting with a gradient of 0–65% dichloromethane in ethyl acetate. Yield %; mp 176.7–178.7 °C. ^1^H NMR (500 MHz, DMSO-d_6_): δ_H_ 0.89 (9H, s), 3.09 (2H, d, *J* = 6.3 Hz), 4.67 (2H, d, *J* = 5.6 Hz), 6.85 (1H, t, *J* = 5.6 Hz), 7.30–7.36 (2H, m), 7.45–7.49 (2H m), 7.53 (1H dd, *J* = 8.2, 1.5 Hz), 7.71 (1H, d, *J* = 1.5 Hz), 7.95 (1H, d, *J* = 8.2 Hz), 8.52 (1H, t, *J* = 6.3 Hz), 11.17 (1H, s). ^13^C NMR (125 MHz, DMSO-d_6_): δ_C_ 27.98, 33.16, 42.12, 50.60, 119.61, 121.25, 123.82, 126.48, 127.77, 129.24, 129.29, 129.68, 132.58, 136.79, 140.69, 151.10, 151.28, 162.04, 166.62. HRMS *m/z* calcd for C_21_H_24_ClN_4_O_2_ [M + H]^+^ 399.1588, found: 399.1577.

### 4.2 Biology

#### 4.2.1 Isolation and Culture of Human Cells

Neutrophils were isolated from leukocyte concentrates obtained from freshly withdrawn peripheral blood of healthy adult male and female donors which were provided by the Institute of Transfusion Medicine, University Hospital Jena, Germany. The experimental protocol was approved by the ethical committee of the University Hospital Jena, and all methods were performed in accordance with the relevant guidelines and regulations. The leukocyte concentrates were mixed with dextran (from Leuconostoc spp. MW ∼40,000, Sigma Aldrich) for sedimentation of erythrocytes, and the supernatant was centrifuged on lymphocyte separation medium (Histopaque-1077, Sigma Aldrich). Contaminating erythrocytes in the pelleted neutrophils were removed by hypotonic lysis using distilled water. The neutrophils were washed twice in ice-cold PBS pH 7.4 and finally resuspended in PBS pH 7.4 plus 1 mg/mL glucose (PGC buffer).

#### 4.2.2 Determination of FLAP-Dependent 5-LO Product Formation in Intact Neutrophils

Human neutrophils [5 × 10^6^; in PGC buffer; incubation volume, 1 mL] were preincubated with 0.1% DMSO (vehicle) or with the compounds at 37 °C for 15 min. After addition of 2.5 μM A23187, the reaction was incubated for 10 min at 37 °C and then stopped by the addition of 1 mL of methanol, and 30 μL of 1 N HCl plus 200 ng of PGB_1_ and 500 μL of PBS were added. Samples were then subjected to solid-phase extraction on C18-columns (100 mg, UCT, Bristol, PA), and 5-LO products (LTB_4_ and its trans-isomers, and 5-H(*p*)ETE) were extracted and analyzed in the presence of internal standard PGB_1_ by RP-HPLC and UV detection as reported elsewhere.^45^

#### 4.2.3 sEH Assay

Human recombinant sEH was expressed and purified as reported before.^46^ In brief, Sf9 cells were infected with a recombinant baculovirus (kindly provided by Dr. B. Hammock, University of California, Davis, CA). Seventy-two hours post-transfection, cells were pelleted and sonicated (3 × 10 s at 4 °C) in lysis buffer containing NaHPO_4_ (50 mM, pH 8), NaCl (300 mM), glycerol (10%), EDTA (1 mM), phenylmethanesulfonyl fluoride (1 mM), leupeptin (10 μg/mL), and a soybean trypsin inhibitor (60 μg/mL). Supernatants after centrifugation at 100,000 × *g* (60 min, 4 °C) were subjected to benzylthio-sepharose-affinity chromatography in order to purify sEH by elution with 4-fluorochalcone oxide in PBS containing 1 mM DTT and 1 mM EDTA. Dialyzed and concentrated (Millipore Amicon-Ultra-15 centrifugal filter) enzyme solution was assayed for total protein with a Bio-Rad protein detection kit (Bio-Rad Laboratories, Munich, Germany), and the epoxide hydrolase activity was determined by using a fluorescence-based assay as described before.^47^ Shortly, sEH was diluted in Tris buffer (25 mM, pH 7) supplemented with BSA (0.1 mg/mL) to an appropriate enzyme concentration and preincubated with test compounds or vehicle (0.1% DMSO) for 10 min at room temperature (RT). The reaction was started by the addition of 50 μM 3-phenyl-cyano(6-methoxy-2-naphthalenyl)methyl ester-2-oxiraneacetic acid (PHOME), a nonfluorescent compound that is enzymatically converted into fluorescent 6-methoxy-naphtaldehyde at RT. After 60 min, reactions were stopped by ZnSO_4_ (200 mM) and fluorescence was detected (λem 465 nm, λex 330 nm) using a NOVOstar microplate reader (BMG Labtech GmbH, Ortenberg, Germany), and potential fluorescence of test compounds was subtracted from the read-out, if required.

### 4.3 Molecular Modeling

The docking approach was applied to obtain putative binding poses of compounds **35** and **37** within the sEH active site (PDB code 6YL4)^42^ located at the C-terminal of the enzyme. The recently released crystal structure was selected because of having good resolution (2.00 Å), co-crystallization with a small molecule inhibitor (not fragment), and reproducibility of the binding modes of the crystallized ligands by rebuilding them and cross-docking among all published sEH crystals. Those ligands were excluded from the binding site and re-docked to the same location with a high similarity. The RMSD value on heavy ligand atoms of the co-crystallized ligand of the selected crystal structure was found to be 0.48 by comparing the co-crystallized and docked poses. Similarly, binding poses of both compounds were computationally retrieved by docking within the FLAP binding site (PDB code 6WGC).^48^ The used crystal exhibits the best resolution within published FLAP crystals, giving reproducible results with docking. Structures were drawn by using Maestro.^49^ Atom types and protonation states of ligands and proteins were assigned at pH 7.0 ± 2.0 with OPLS4 forcefield. The *LigPrep*(50) routine was applied to prepare the ligands and *Protein Preparation Wizard*(51) was utilized to prepare the enzyme and add predicted positions of its missing side chains. Van der Waals radius scaling factor and partial charge cutoff values were used with their default parameters, 1.0 and 0.25. The simulations were done with *Glide* in standard precision mode^52,53^ and only the highest scoring poses were kept for visualization and further studies. Top scored poses were issued to molecular dynamics to see time-dependent interaction patterns of the molecules during 200 ns simulations with four copies. The simulation systems were prepared with *System Builder* and simulations were run with *Desmond*.^54^ The SPC model was used for waters. Additionally, POPC membrane atoms were used for embedding FLAP within the membrane. The long-range Coulombic interactions cutoff value was set to 9.0 Å. The systems were neutralized with 6 Na^+^ ions. The simulation system was prepared with the OPLS4 forcefield.

#### 4.3.1 Molecular Dynamics Simulations

The simulations were relaxed with five stages for the simulations conducted with sEH, and an additional membrane relaxation step was applied for FLAP. (i) The first step was utilized with the NVT ensemble and Brownian dynamics, the temperature used was 10 K for 12 ps, the relaxation included 2000 minimization steps, and there was existence of harmonic restraints on the solute atoms by applying force constant for 50 kcal/mol/Å^2^. (ii) The second stage proceeded with the same parameters by utilizing the NVT ensemble and the Langevin method and (iii) the third step continued with utilizing the NPT ensemble with the Langevin method. (iv) At the fourth step, previous system’s temperature was increased to the simulation temperature (300 K). (v) At the fifth and last relaxation steps, restraints were excluded, and the system was relaxed for 24 ps. Then, 200 ns of MD simulation was started by setting the recording interval to 200 picoseconds for both saving the trajectories and energy values. The most populated trajectory was selected by the *Desmond Trajectory Clustering* panel, and image generation was done with *PyMOL*.^55^ Molecular dynamics of each complex showed similar binding patterns for each simulation copy; therefore, occupancy values of the first simulation are presented. The reported occupancies are higher than 10%.

## Supporting Information Available

Representative NMR spectra and graphical presentations of bioactivity assays for the compounds tested; RMSD figures of compounds **35** and **37** simulated with sEH and FLAP by MD simulations (PDF)
