# Supplementary material for: Quinazoline-4(3H)-one-7-carboxamide Derivatives as Human Soluble Epoxide Hydrolase Inhibitors with Developable 5-Lipoxygenase Activating Protein Inhibition
Source: ACS Omega. 2022 Oct 5;7(41):36354–65. doi: 10.1021/acsomega.2c04039 (PMC9583330; doi:10.1021/acsomega.2c04039)
Supplement: Supplementary file 1 — ao2c04039_si_001.pdf [file ao2c04039_si_001.pdf]

Supplementary Information

Quinazoline-4(3H)-one-7-carboxamide Derivatives as Human Soluble Epoxide Hydrolase (sEH) Inhibitors with Developable 5-Lipoxygenase Activating Protein (FLAP) Inhibition

Sümeyye Turanlı,[¥,§] Azize Gizem Ergül,[¥,‡] Paul M. Jordan,[†] Abdurrahman Olğaç,[¥] Burcu Çalışkan,[¥] Oliver Werz,[†] Erden Banoglu[¥,*]

[¥]Department of Pharmaceutical Chemistry, Faculty of Pharmacy, Gazi University, Taç Sok. No:3 Yenimahalle, 06560 Ankara, Turkey

[†]Department of Pharmaceutical/Medicinal Chemistry, Institute of Pharmacy, Friedrich Schiller University Jena, Philosophenweg 14, D-7743 Jena, Germany

[§] Department of Pharmaceutical Chemistry, Faculty of Pharmacy, Adıyaman University, Adıyaman, Turkey.
[‡] Department of Pharmaceutical Chemistry, Faculty of Pharmacy, Lokman Hekim University, Ankara, Turkey

Table of Contents

Synthesis of Intermediate Compounds S2
Figure S2. Representative bioactivity results of the selected final compounds. S9
Figure S3. RMSD values of compounds A) 35 and B) 37 simulated with sEH. Both figures were plotted after fitting each frame against the first one. S9
Figure S4 Protein-ligand interactions of A) compound 35 and B) compound 37 at FLAP binding site with their occupancy values calculated during the simulation time of 200 ns. S9
Figure S5. RMSD values of compounds A) 35 and B) 37 simulated with FLAP. Both figures were plotted after fitting each frame against the first one. S10
Figure S6. ^{1}H-NMR, ^{13}C-NMR and HRMS spectrums of 10 .... S11
Figure S7. ^{1}H-NMR, ^{13}C-NMR and HRMS spectrums of 11 .... S14
Figure S8. ^{1}H-NMR, ^{13}C-NMR and HRMS spectrums of 12 .... S17
Figure S9. ^{1}H-NMR, ^{13}C-NMR and HRMS spectrums of 13 .... S20
Figure S10. ^{1}H-NMR, ^{13}C-NMR and HRMS spectrums of 14 .... S23
Figure S11. ^{1}H-NMR, ^{13}C-NMR and HRMS spectrums of 20 .... S26
Figure S12. ^{1}H-NMR, ^{13}C-NMR and HRMS spectrums of 21 .... S29
Figure S13. ^{1}H-NMR, ^{13}C-NMR and HRMS spectrums of 34 .... S32
Figure S14. ^{1}H-NMR, ^{13}C-NMR and HRMS spectrums of 35 .... S35
Figure S15. ^{1}H-NMR, ^{13}C-NMR and HRMS spectrums of 36 .... S38
Figure S16. ^{1}H-NMR, ^{13}C-NMR and HRMS spectrums of 37 .... S41
Figure S17. 1H-NMR, 13C-NMR and HRMS spectrums of 38 .... S44
Figure S18. ^{1}H-NMR, ^{13}C-NMR and HRMS spectrums of 39 .... S47
Figure S19. ^{1}H-NMR, ^{13}C-NMR and HRMS spectrums of 40 .... S50
Figure S20. ^{1}H-NMR, ^{13}C-NMR and HRMS spectrums of 41 .... S53
Figure S21. ^{1}H-NMR, ^{13}C-NMR and HRMS spectrums of 42 .... S56
Figure S22. ^{1}H-NMR, ^{13}C-NMR and HRMS spectrums of 43 .... S59
Figure S23. ^{1}H-NMR, ^{13}C-NMR and HRMS spectrums of 44 .... S62
Figure S24. ^{1}H-NMR, ^{13}C-NMR and HRMS spectrums of 45 .... S65
Figure S25. ^{1}H-NMR, ^{13}C-NMR and HRMS spectrums of 46 .... S68
Figure S26. ^{1}H-NMR, ^{13}C-NMR and HRMS spectrums of 48 .... S71
Figure S27. ^{1}H-NMR, ^{13}C-NMR and HRMS spectrums of 49 .... S74
Figure S28. ^{1}H-NMR, ^{13}C-NMR and HRMS spectrums of 50 .... S77
Figure S29. ^{1}H-NMR, ^{13}C-NMR and HRMS spectrums of 55 .... S80
References S82

Synthesis of Intermediate Compounds

Methyl 4-oxo-3-phenyl-2-thioxo-1,2,3,4-tetrahydroquinazoline-7-carboxylate (7)

To a mixture of dimethylaminoterephthalate (4.78 mmol, 1 eq) in pyridine (8 mL), phenyl isothiocyanate (5.28 mmol, 1.1 eq) was added and stirred for 8 hours at 100 °C. Pyridine was evaporated and reaction mixture was diluted with water. The solid was filtered off under vacuum and dried. The crude product was washed with ethyl acetate. Yield 66%; mp >300 °C (decomp). ^{1}H NMR (400 MHz, DMSO-d$_6$): δ_H 3.92 (3H, s), 7.29 (2H, d, *J* = 7.8 Hz), 7.39-7.50 (3H, m), 7.82 (1H, d, *J* = 7.8 Hz), 8.02-8.07 (2H, m), 13.18 (1H, s); HRMS *m/z* calculated for $C_{16}H_{13}N_2O_3S$ [M+H]$^+$ 313.0647, found: 313.0633. CAS #514857-29-5.

Methyl 2-((2-chlorobenzyl)thio)-4-oxo-3-phenyl-3,4-dihydroquinazoline-7-carboxylate (8)

To a solution of Compound 7 (1.2 mmol, 1 eq) in DMF (2 mL), Cs_2CO_3 (3 mmol, 2.5 eq) and 2-chlorobenzyl bromide (1.2 mmol, 1 eq) were added and the reaction mixture was stirred at room temperature for 3 hours. The mixture was diluted with water and acidified to pH 5 with 2N HCl. The solid was filtered under vacuum. The crude product was used in the next step. Yield 94%; mp 180.5-181.9 °C. ^{1}H NMR (400 MHz, DMSO-d$_6$): δ_H 3.95 (3H, s), 4.55 (2H, s), 7.28-7.32 (2H, m), 7.43-7.48 (3H, m), 7.55-7.57 (3H, m), 7.66-7.69 (1H, m), 7.97 (1H, dd, *J* = 8.4, 1.6 Hz), 8.19-8.22 (2H, m); HRMS *m/z* calculated for $C_{23}H_{18}N_2O_3SCl$ [M+H]$^+$ 437.0714, found: 437.0727.

Methyl 4-oxo-3-phenyl-2-((4-(trifluoromethyl)benzyl)thio)-3,4-dihydroquinazoline-7-carboxylate (9)

Prepared from Compound 7 and 4-(trifluoromethyl)benzyl bromide under the same conditions that applied to Compound 8. Yield 81%; mp 174.9-176.7 °C. ^{1}H NMR (400 MHz, DMSO-d$_6$): δ_H 3.95 (3H, s), 5.52 (2H, s), 7.46-7.49 (2H, m), 7.55-7.58 (3H, m), 7.65-7.70 (4H, m), 7.97 (1H, dd, *J* = 8.4, 1.6 Hz), 8.18-8.20 (2H, m); HRMS *m/z* calculated for $C_{24}H_{18}N_2O_3SF_3$ [M+H]$^+$ 471.0984, found: 471.0990.

2-Aminoterephthalic acid (15)

Dimethylaminoterephthalate (9.56 mmol, 1 eq) and LiOH.H$_2$O (23.92 mmol, 2.5 eq) were dissolved in THF:H$_2$O (3:3 mL) and stirred under reflux for 2 hours. THF was evaporated, the reaction mixture diluted with water and the pH was adjusted to 5 with HCl. The solid was filtered under vacuum and dried. The crude product was used in the next step. Yield 95%; mp

323.0°C (decomp). HRMS m/z calculated for $C_8H_8NO_4$ $[M+H]^+$ 182.0453, found 182.0448. CAS #10312-55-7.

2-amino-4-(methoxycarbonyl)benzoic acid (16)

To a solution of Compound 15 (9 mmol, 1 eq) in MeOH (4 mL), chloromethylsilane (13.5 mmol, 1.5 eq) was added and refluxed for 4 hours. After cooling at rt, the reaction mixture was concentrated and saturated aqueous solution of K_2CO_3 were added, and the solution was extracted with ethyl acetate. The aqueous layer was acidified at pH 5 with acetic acid and extracted with ethyl acetate. The organic layer was dried, filtered, and evaporated. The crude was used in the next step. Yield 80%; mp 218.2-220.2°C. HRMS m/z calculated for $C_9H_{10}NO_4$ $[M+H]^+$ 196.0610, found 196.0617. CAS #85743-02-8.

Methyl 4-oxo-2-thioxo-1,2,3,4-tetrahydroquinazoline-7-carboxylate (17)

Step 1: Compound 16 (5.584 mmol, 1 eq) in $SOCl_2$ (3.5 mL) was refluxed for 3 hours. The reaction mixture was then concentrated in vacuo. The obtained methyl-3-amino-4-(chlorocarbonyl) benzoate was used in the next step. *Step 2:* Methyl-3-amino-4-(chlorocarbonyl) benzoate (5.584 mmol, 1 eq) was dissolved in acetone (4 mL) and added dropwise to a suspension of NH_4SCN (437.8 mg, 5.752 mmol, 1.03 eq) in acetone (2mL). The reaction mixture was stirred at rt for 2 hours and filtered off under vacuum. This solid was then suspended in an aqueous solution of NaOH (10% w/w, 5 mL), stirred and filtered off under vacuum. Water was added to the residue and mixture was acidified to pH 2 with aqueous 2N HCl, the solid was filtered under vacuum. The compound was used in the next step. Yield 60%; mp 269.2-271.2°C. ^1H NMR (400 MHz, DMSO-d_6): δ_H 3.90 (3H, s), 7.78 (1H, dd, J = 8.0, 1.6 Hz), 7.92 (1H, d, J = 1.6 Hz), 8.02 (1H, d, J = 8.0 Hz), 12.62 (1H, s), 12.84 (1H, s); HRMS *m/z* calculated for $C_{10}H_9N_2O_3S$ $[M+H]^+$ 237.0334, found: 237.0339. CAS #422277-15-4.

4-oxo-2-thioxo-1,2,3,4-tetrahydroquinazoline-7-carboxylic acid (18)

Prepared from Compound 17 under the same conditions that used for Compound 10. Yield 80%; mp 264.8 °C (decomp.). ^1H NMR (400 MHz, DMSO-d_6): δ_H 7.77 (1H, d, J = 8.4 Hz), 7.92 (1H, s), 8.02 (1H, d, J = 8.4 Hz), 12.59 (1H, s), 12.84 (1H, s); HRMS *m/z* calculated for $C_9H_7N_2O_3S$ $[M+H]^+$ 223.0177, found: 223.0172. CAS #422277-16-5.

N-neopentyl-4-oxo-2-thioxo-1,2,3,4-tetrahydroquinazoline-7-carboxamide (19)

Prepared from Compound 18 under the same condition that was used in Method A. Yield 30%; mp 198.5-200.4 °C (decomp.). ^1H NMR (400 MHz, DMSO-d_6): δ_H 0.91 (9H, s), 3.10 (2H, d, J = 6.4 Hz), 7.67 (1H, dd, J = 8.0, 1.2 Hz), 7.74 (1H, d, J = 1.2 Hz), 7.98 (1H, d, J = 8.0 Hz),

8.58 (1H, d, *J* = 6.4 Hz), 12.54 (1H, s), 12.80 (1H, s); HRMS *m/z* calculated for $C_{14}H_{18}N_3O_2S$ $[M+H]^+$ 292.1120, found: 292.1132.

Method B: General synthesis method for the alkylation of quinazolinone-7-carboxylic acids

Compound **17** (1.5 mmol, 1 eq) was dissolved in EtOH (3 mL) and 1N NaOH (1.5 mL), appropriate benzyl bromide derivate (1.5 mmol, 1 eq) was added dropwise and refluxed for 2 hours. The mixture was diluted with water and acidified with 2N HCl to pH 3. The solid was filtered under vacuum. The compounds were used in the next step.

2-((2-Chlorobenzyl)thio)-4-oxo-3,4-dihydroquinazoline-7-carboxylic acid (22)

It was prepared according to Method B. Yield 85%; mp 280°C (decomp). ^{1}H NMR (500 MHz, DMSO-*d₆*): δ_H 4.62 (2H, s), 7.31-7.34 (2H, m), 7.48-7.52 (1H, m), 7.70-7.74 (1H, m), 7.91 (1H, dd, *J* = 8.3, 1.4 Hz), 8.11-8.13 (2H, m), 12.79 (1H, s), 13.47 (1H, s). ^{13}C-NMR (125 MHz, DMSO-d_6): δ_C 32.10, 123.44, 125.99, 127.14, 127.58, 127.85, 129.89, 129.93, 132.14, 133.88, 135.22, 136.76, 148.59, 156.46, 161.18, 166.98. HRMS (m/z) $[M+H]^+$ calcd for $C_{16}H_{12}ClN_2O_3S$ $[M+H]^+$ 347.0257, found 347.0259.

2-((3-chlorobenzyl)thio)-4-oxo-3,4-dihydroquinazoline-7-carboxylic acid (23)

Prepared from Compound 17 and 3-chlorobenzyl bromide under the same condition that was used in Method B. Yield 71%; mp > 300.0 °C (decomp.). ^{1}H NMR (400 MHz, DMSO-d_6): δ_H 4.51 (2H, s), 7.30-7.42 (2H, m), 7.47 (1H, d, *J* = 7.6 Hz) 7.60 (1H, m), 7.90 (1H, dd, *J* =8.0, 1.2 Hz), 8.08 (1H, d, *J* = 1.2 Hz), 8.12 (1H, d, *J* = 8.0 Hz), 12.90 (1H, s), 13.40 (1H, bs); HRMS *m/z* calculated for $C_{16}H_{12}N_2O_3SCl$ $[M+H]^+$ 347.0251, found: 347.0257.

2-((4-chlorobenzyl)thio)-4-oxo-3,4-dihydroquinazoline-7-carboxylic acid (24)

Prepared from Compound 17 and 4-chlorobenzyl bromide under the same condition that was used in Method B. Yield 91%; mp 310.0 °C (decomp.). ^{1}H NMR (400 MHz, DMSO-d_6): δ_H 4.50 (2H, s), 7.38 (2H, d, *J* = 8.4 Hz), 7.52 (2H, d, *J* = 8.4 Hz), 7.90 (1H, dd, *J* = 8.0, 1.6 Hz), 8.07 (1H, d, *J* = 1.6 Hz), 8.11 (1H, d, *J* = 8.0 Hz), 12.78 (1H, s); HRMS *m/z* calculated for $C_{16}H_{12}N_2O_3SCl$ $[M+H]^+$ 347.0257, found: 347.0248.

4-oxo-2-((2-(trifluoromethyl)benzyl)thio)-3,4-dihydroquinazoline-7-carboxylic acid (25)

Prepared from Compound 17 and 2-(trifluoromethyl)benzyl bromide under the same condition that was used in Method B. Yield 71%; mp 329.1 °C (decomp.). ^{1}H NMR (400 MHz, DMSO-d_6): δ_H 4.72 (2H, s), 7.52 (1H, t, *J* = 7.8 Hz), 7.66 (1H, t, *J* = 7.8 Hz), 7.76 (1H, d, *J* = 7.8 Hz), 7.85 (1H, d, *J* = 7.8 Hz), 7.91 (1H, dd, *J* = 8.0, 1.4 Hz), 8.07 (1H, d, *J* = 1.4 Hz), 8.13 (1H, d, *J*

= 8.0 Hz), 12.83 (1H, s); HRMS *m/z* calculated for $C_{17}H_{12}N_2O_3SF_3$ $[M+H]^+$ 381.0521, found: 381.0506.

2-((3,4-dichlorobenzyl)thio)-4-oxo-3,4-dihydroquinazoline-7-carboxylic acid (26)

Prepared from Compound 17 and 3,4-dichlorobenzyl chloride under the same condition that was used in Method B. Yield 75%; mp 258.1 °C (decomp.). ^{1}H NMR (400 MHz, DMSO-d$_6$): δ_H 4.50 (2H, s), 7.51 (1H, dd, *J* = 8.4, 2.0 Hz), 7.57 (1H, d, *J* = 8.4 Hz), 7.81 (1H, d, *J* = 2.0 Hz), 7.90 (1H, dd, *J* = 8.4, 1.4 Hz), 8.09 (1H, d, *J* = 1.4 Hz), 8.11 (1H, d, *J* = 8.4 Hz), 12.79 (1H, s); HRMS *m/z* calculated for $C_{16}H_{11}N_2O_3SCl_2$ $[M+H]^+$ 380.9667, found: 380.9662.

2-((2-fluorobenzyl)thio)-4-oxo-3,4-dihydroquinazoline-7-carboxylic acid (27)

Prepared from Compound 17 and 2-fluorobenzyl bromide under the same condition that was used in Method B. Yield 74%; mp 210.0 °C (decomp.). ^{1}H NMR (400 MHz, DMSO-d$_6$): δ_H 4.53 (2H, s), 7.12-7.36 (4H, m), 7.60 (1H, td, *J* = 7.7, 1.8 Hz), 7.88 (1H, dd, *J* = 8.2, 1.6 Hz), 8.07 (1H, d, *J* = 1.6 Hz), 8.09 (1H, d, *J* = 8.2 Hz), 12.76 (1H, s); HRMS *m/z* calculated for $C_{16}H_{12}N_2O_3SF$ $[M+H]^+$ 335.0553, found: 335.0538.

2-((2-cyanobenzyl)thio)-4-oxo-3,4-dihydroquinazoline-7-carboxylic acid (28)

Prepared from Compound 17 and 2-cyanobenzyl bromide under the same condition that was used in Method B. Yield 87%; mp 257.0 °C (decomp). HRMS *m/z* calculated for $C_{17}H_{12}N_3O_3S$ $[M+H]^+$ 338.0599, found: 338.0607.

2-((2-methylbenzyl)thio)-4-oxo-3,4-dihydroquinazoline-7-carboxylic acid (29)

Prepared from Compound 17 and 2-methylbenzyl bromide under the same condition that was used in Method B. Yield 84%; mp 252.5 °C (decomp). HRMS *m/z* calculated for $C_{17}H_{15}N_2O_3S$ $[M+H]^+$ 327.0803, found: 327.0798.

2-((2-methoxybenzyl)thio)-4-oxo-3,4-dihydroquinazoline-7-carboxylic acid (30)

Prepared from Compound 17 and 2-methoxybenzyl chloride under the same condition that was used in Method B. Yield 61%; mp 230.0 °C (decomp.). ^{1}H NMR (400 MHz, DMSO-d$_6$): δ_H 3.84 (3H, s), 4.54 (2H, s), 7.14-7.24 (2H, m), 7.30-7.35 (1H, m), 7.62 (1H, td, *J* = 7.6, 1.6 Hz), 7.90 (1H, dd, *J* = 8.0, 1.2 Hz), 8.09 (1H, d, *J* = 1.2 Hz), 8.11 (1H, d, *J* = 8.0 Hz), 12.78 (1H, s); HRMS *m/z* calculated for $C_{17}H_{15}N_2O_4S$ $[M+H]^+$ 343.0753, found: 343.0751.

4-oxo-2-((2-(trifluoromethoxy)benzyl)thio)-3,4-dihydroquinazoline-7-carboxylic acid (31)

Prepared from Compound 17 and 2-(trifluoromethoxy)benzyl bromide under the same condition that was used in Method B. Yield 90%; mp 287.0 °C (decomp.). ^{1}H NMR (400 MHz, DMSO-d$_6$): δ_H 4.60 (2H, s), 7.34-7.45 (3H, m), 7.72-7.74 (1H, m), 7.90 (1H, dd, *J* = 8.0, 1.2 Hz),

8.08 (1H, d, *J* = 1.2 Hz), 8.12 (1H, d, *J* = 8.0 Hz), 12.90 (1H, bs); HRMS *m/z* calculated for $C_{17}H_{12}N_2O_4SF_3Cl$ [M+H]$^+$ 397.0470, found: 397.0473.

2-(((2-methylpyridin-3-yl)methyl)thio)-4-oxo-3,4-dihydroquinazoline-7-carboxylic acid (32)

Prepared from Compound 17 and 3-(chloromethyl)-2-methylpyridine under the same condition that was used in Method B. Yield 61%; mp 280.0 °C (decomp.). ^1H NMR (400 MHz, DMSO-d$_6$): δ_H 2.60 (3H, s), 4.55 (2H, s), 7.18 (1H, dd, *J* = 7.8, 1.2 Hz), 7.86-7.91 (2H, m), 8.08 (1H, d, *J* = 1.2 Hz), 8.11 (1H, d, *J* = 8.0 Hz), 8.33 (1H, dd, *J* = 5.2, 1.2 Hz), 12.95 (1H, bs); HRMS *m/z* calculated for $C_{16}H_{12}N_2O_3SF$ [M+H]$^+$ 331.0553, found: 331.0538.

4-oxo-2-((4-(trifluoromethyl)benzyl)thio)-3,4-dihydroquinazoline-7-carboxylic acid (**33**)

Prepared from Compound 17 and 4-(trifluoromethyl)benzyl bromide under the same condition that was used in Method B. Yield 95%; mp 273.0 °C (decomp.). HRMS m/z calculated for $C_{17}H_{12}F_3N_2O_3S$ [M+H]$^+$ 381.0521, found 381.0520.

Tert-butyl 4-(2-((2-chlorobenzyl)thio)-4-oxo-3,4-dihydroquinazoline-7-carbonyl)piperazine-1-carboxylate (47)

Prepared from Compound 22 and *N*-Boc-piperazine under the same condition that was used in Method A. Yield 35%; mp 139.8-141.2 °C. ^1H NMR (400 MHz, DMSO-d$_6$): δ_H 1.41 (9H, s), 3.30 (2H, bs), 3.35 (2H, bs), 3.45 (2H, bs), 3.64 (2H, bs), 4.59 (2H, s), 7.29-7.35 (2H, m), 7.41 (1H, dd, *J* = 8.0, 1.6 Hz), 7.48-7.50 (1H, m), 7.64 (1H, s), 7.73-7.75 (1H, m), 8.07 (1H, d, *J* = 8.0 Hz), 12.73 (1H, s). HRMS (m/z) calcd for $C_{25}H_{28}ClN_4O_4S$ [M+H]$^+$: 515.1520, found: 515.1540.

Methyl 2,4-dioxo-1,2,3,4-tetrahydroquinazoline-7-carboxylate (51)

Compound 16 (1.64 mmol, 1 eq) was taken in acetic acid (5 mL), urea (16.4 mmol, 10 eq) was added, and refluxed for 5 hours. The reaction mixture was taken into water and the solid was filtered off. The crude was used in the next step. Yield 65%; mp > 300.0 °C. HRMS m/z calculated for $C_{10}H_9N_2O_4$ [M+H]$^+$, 221.0562, found 221.0551. CAS # 174074-88-5

Methyl 2,4-dichloroquinazoline-7-carboxylate (52)

Compound 51 (1.07 mmol, 1 eq) was taken into toluene (5mL) and DIEA (3.2 mmol, 3 eq) and $POCl_3$ (3 mL) were added to it and stirred at 90 °C for 3 hours. At the end of the period, the reaction flask was cooled with the help of an ice bath, water was added dropwise and extracted with ethyl acetate. The extract was re-extracted with the prepared $NaHCO_3$ solution. The organic layer was dried, filtered, and evaporated. The crude was used in the next step. Yield 61%; mp 129.0-131.0 °C. HRMS m/z calculated for $C_{10}H_7Cl_2N_2O_2$ [M+H]$^+$, 256.9885, found 256.9876 . CAS #174074-89-6

2-Chloro-4-oxo-3,4-dihydroquinazoline-7-carboxylic acid (53)

Compound 52 (1 mmol, 1 eq) was heated with 1N NaOH solution (4 mL) under reflux for 2 hours. The reaction mixture was diluted with water and acidified with acetic acid, the solid was filtered and dried. The compound was used in the next step Yield 83%; mp 247.0 °C (decomp.). HRMS m/z calculated for $C_9H_6ClN_2O_3$ $[M+H]^+$, 225.0067, found 225.0073. CAS #1594503-33-9

2-((2-Chlorobenzyl)amino)-4-oxo-3,4-dihydroquinazoline-7-carboxylic acid (54)

To the ethanol solution of compound 53 (0.8 mmol, 1 eq), DIEA (1.2 mmol, 1.5 eq) and 2-chlorobenzylamine (0.96 mmol, 1.2 eq) were added and refluxed for 3 hours. At the end of the time, the reaction mixture was cooled, and water was added, the solid was filtered and dried. Yield 79%; mp 288.0 °C (decomp). ^{1}H NMR (500 MHz, DMSO-d_6): δ_H 4.65 (2H, s), 6.94 (1H, bs), 7.28-7.37 (2H, m), 7.47-7.49 (2H, m), 7.06 (1H, d, *J* = 8.0 Hz), 7.75 (1H, s), 7.98 (1H, d, *J* = 8.0 Hz), 11.23 (1H, bs), 13.24 (1H, bs); HRMS m/z calculated for $C_{16}H_{13}ClN_3O_3$ $[M+H]^+$,330.0645, found 330.0645.

Table S1. Physicochemical properties and predicted ADME properties of compounds **5**, **35** and **37**. Given values are calculated with *QikProp.* [1]

Properties	Compound 5	Compound 35	Compound 37	Range for 95% of Drugs or Recommended Values
Molecular Weight (mol_MW)	471.357	415.937	449.49	130.0 – 725.0
Predicted octanol/water partition coefficient (QPlogPo/w)	5.61	4.679	4.938	−2.0 – 6.5
Number of Hydrogen Bond Donors (donorHB)	1	2	2	0.0 – 6.0
Number of Hydrogen Bond Acceptors (accptHB)	6	6	6	2.0 – 20.0
Rotable Bonds (#rotor)	6	6	6	0-15
Predicted Central Nervous System Activity (CNS)	-2	-1	0	−2 (inactive), +2 (active)
Computed dipole moment (dipole)	6.417	4.047	5.639	1.0 – 12.5
Total solvent accessible surface area (SASA)	748.315	757.062	733.08	300.0 – 1000.0
Hydrophobic component of the SASA (FOSA)	58.991	259.869	253.993	0.0 – 750.0
Hydrophilic component of the SASA (FISA)	143.4	124.021	104.306	7.0 – 330.0
π (carbon and attached hydrogen) component of the SASA (PISA)	404.977	266.19	245.477	0.0 – 450.0
Weakly polar component of the SASA (WPSA)	140.948	106.982	129.304	0.0 – 175.0
Total solvent-accessible volume (volume)	1325.18	1310.161	1316.98	500.0 – 2000.0
Predicted apparent Caco-2 cell permeability in nm/sec (QPPCaco)	109.556	660.409	1015.697	<25 poor, >500 great
Predicted brain/blood partition coefficient (QPlogBB)	-1.025	-0.949	-0.588	−3.0 – 1.2

Predicted apparent MDCK cell permeability in nm/sec (QPPMDCK)	341.083	1217.971	2570.323	<25 poor, >500 great
Predicted skin permeability, log Kp (QPlogKp)	-2.16	-2.291	-2.001	−8.0 – −1.0
PM3 calculated ionization potential (negative of HOMO energy) (IP(eV))	9.334	9.118	9.173	7.9 – 10.5
PM3 calculated electron affinity (negative of LUMO energy) (EA(eV))	1.258	1.059	1.124	−0.9 – 1.7
Prediction of binding to human serum albumin (QPlogKhsa)	0.607	0.678	0.714	−1.5 – 1.5
Predicted human oral absorption on 0 to 100% scale (PercentHumanOralAbsorption)	83.337	100	100	>80% is high <25% is poor
Van der Waals surface area of polar atoms and carbonyl carbon atoms (PSA)	85.651	85.344	82.867	7.0 – 200.0

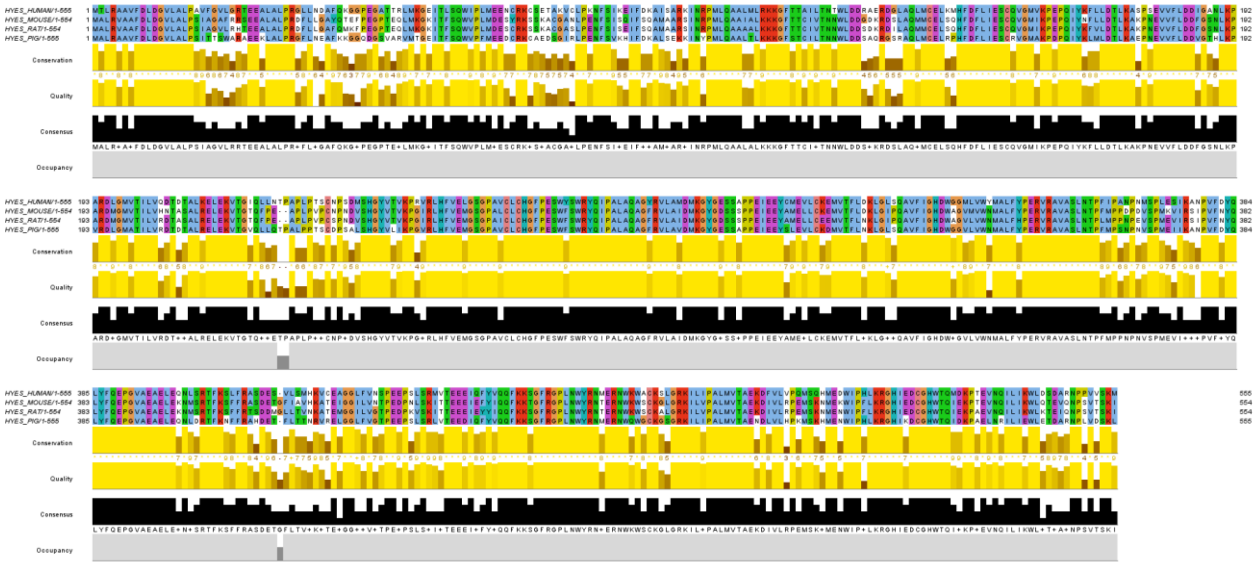

Figure S1. Sequence alignment of soluble epoxide hydrolase amino acid sequences of human (UniProt ID: P34913), mouse (UniProt ID: P34914), rat(UniProt ID: P80299), and pig (UniProt ID: Q6Q2C2) recorded in SwissProt database.[2] Alignment is done with T-Coffee[3] by using JalView.[4]

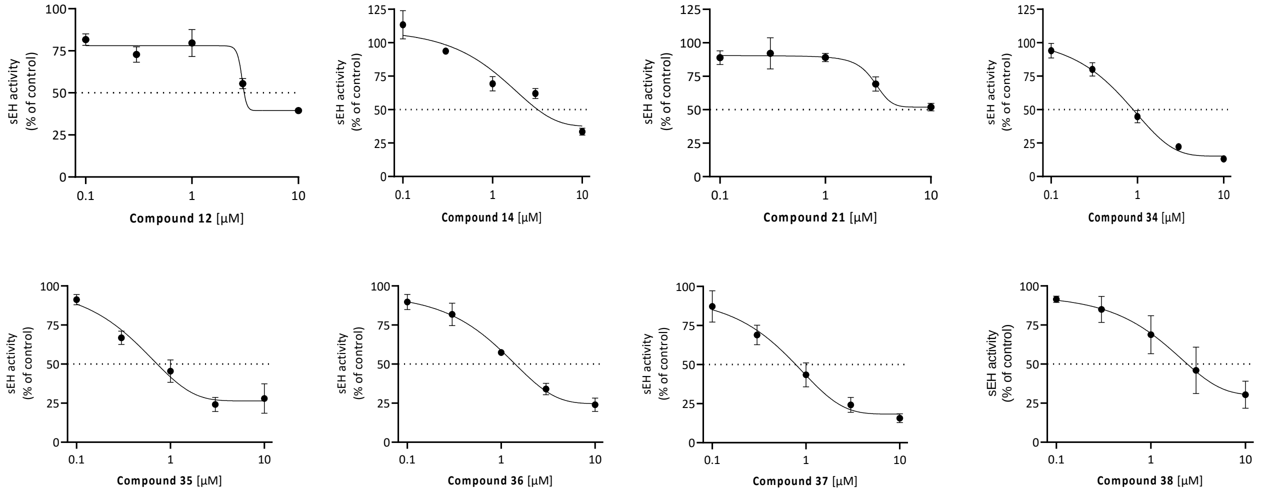

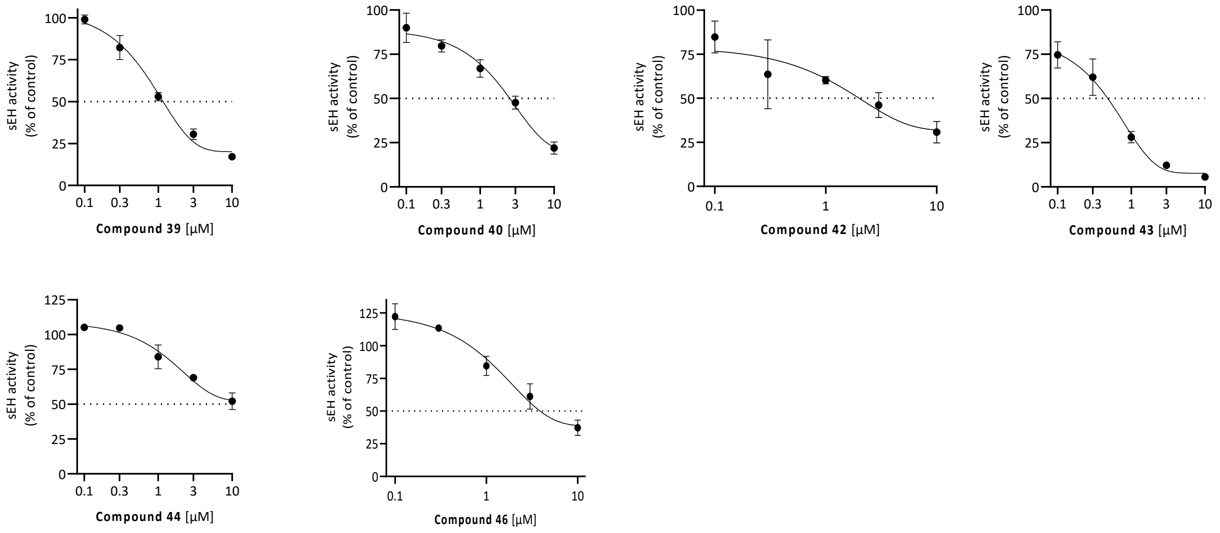

Figure S2. Representative bioactivity results of the selected final compounds.

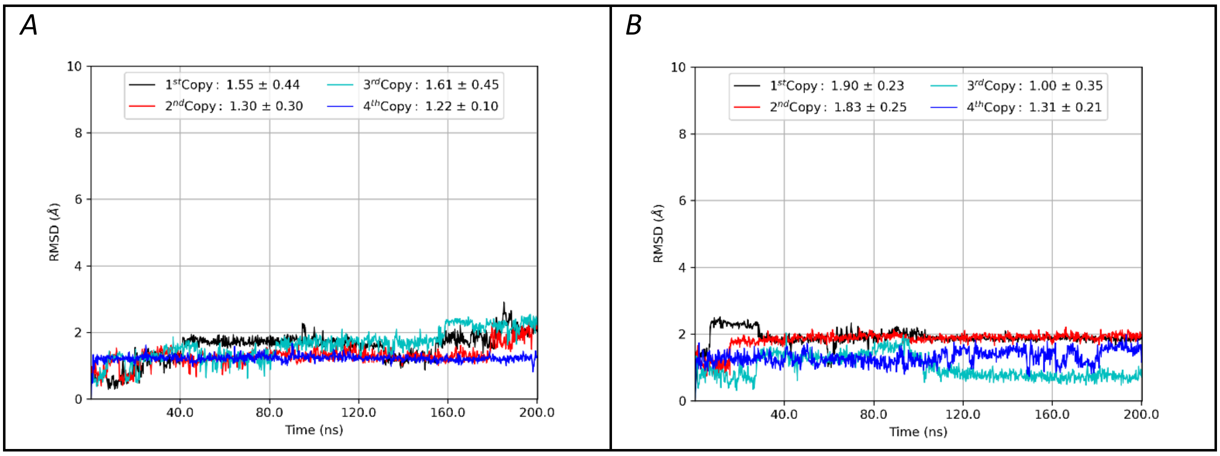

Figure S3. RMSD values of compounds A) **35** and B) **37** simulated with sEH. Both figures were plotted after fitting each frame against the first one.

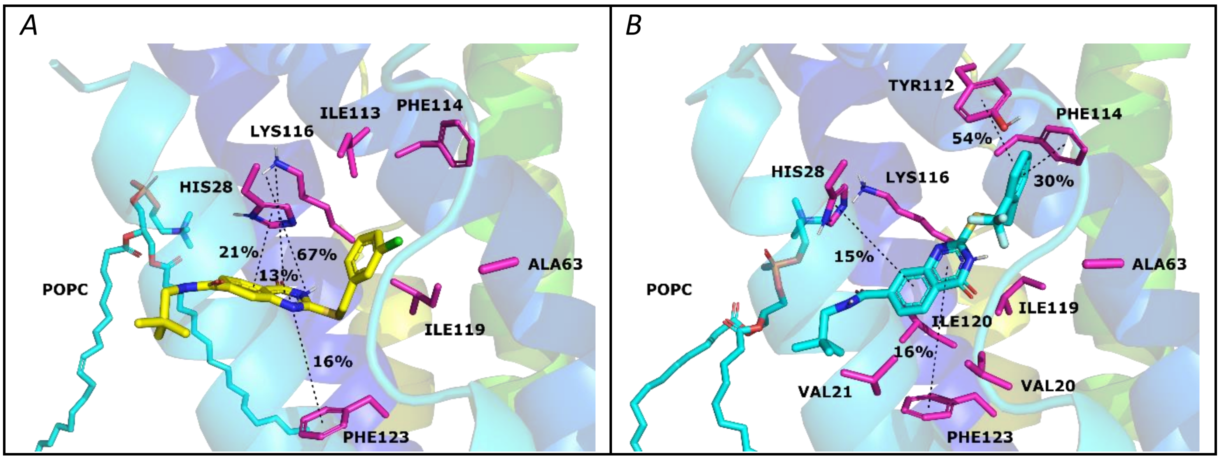

Figure S4. Protein-ligand interactions of A) compound **35** and B) compound **37** at FLAP binding site with their occupancy values calculated during the simulation time of 200 ns.

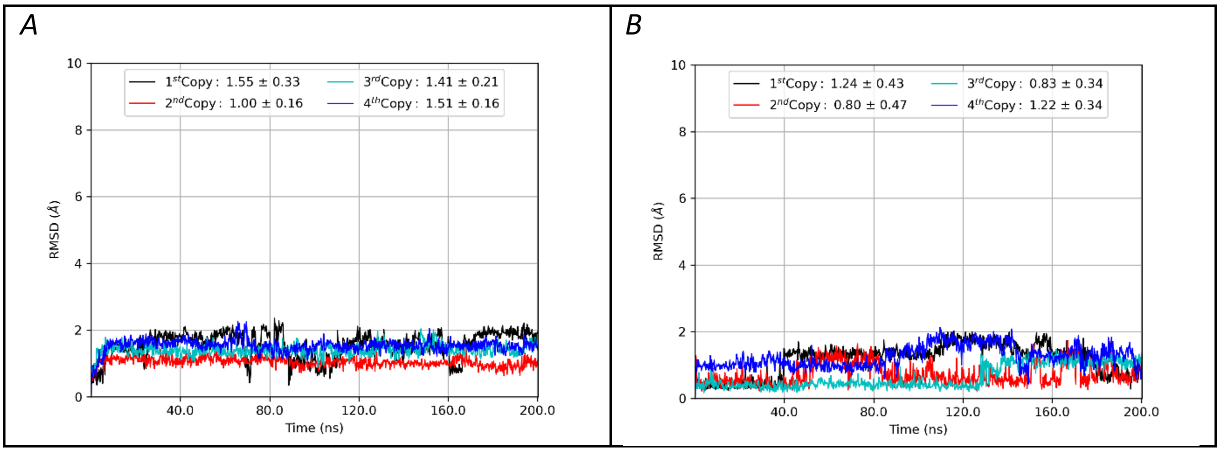

Figure S5. RMSD values of compounds A) **35** and B) **37** simulated with FLAP. Both figures were plotted after fitting each frame against the first one.

Figure S6. ^{1}H-NMR, ^{13}C-NMR and HRMS spectrums of 10

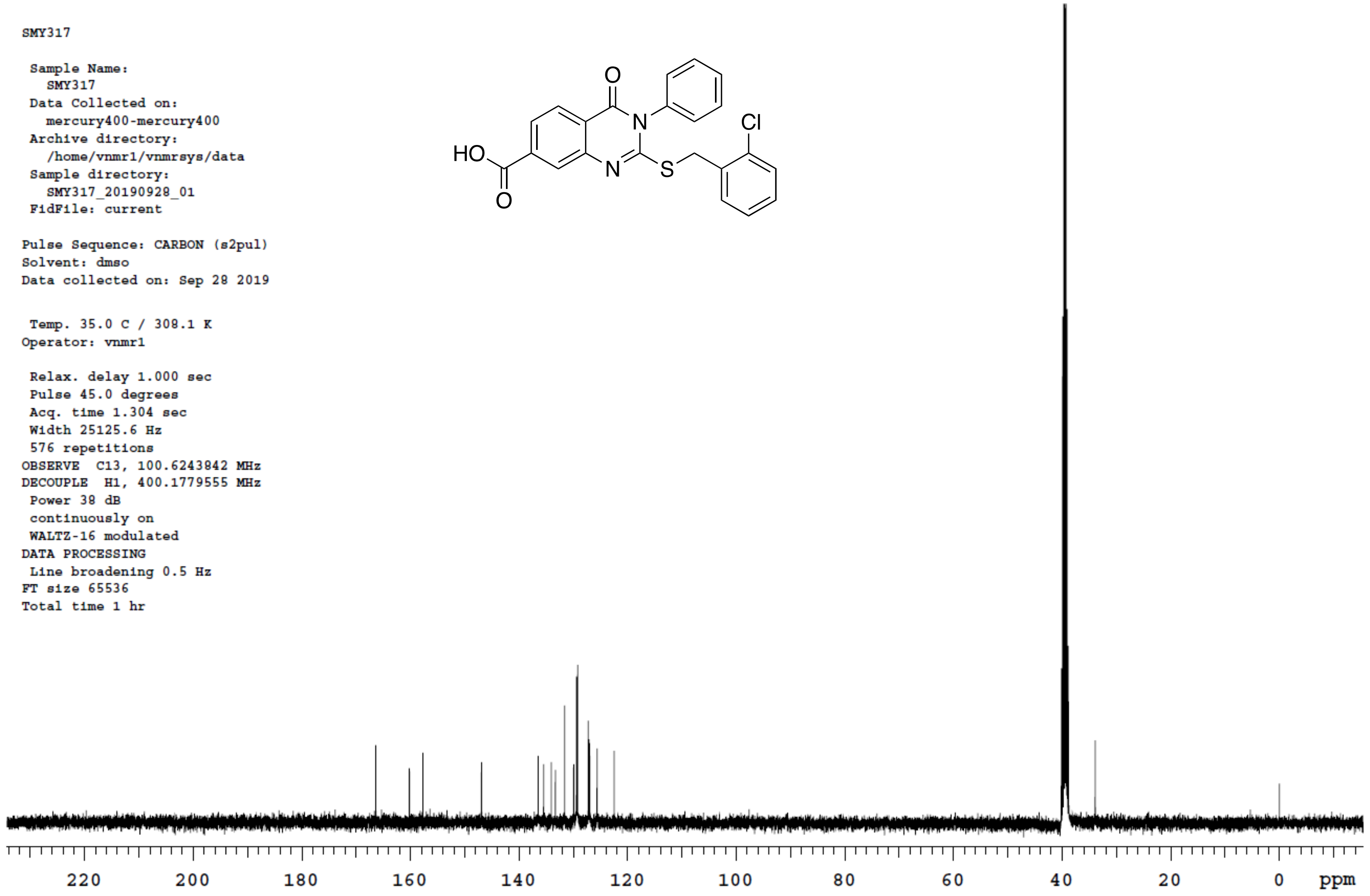

SMY317

Sample Name:
SMY317
Data Collected on:
mercury400-mercury400
Archive directory:
/home/vnmr1/vnmrsys/data
Sample directory:
SMY317_20190928_01
FidFile: PROTON_01

Pulse Sequence: PROTON (s2pul)
Solvent: dmso
Data collected on: Sep 28 2019

Temp. 35.0 C / 308.1 K
Operator: vnmr1

Relax. delay 1.000 sec
Pulse 45.0 degrees
Acq. time 2.559 sec
Width 6402.0 Hz
8 repetitions
OBSERVE H1, 400.1759761 MHz
DATA PROCESSING
FT size 32768
Total time 0 min 31 sec

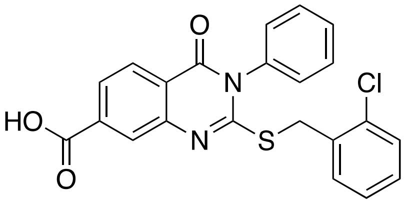

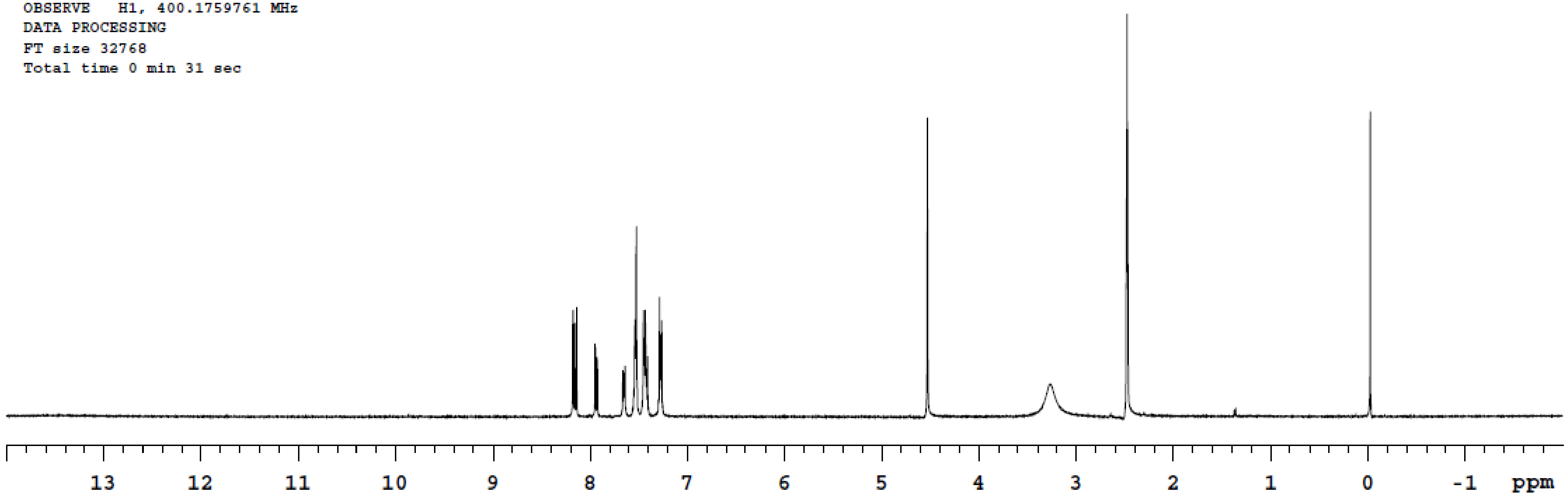

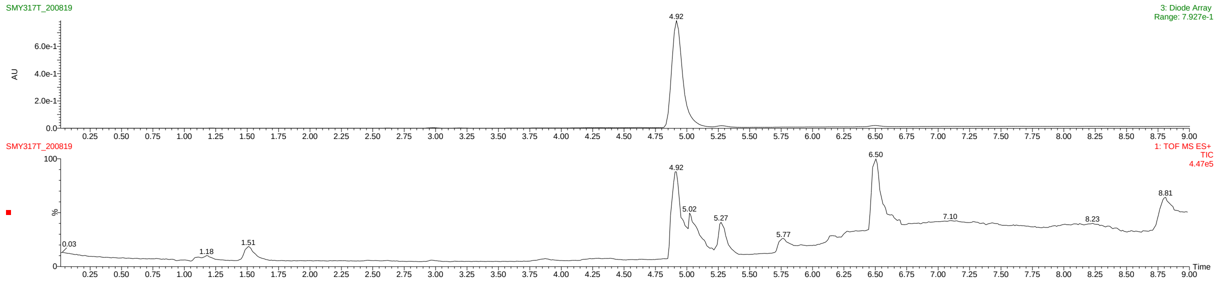

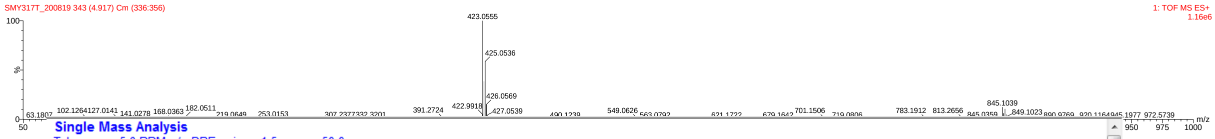

Single Mass Analysis
Tolerance = 5.0 PPM / DBE: min = -1.5, max = 50.0
Element prediction: Off
Number of isotope peaks used for i-FIT = 3
Monoisotopic Mass, Even Electron Ions
63 formula(e) evaluated with 1 results within limits (up to 50 closest results for each mass)
Elements Used:

Mass	Calc. Mass	mDa	PPM	DBE	Formula	i-FIT	i-FIT (Norm)	C	H	N	O	S	Cl
423.0555	423.0570	-1.5	-3.5	15.5	C22 H16 N2 O3 S Cl	788.9	0.0	22	16	2	3	1	1

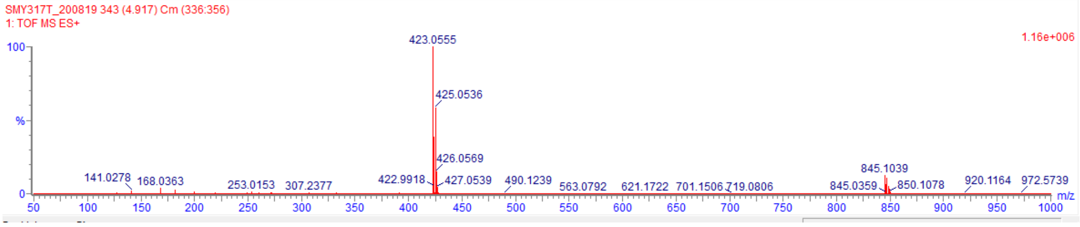

Figure S7. ^{1}H-NMR, ^{13}C-NMR and HRMS spectrums of 11

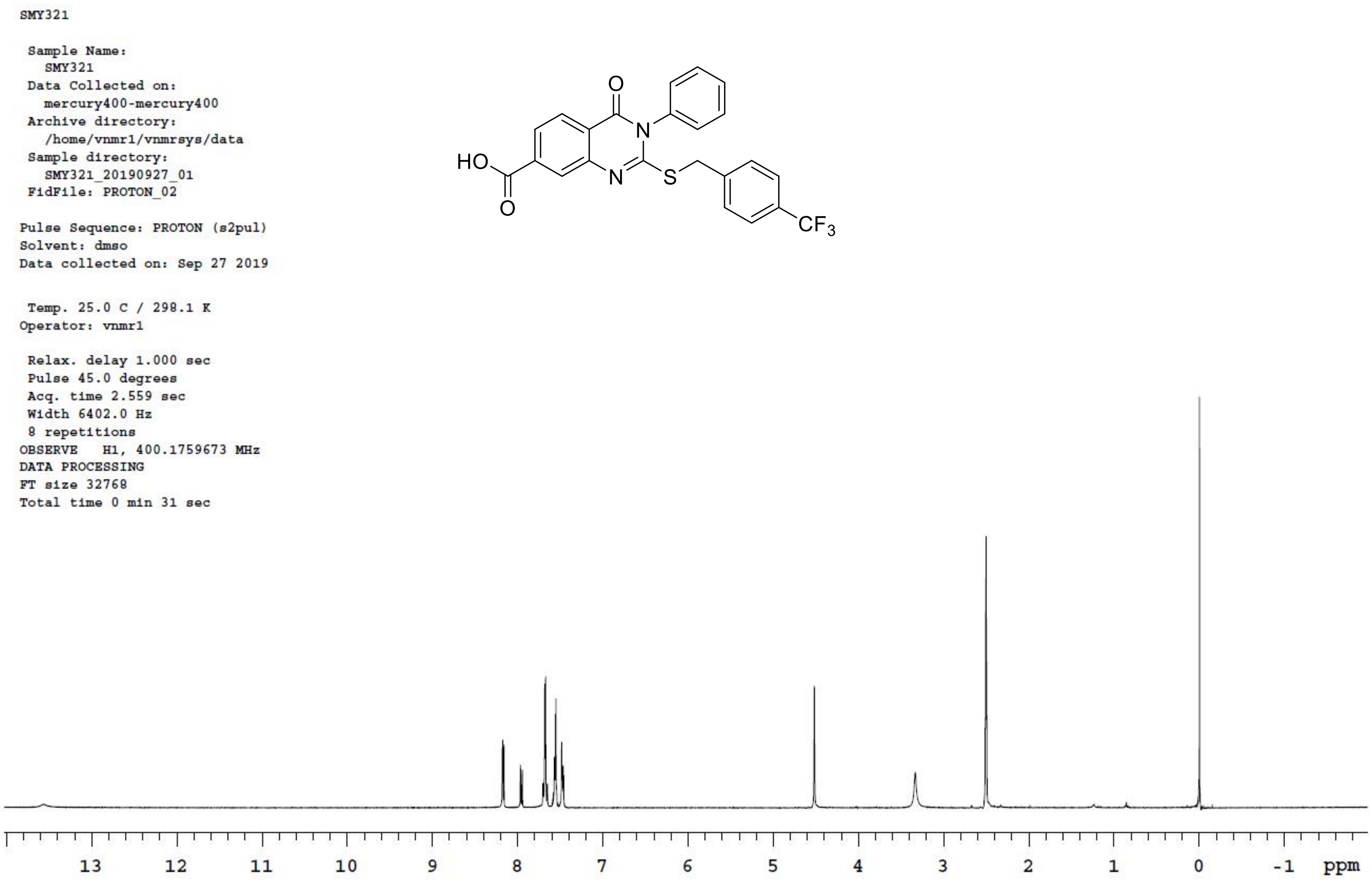

SMY321

Sample Name:
SMY321
Data Collected on:
mercury400-mercury400
Archive directory:
/home/vnmr1/vnmrsys/data
Sample directory:
SMY321_20190927_01
FidFile: CARBON_01

Pulse Sequence: CARBON (s2pul)
Solvent: dmso
Data collected on: Sep 27 2019

Temp. 25.0 C / 298.1 K
Operator: vnmr1

Relax. delay 1.000 sec
Pulse 45.0 degrees
Acq. time 1.304 sec
Width 25125.6 Hz
5000 repetitions
OBSERVE C13, 100.6243774 MHz
DECOUPLE H1, 400.1779555 MHz
Power 38 dB
continuously on
WALTZ-16 modulated
DATA PROCESSING
Line broadening 0.5 Hz
FT size 65536
Total time 3 hr, 19 min

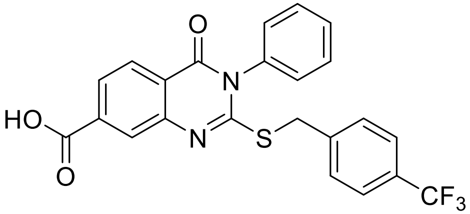

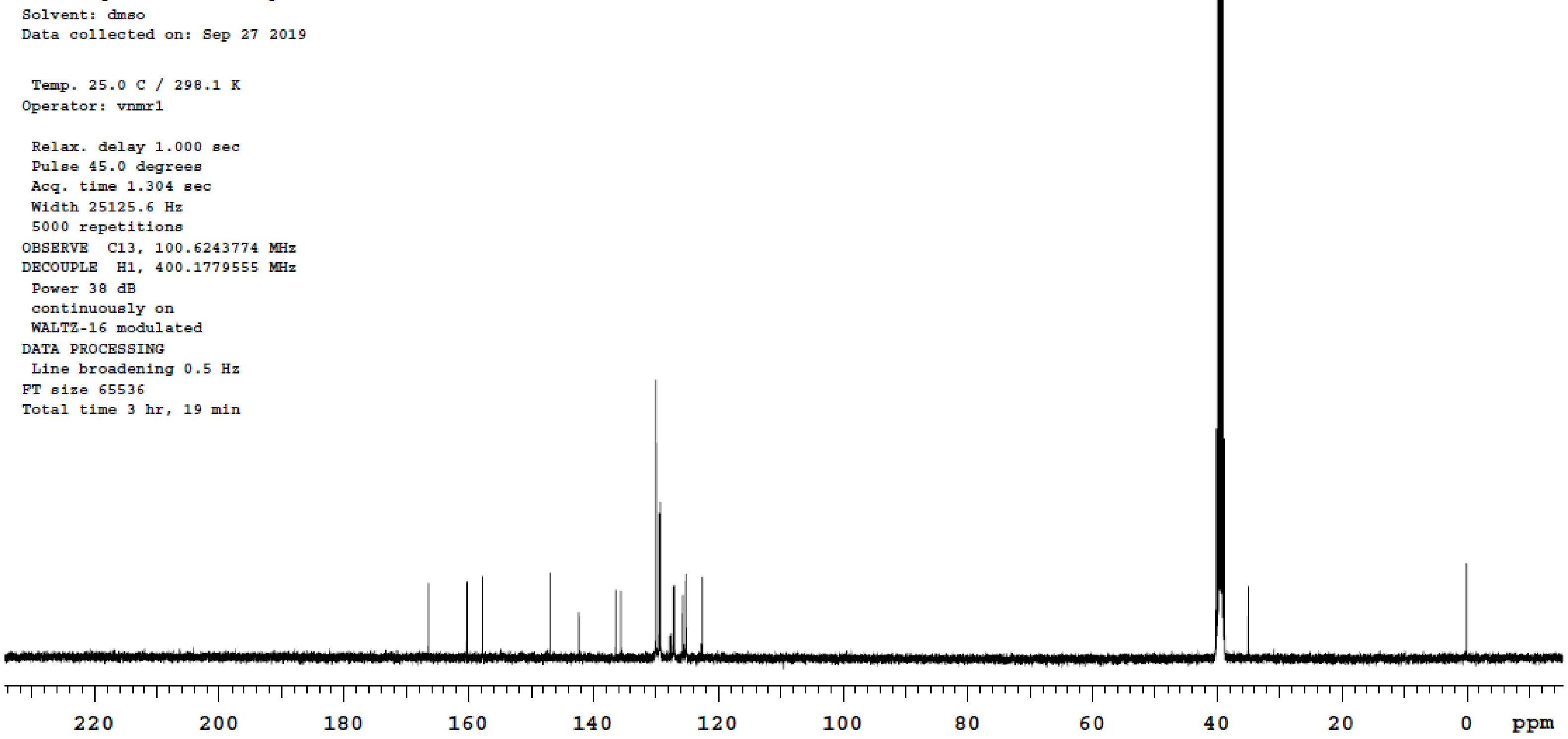

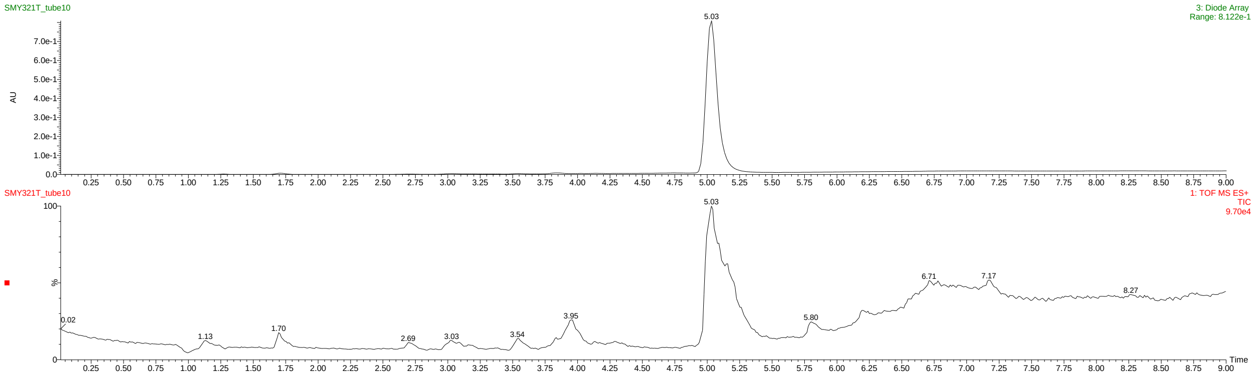

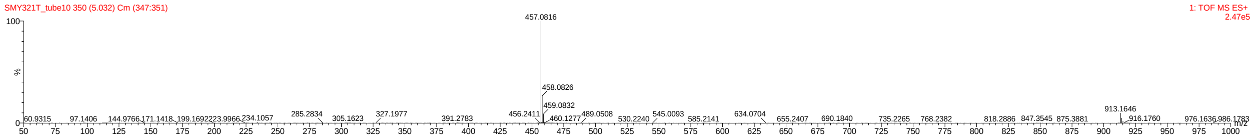

Single Mass Analysis
Tolerance = 5.0 PPM / DBE: min = -1.5, max = 50.0
Element prediction: Off
Number of isotope peaks used for i-FIT = 3
Monoisotopic Mass, Even Electron Ions
59 formula(e) evaluated with 1 results within limits (up to 50 closest results for each mass)
Elements Used:

Mass	Calc. Mass	mDa	PPM	DBE	Formula	i-FIT	i-FIT (Norm)	C	H	N	O	S	F
457.0816	457.0834	-1.8	-3.9	15.5	C23 H16 N2 O3 S F3	292.2	0.0	23	16	2	3	1	3

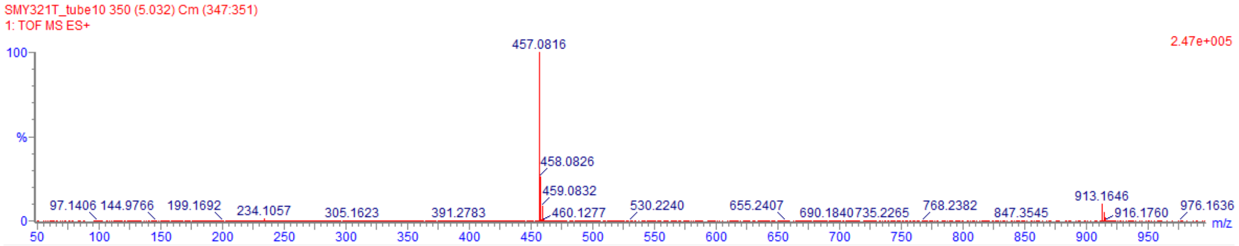

Figure S8. ^{1}H-NMR, ^{13}C-NMR and HRMS spectrums of 12

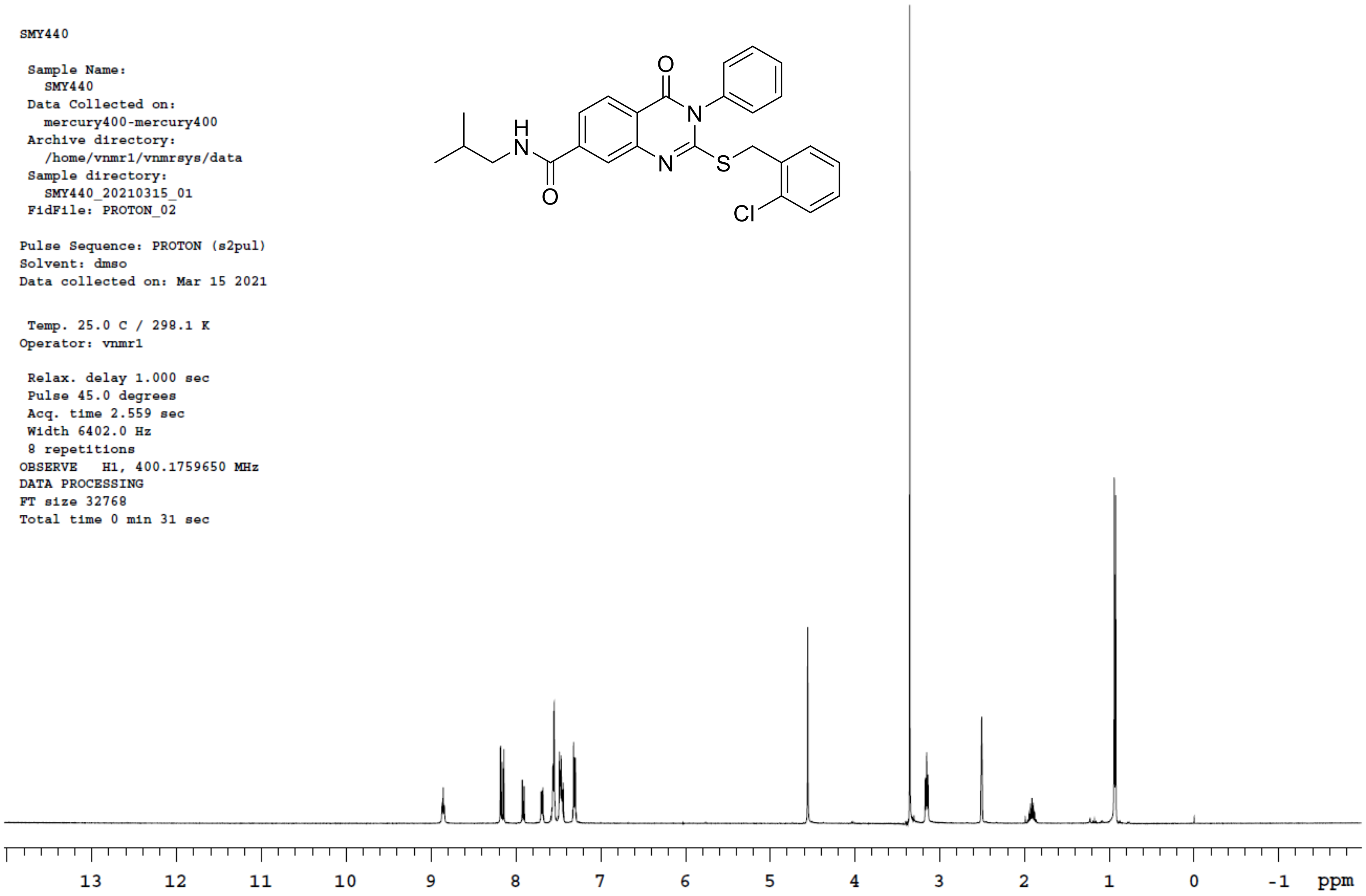

SMY440

Sample Name:
SMY440
Data Collected on:
mercury400-mercury400
Archive directory:
/home/vnmr1/vnmrsys/data
Sample directory:
SMY440_20210315_01
FidFile: CARBON_01

Pulse Sequence: CARBON (s2pul)
Solvent: dmso
Data collected on: Mar 15 2021

Temp. 25.0 C / 298.1 K
Operator: vnmr1

Relax. delay 1.000 sec
Pulse 45.0 degrees
Acq. time 1.550 sec
Width 21141.6 Hz
2000 repetitions
OBSERVE C13, 100.6243742 MHz
DECOUPLE H1, 400.1779555 MHz
Power 38 dB
continuously on
WALTZ-16 modulated
DATA PROCESSING
Line broadening 0.5 Hz
FT size 65536
Total time 1 hr, 28 min

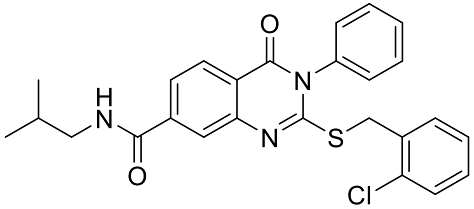

Agilent Technologies

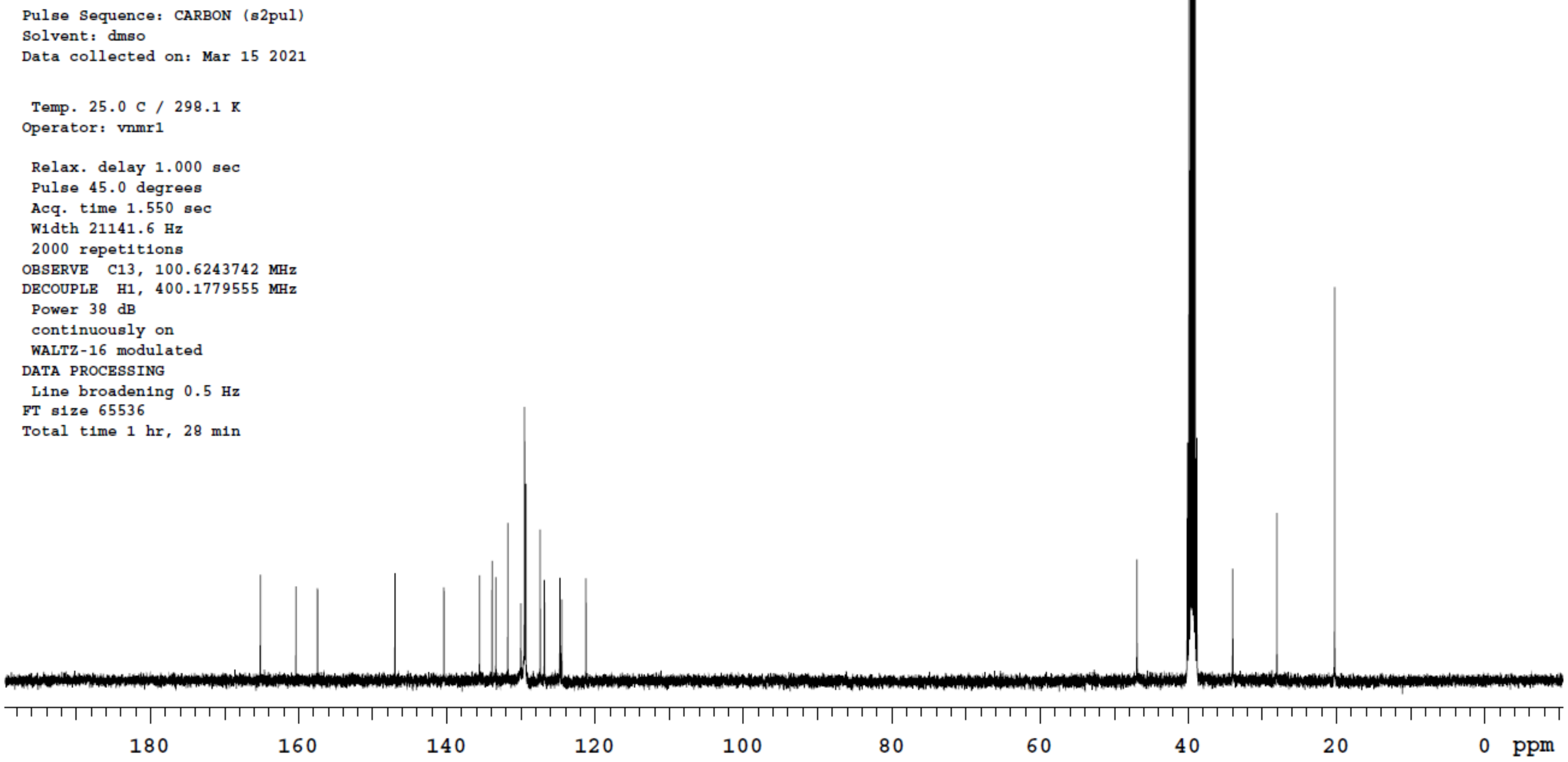

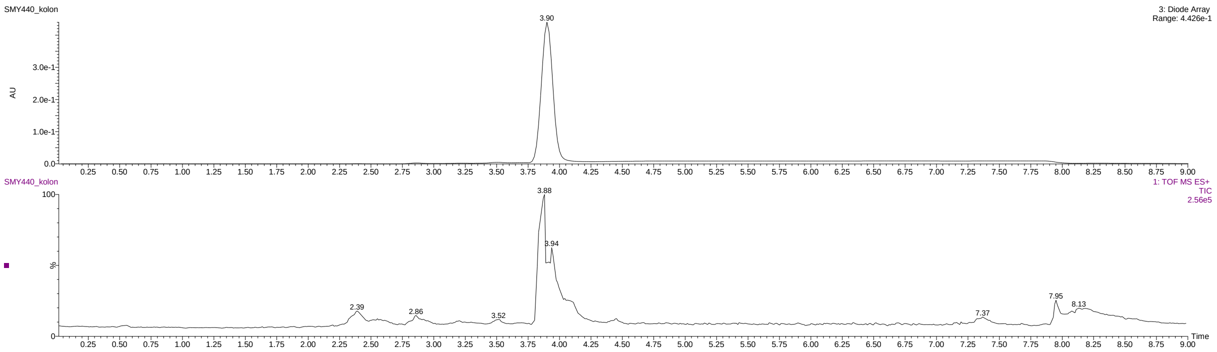

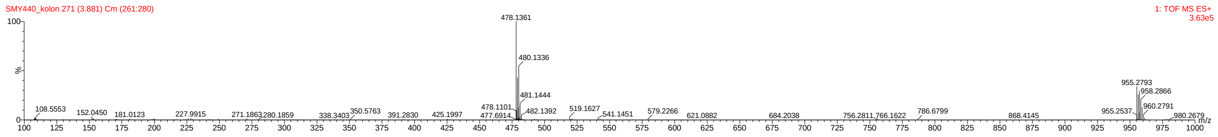

Single Mass Analysis

Tolerance = 5.0 PPM / DBE: min = -1.5, max = 50.0

Element prediction: Off

Number of isotope peaks used for i-FIT = 3

Monoisotopic Mass, Even Electron Ions

79 formula(e) evaluated with 1 results within limits (up to 50 closest results for each mass)

Elements Used:

Mass	Calc. Mass	mDa	PPM	DBE	Formula	i-FIT	i-FIT (Norm)	C	H	N	O	S	Cl
478.1361	478.1356	0.5	1.0	15.5	C26 H25 N3 O2 S Cl	579.8	0.0	26	25	3	2	1	1

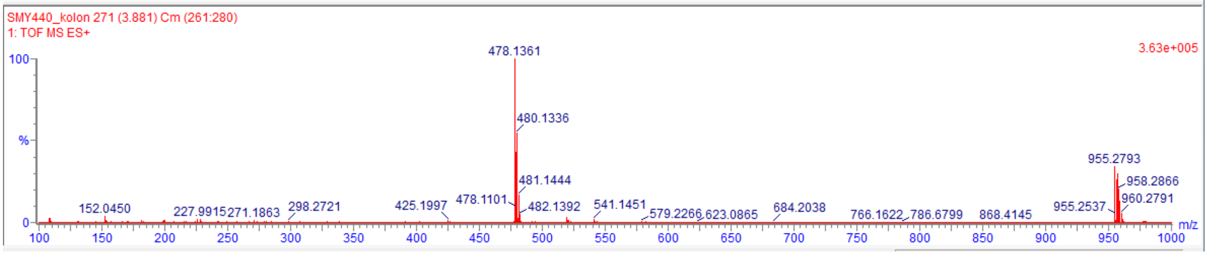

Figure S9. ^{1}H-NMR, ^{13}C-NMR and HRMS spectrums of 13

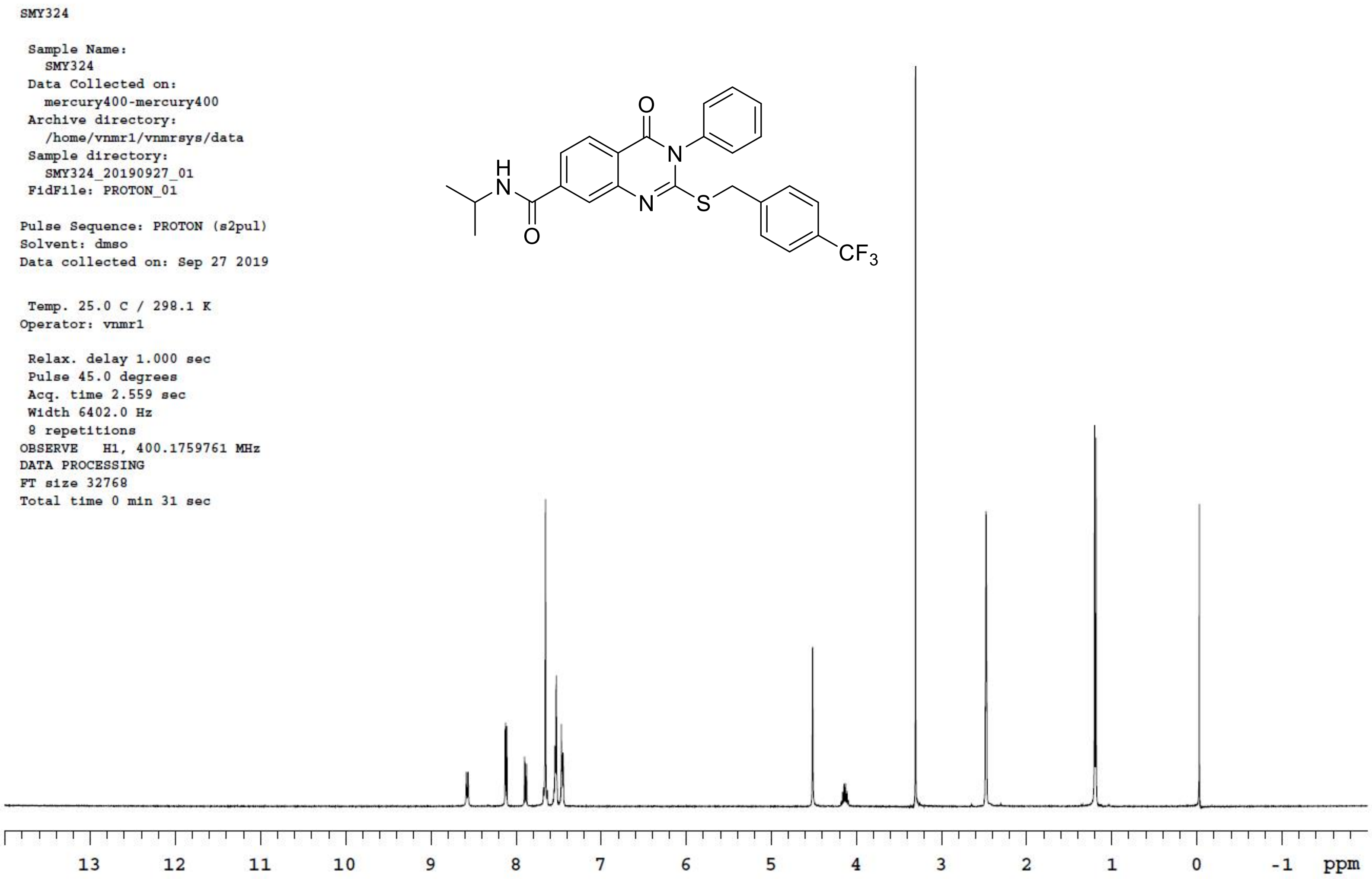

SMY324

Sample Name:
SMY324
Data Collected on:
mercury400-mercury400
Archive directory:
/home/vnmr1/vnmrsys/data
Sample directory:
SMY324_20190927_01
FidFile: current

Pulse Sequence: CARBON (s2pul)
Solvent: dmso
Data collected on: Sep 27 2019

Temp. 25.0 C / 298.1 K
Operator: vnmr1

Relax. delay 1.000 sec
Pulse 45.0 degrees
Acq. time 1.550 sec
Width 21141.6 Hz
64 repetitions
OBSERVE C13, 100.6243836 MHz
DECOUPLE H1, 400.1779555 MHz
Power 38 dB
continuously on
WALTZ-16 modulated
DATA PROCESSING
Line broadening 0.5 Hz
FT size 65536
Total time 3 hr, 40 min

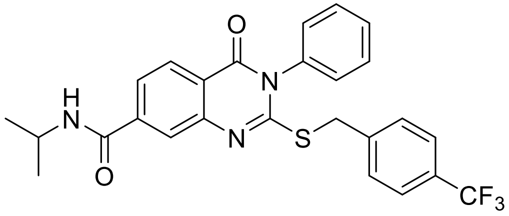

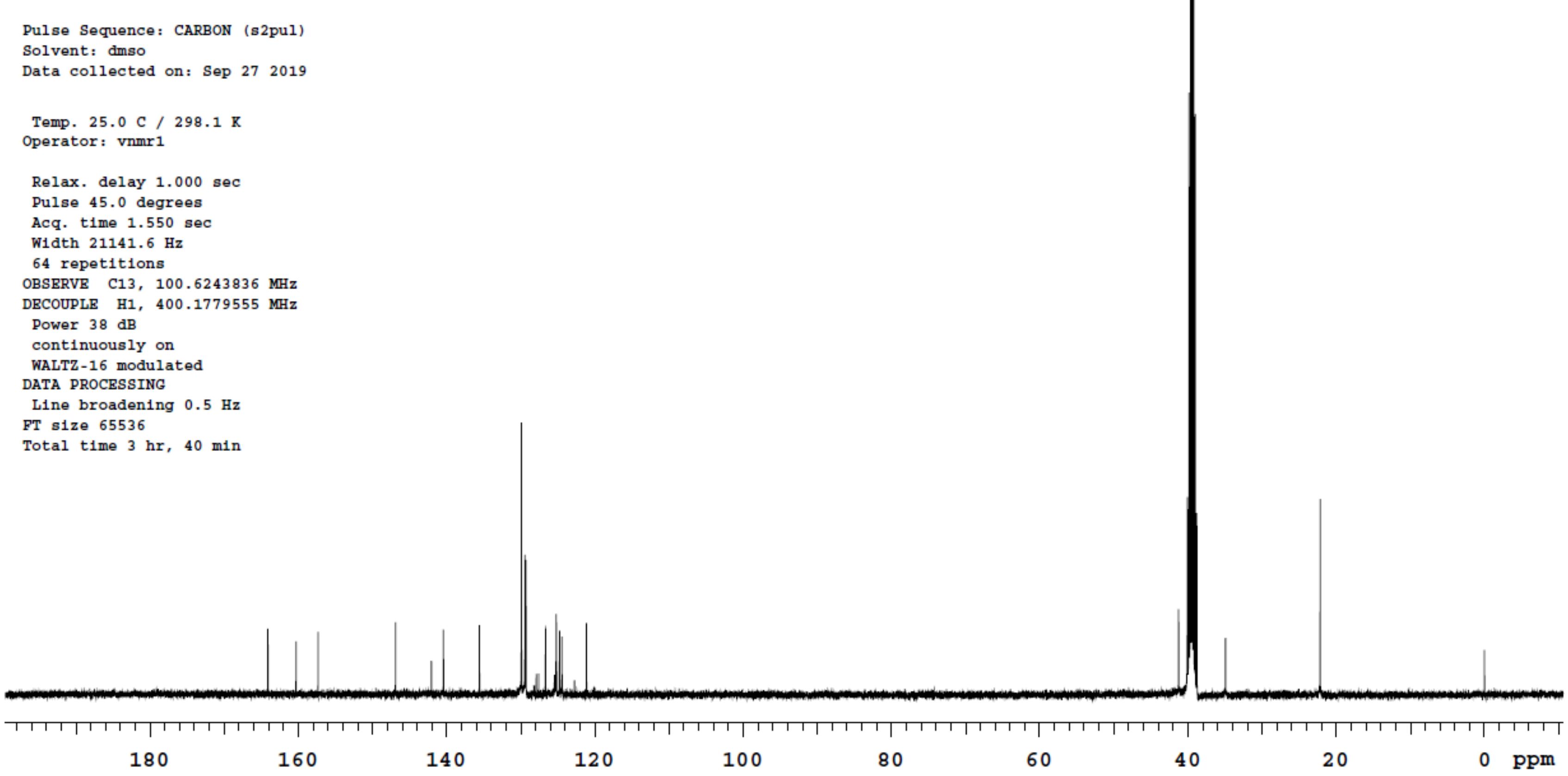

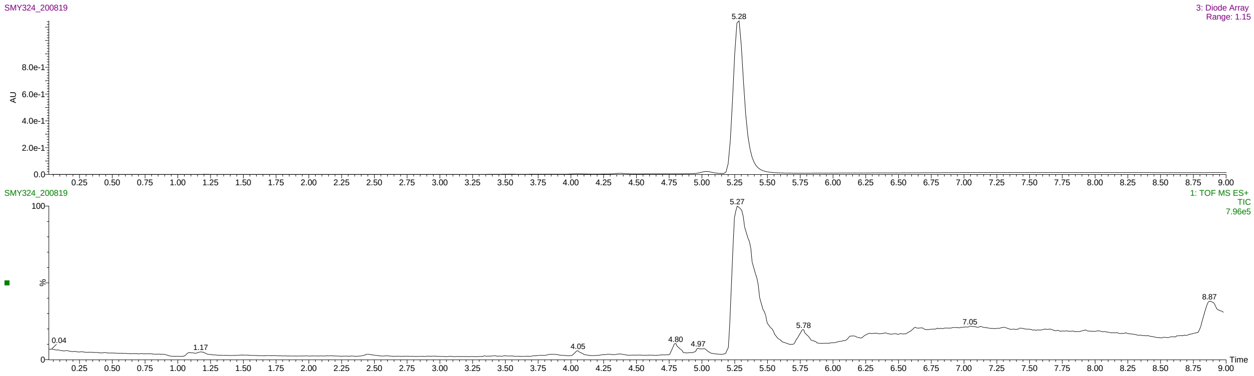

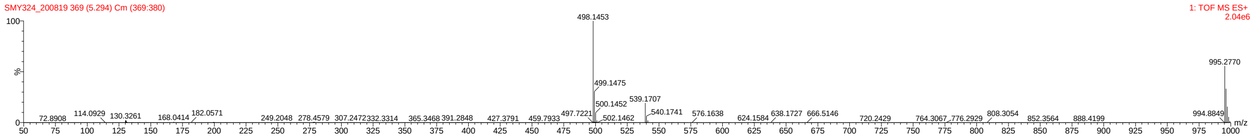

Single Mass Analysis
Tolerance = 5.0 PPM / DBE: min = -1.5, max = 50.0
Element prediction: Off
Number of isotope peaks used for i-FIT = 3
Monoisotopic Mass, Even Electron Ions
67 formula(e) evaluated with 1 results within limits (up to 50 closest results for each mass)
Elements Used:

Mass	Calc. Mass	mDa	PPM	DBE	Formula	i-FIT	i-FIT (Norm)	C	H	N	O	S	F
498.1453	498.1463	-1.0	-2.0	15.5	C26 H23 N3 O2 S F3	690.5	0.0	26	23	3	2	1	3

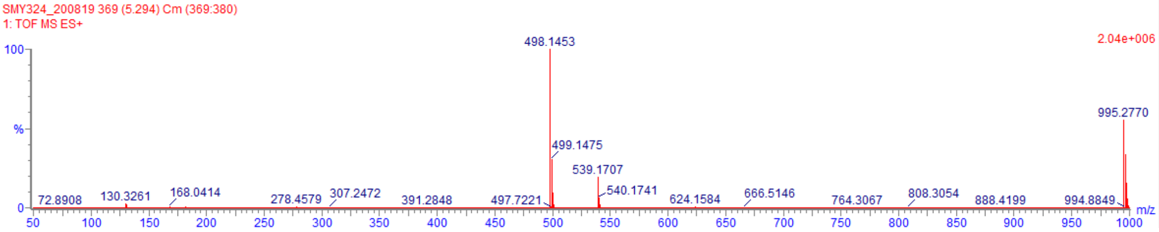

Figure S10. ^{1}H-NMR, ^{13}C-NMR and HRMS spectrums of 14

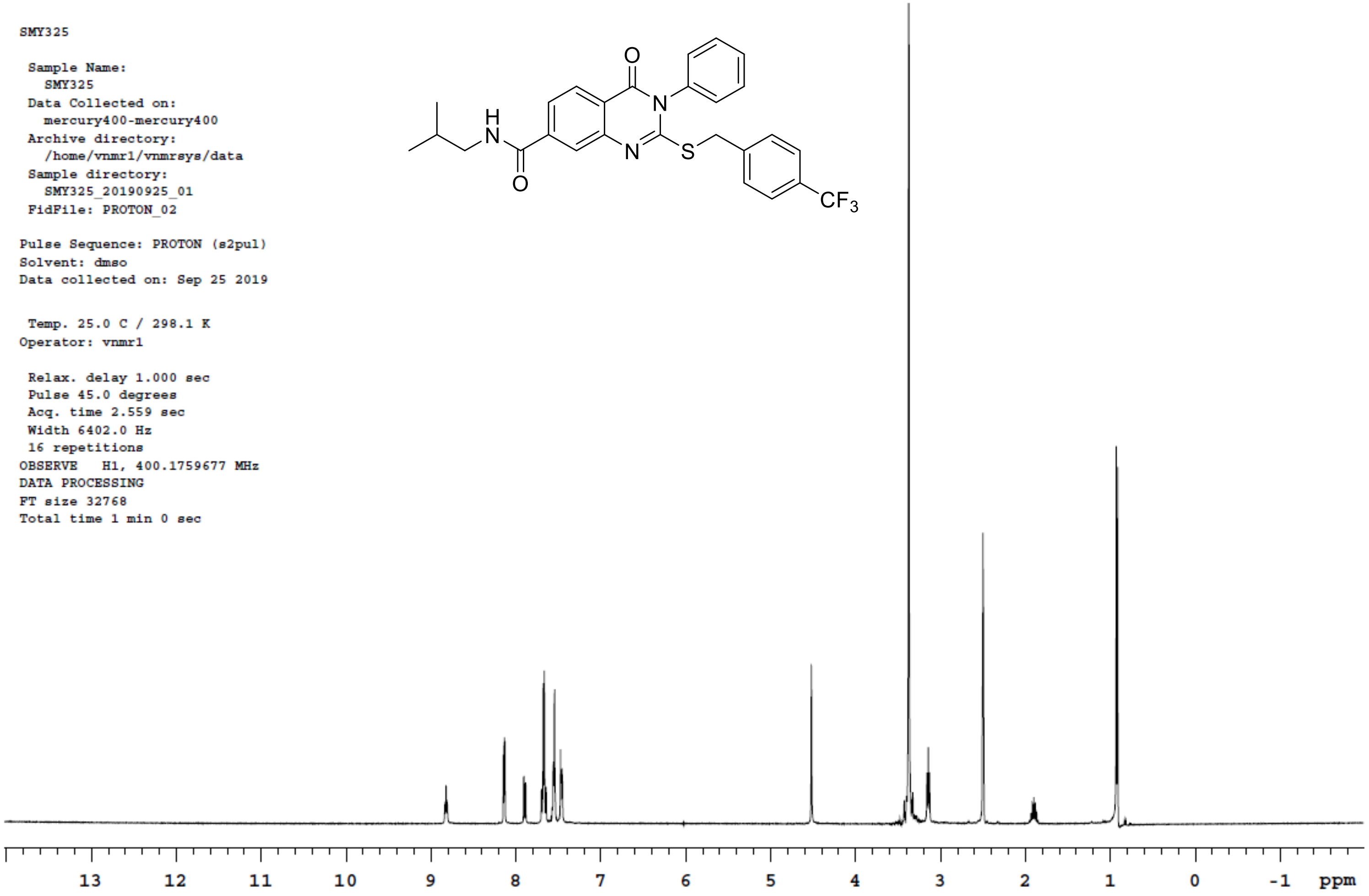

SMY325

Sample Name:
SMY325
Data Collected on:
mercury400-mercury400
Archive directory:
/home/vnmr1/vnmrsys/data
Sample directory:
SMY325_20190925_01
FidFile: CARBON_01

Pulse Sequence: CARBON (s2pul)
Solvent: dmso
Data collected on: Sep 25 2019

Temp. 25.0 C / 298.1 K
Operator: vnmr1

Relax. delay 1.000 sec
Pulse 45.0 degrees
Acq. time 1.304 sec
Width 25125.6 Hz
5000 repetitions
OBSERVE C13, 100.6243292 MHz
DECOUPLE H1, 400.1779555 MHz
Power 38 dB
continuously on
WALTZ-16 modulated
DATA PROCESSING
Line broadening 0.5 Hz
FT size 65536
Total time 3 hr, 19 min

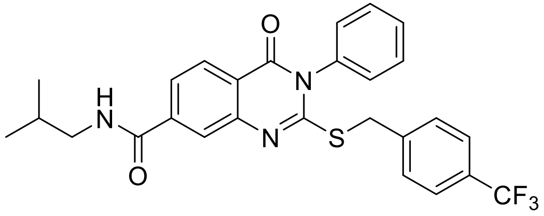

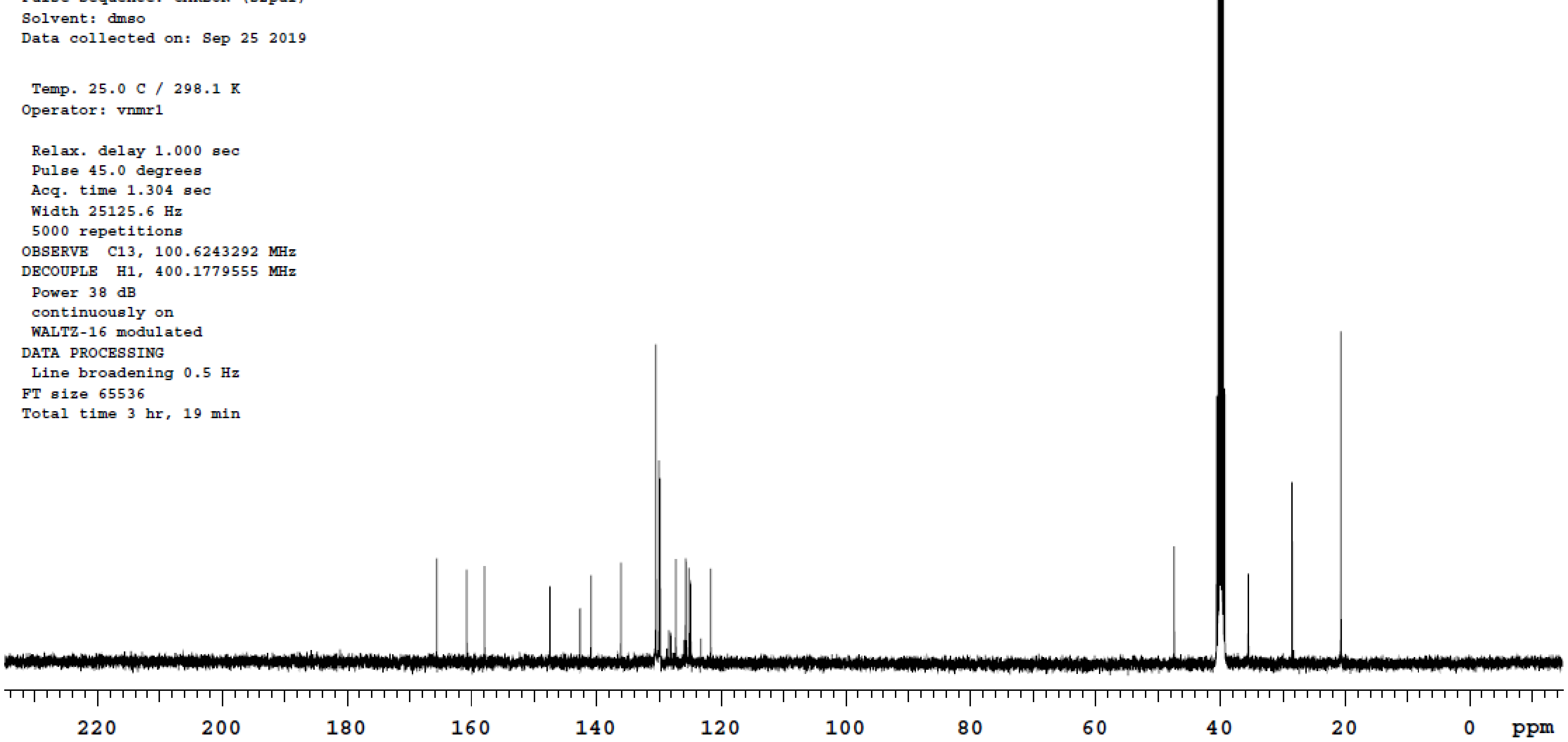

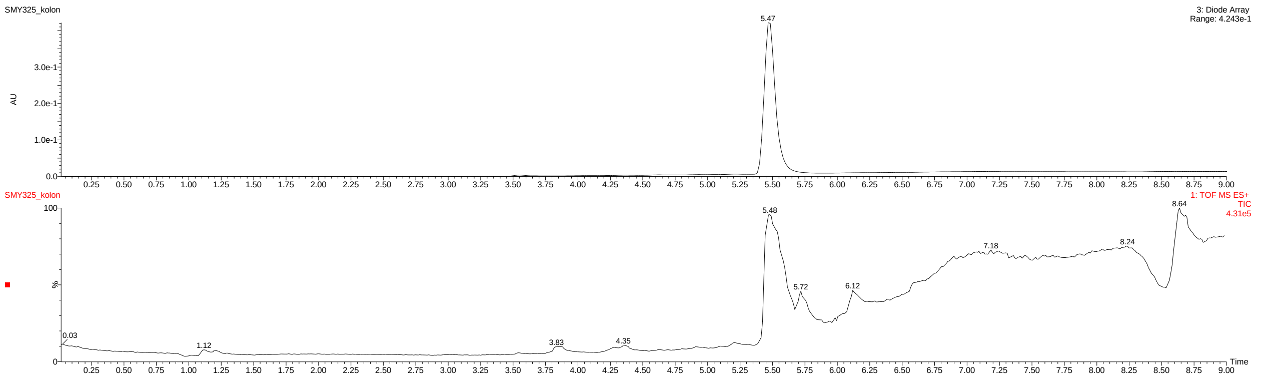

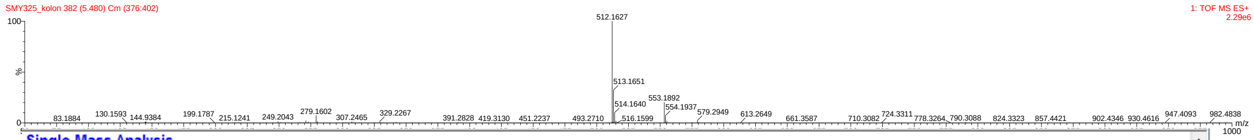

Single Mass Analysis

Tolerance = 5.0 PPM / DBE: min = -1.5, max = 50.0

Element prediction: Off

Number of isotope peaks used for i-FIT = 3

Monoisotopic Mass, Even Electron Ions

63 formula(e) evaluated with 1 results within limits (up to 50 closest results for each mass)

Elements Used:

Mass	Calc. Mass	mDa	PPM	DBE	Formula	i-FIT	i-FIT (Norm)	C	H	N	O	S	F
512.1627	512.1620	0.7	1.4	15.5	C27 H25 N3 O2 S F3	709.2	0.0	27	25	3	2	1	3

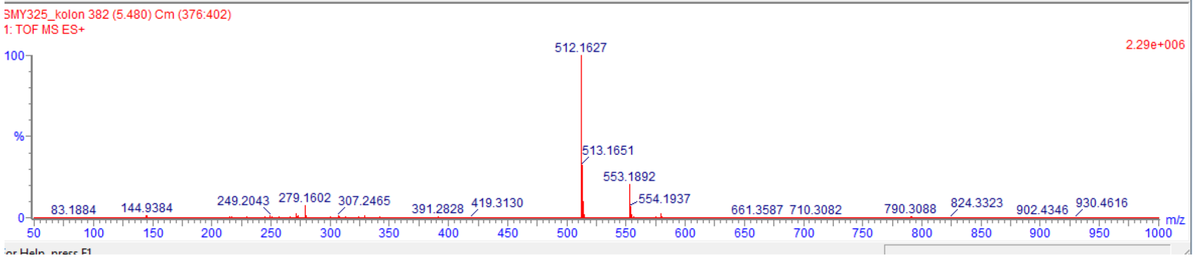

Figure S11. ^{1}H-NMR, ^{13}C-NMR and HRMS spectrums of 20

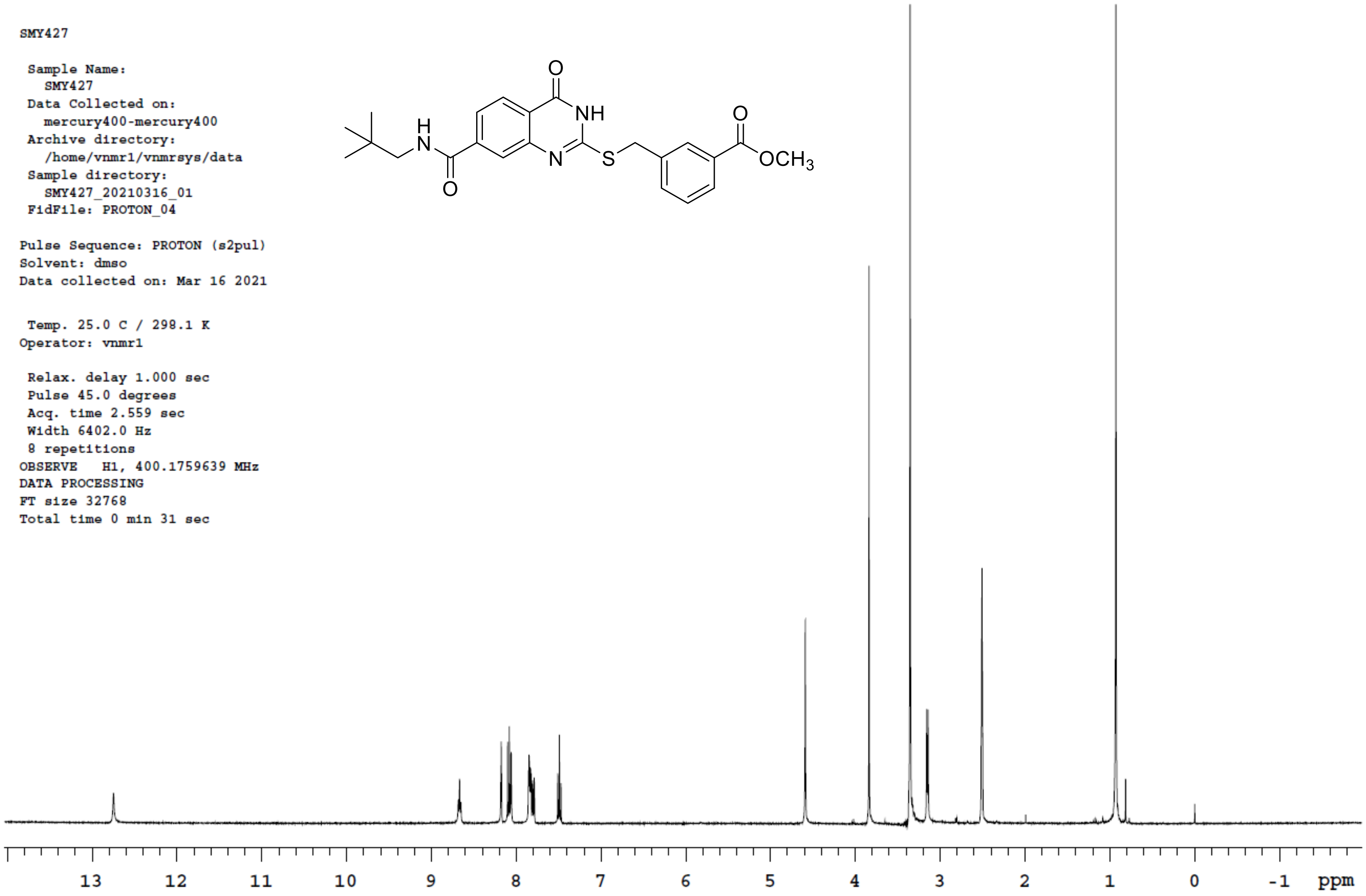

SMY427

Sample Name:
SMY427
Data Collected on:
mercury400-mercury400
Archive directory:
/home/vnmr1/vnmrsys/data
Sample directory:
SMY427_20210316_01
FidFile: current

Pulse Sequence: CARBON (s2pul)
Solvent: dmso
Data collected on: Mar 16 2021

Temp. 25.0 C / 298.1 K
Operator: vnmr1

Relax. delay 1.000 sec
Pulse 45.0 degrees
Acq. time 1.550 sec
Width 21141.6 Hz
960 repetitions
OBSERVE C13, 100.6243736 MHz
DECOUPLE H1, 400.1779555 MHz
Power 38 dB
continuously on
WALTZ-16 modulated
DATA PROCESSING
Line broadening 0.5 Hz
FT size 65536
Total time 2 hr, 3 min

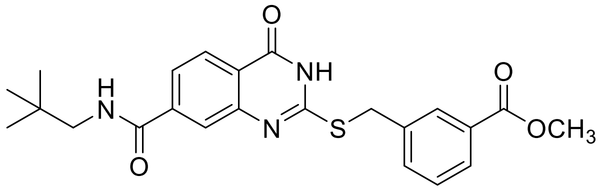

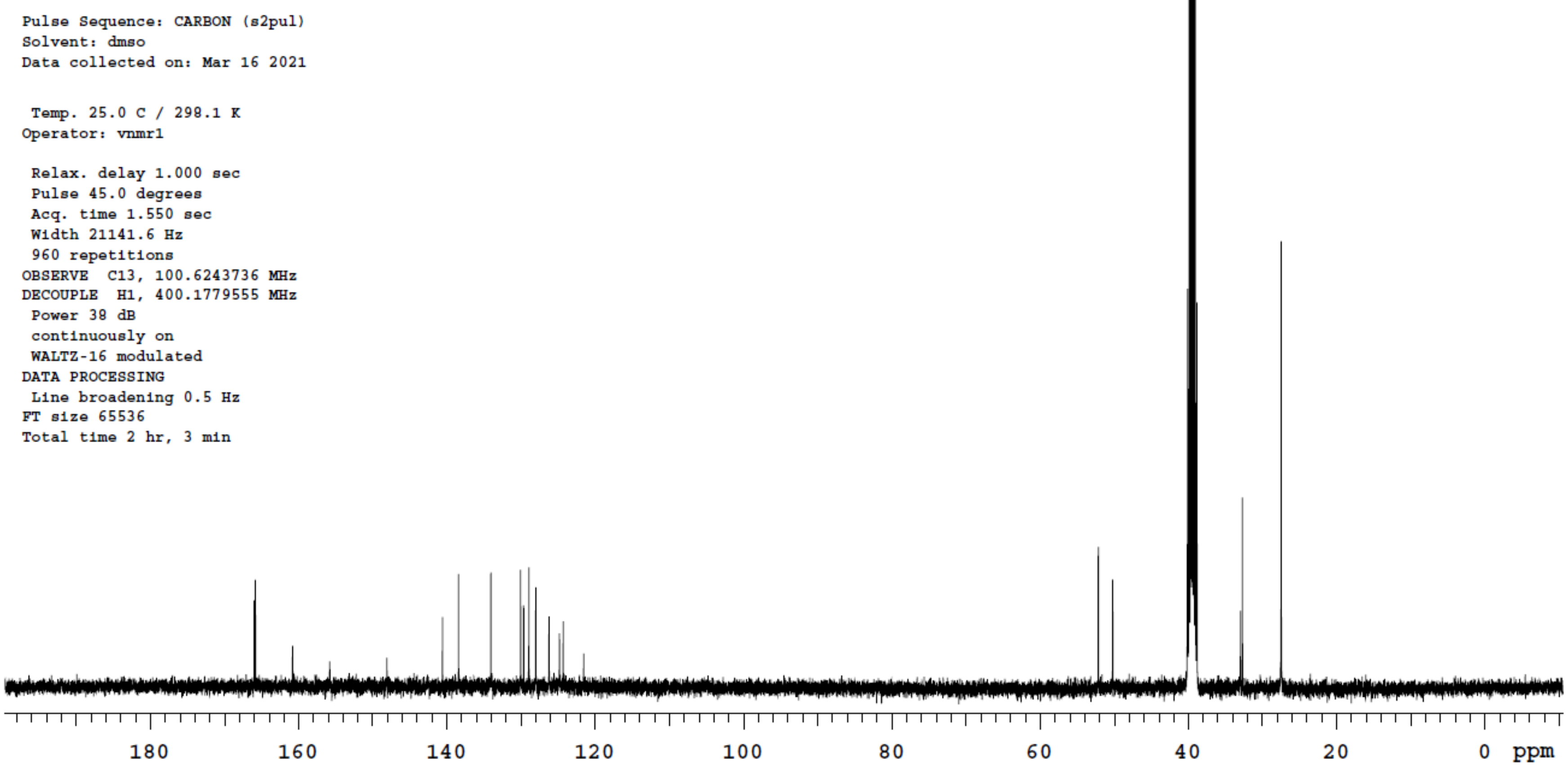

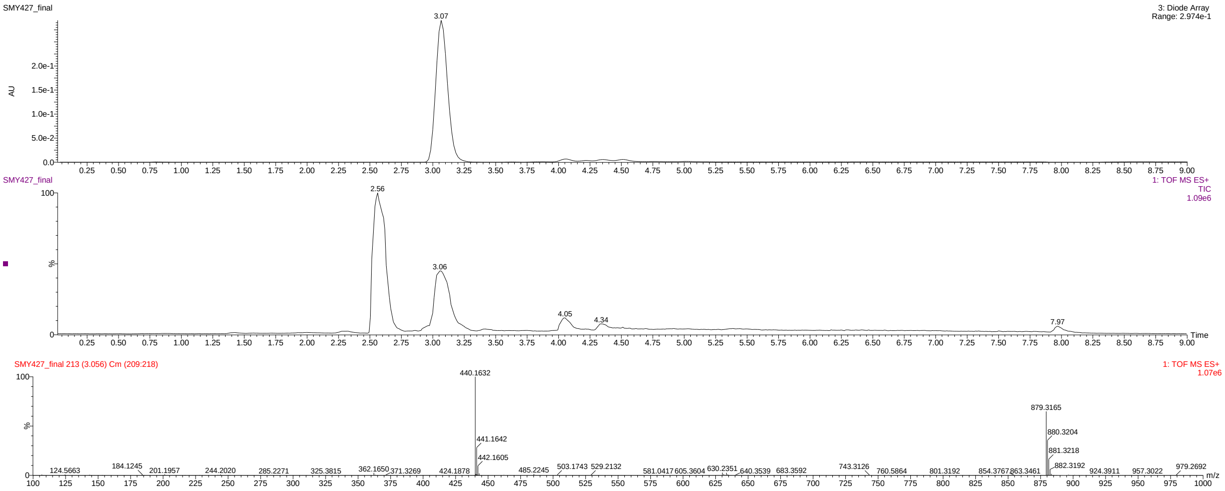

Single Mass Analysis
Tolerance = 5.0 PPM / DBE: min = -1.5, max = 50.0
Element prediction: Off
Number of isotope peaks used for i-FIT = 3
Monoisotopic Mass, Even Electron Ions
15 formula(e) evaluated with 1 results within limits (up to 50 closest results for each mass)
Elements Used:

Mass	Calc. Mass	mDa	PPM	DBE	Formula	i-FIT	i-FIT (Norm)	C	H	N	O	S
440.1632	440.1644	-1.2	-2.7	12.5	C23 H26 N3 O4 S	541.0	0.0	23	26	3	4	1

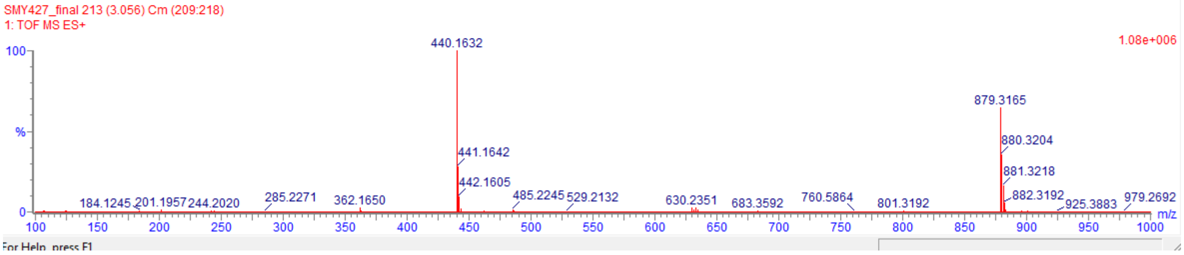

Figure S12. ^{1}H-NMR, ^{13}C-NMR and HRMS spectrums of 21

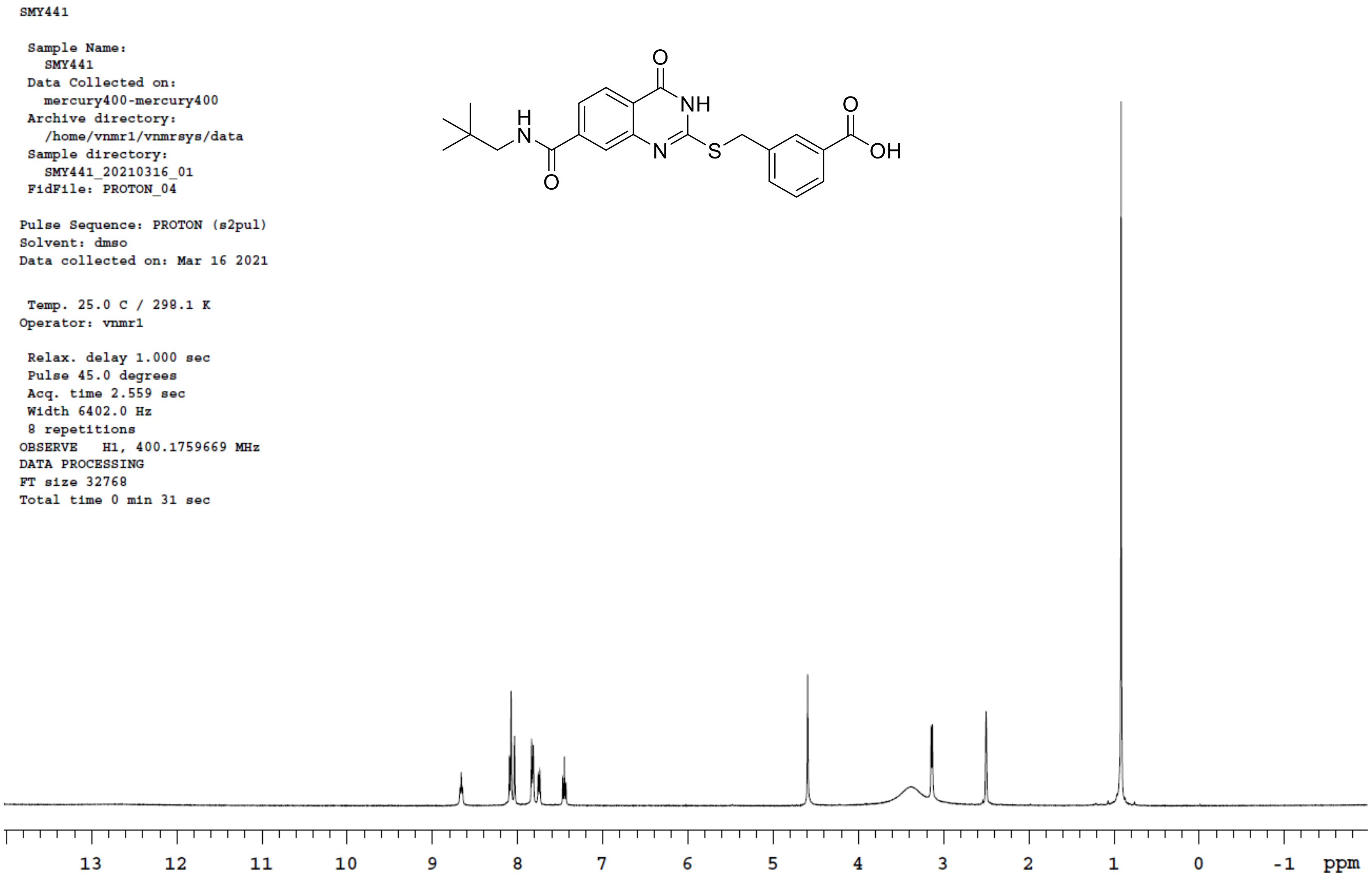

SMY441

Sample Name:
SMY441
Data Collected on:
mercury400-mercury400
Archive directory:
/home/vnmr1/vnmrsys/data
Sample directory:
SMY441_20210316_01
FidFile: CARBON_01

Pulse Sequence: CARBON (s2pul)
Solvent: dmso
Data collected on: Mar 16 2021

Temp. 25.0 C / 298.1 K
Operator: vnmr1

Relax. delay 1.000 sec
Pulse 45.0 degrees
Acq. time 1.550 sec
Width 21141.6 Hz
3000 repetitions
OBSERVE C13, 100.6243292 MHz
DECOUPLE H1, 400.1779555 MHz
Power 38 dB
continuously on
WALTZ-16 modulated
DATA PROCESSING
Line broadening 0.5 Hz
FT size 65536
Total time 2 hr, 12 min

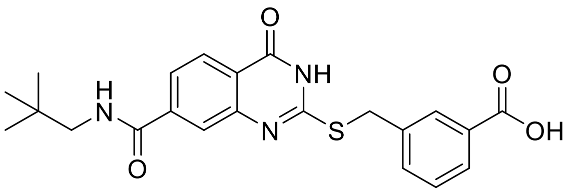

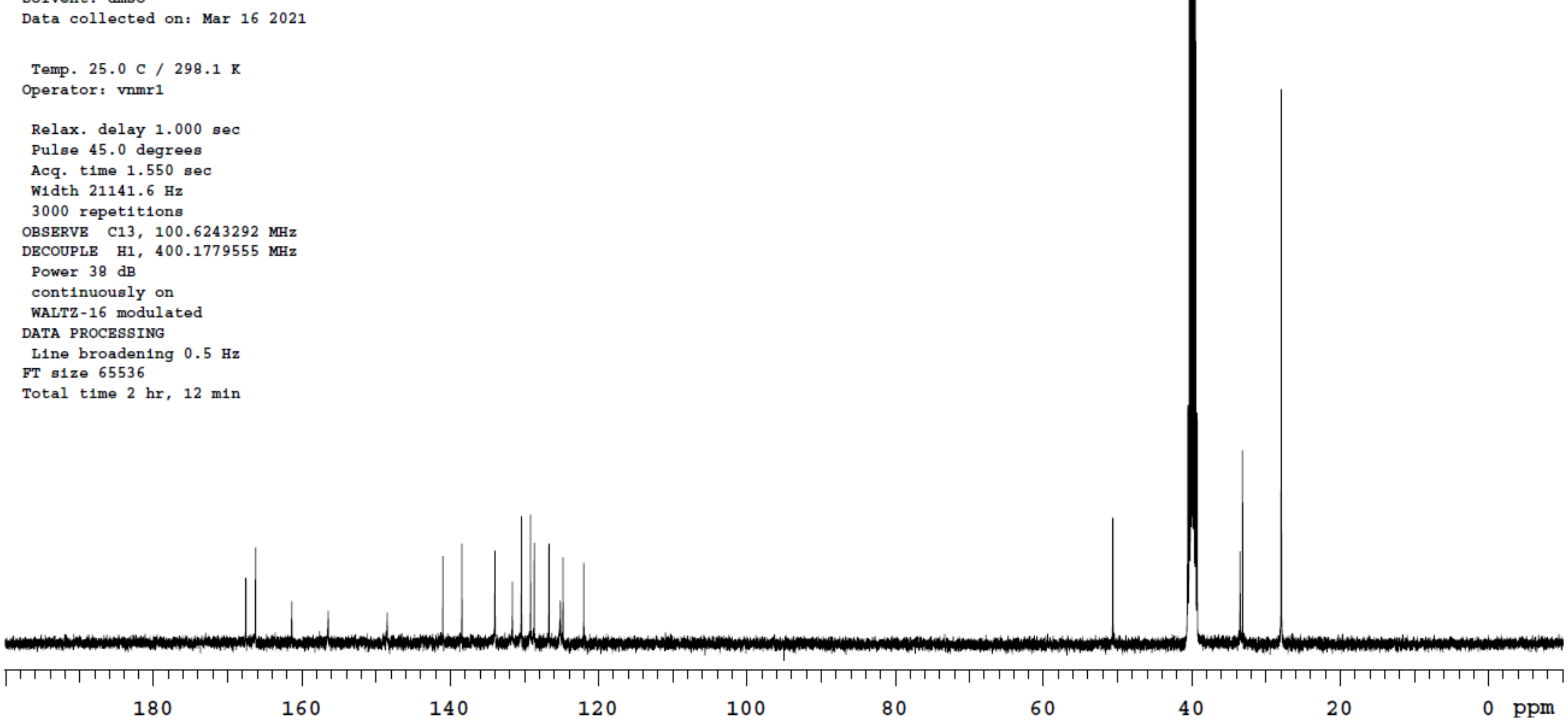

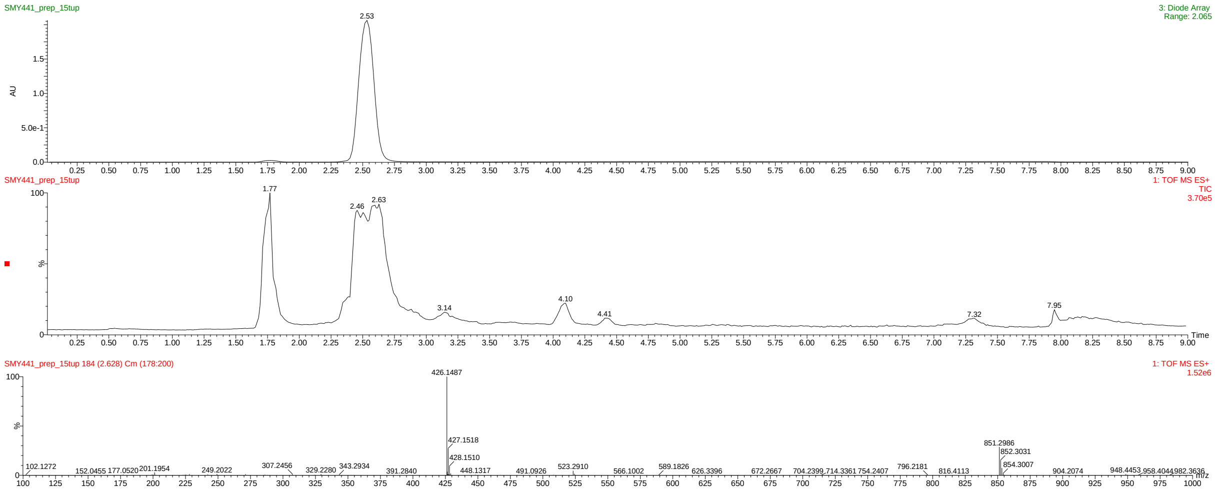

Single Mass Analysis
Tolerance = 5.0 PPM / DBE: min = -1.5, max = 50.0
Element prediction: Off
Number of isotope peaks used for i-FIT = 3
Monoisotopic Mass, Even Electron Ions
14 formula(e) evaluated with 1 results within limits (up to 50 closest results for each mass)
Elements Used:

Mass	Calc. Mass	mDa	PPM	DBE	Formula	i-FIT	i-FIT (Norm)	C	H	N	O	S
426.1487	426.1488	-0.1	-0.2	12.5	C22 H24 N3 O4 S	735.3	0.0	22	24	3	4	1

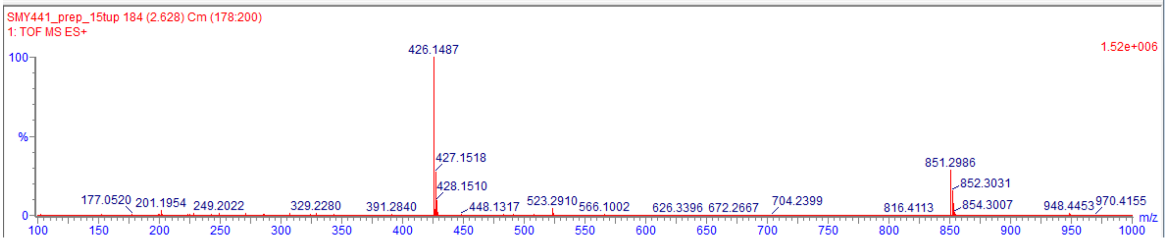

Figure S13. ^{1}H-NMR, ^{13}C-NMR and HRMS spectrums of 34

```
AGU282

 Sample Name:
   AGU282
 Data Collected on:
   mercury400-mercury400
 Archive directory:
   /home/vnmr1/vnmrsys/data
 Sample directory:
   AGU282_20191002_01
 FidFile: PROTON_02

Pulse Sequence: PROTON (s2pul)
Solvent: dmso
Data collected on: Oct  2 2019

 Temp. 25.0 C / 298.1 K
Operator: vnmr1

 Relax. delay 1.000 sec
 Pulse 45.0 degrees
 Acq. time 2.559 sec
 Width 6402.0 Hz
 8 repetitions
OBSERVE   H1, 400.1759761 MHz
DATA PROCESSING
FT size 32768
Total time 0 min 31 sec
```

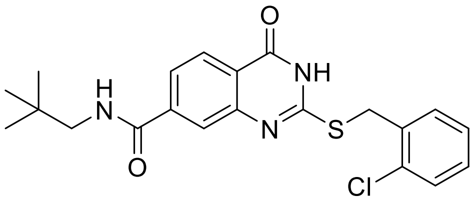

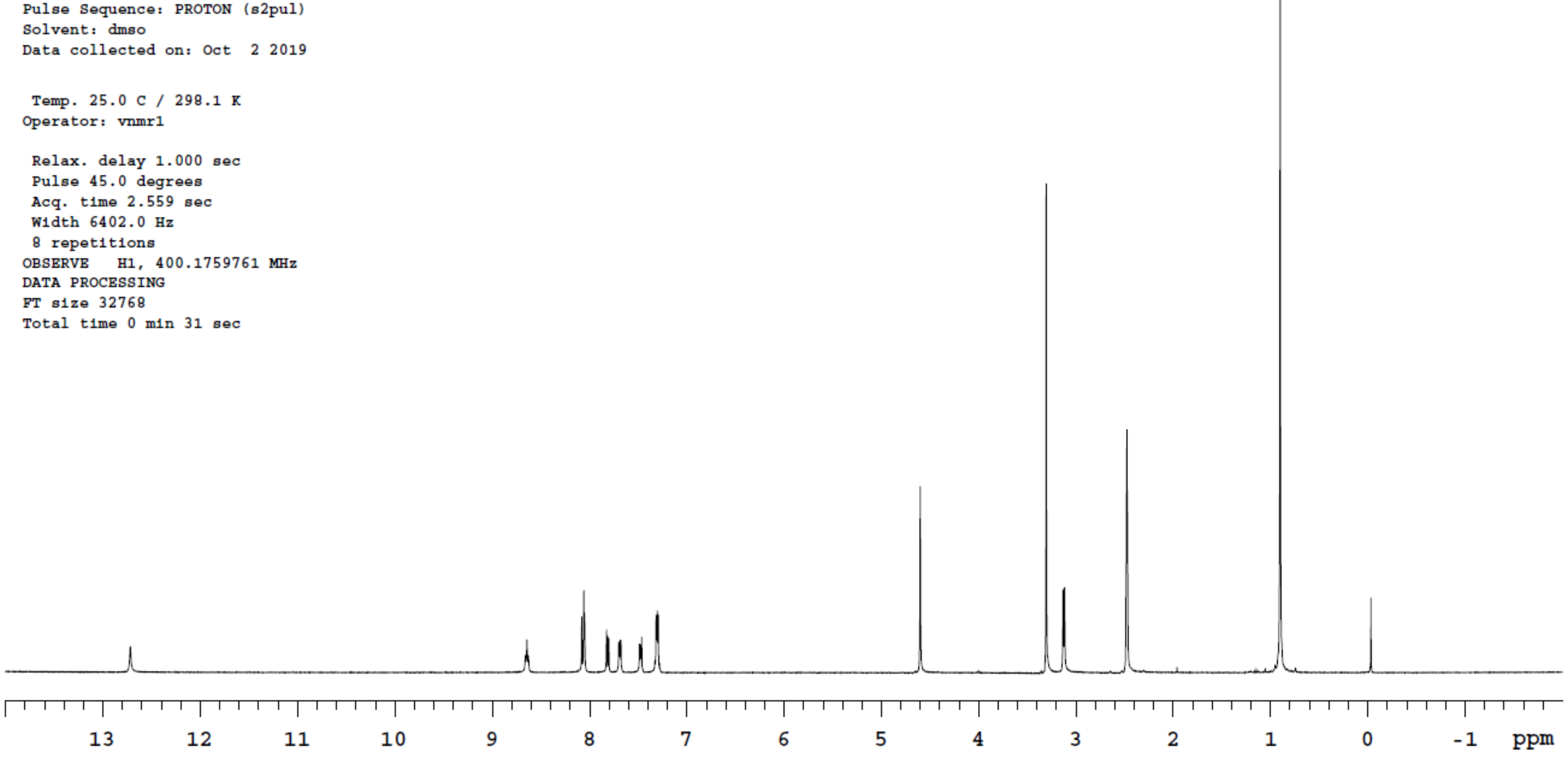

CARBON_01
AGU282

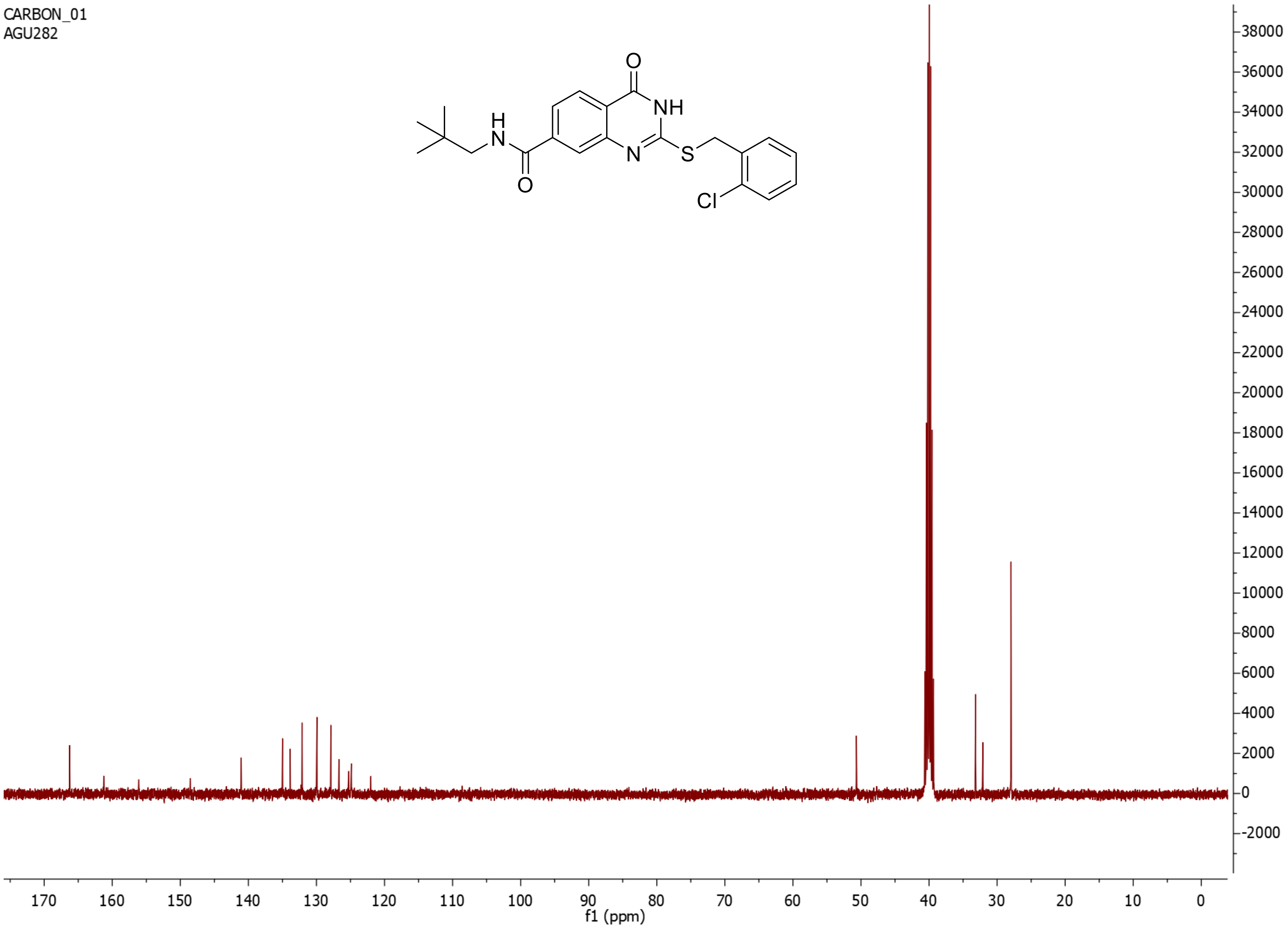

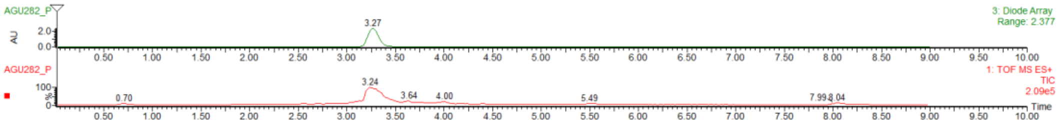

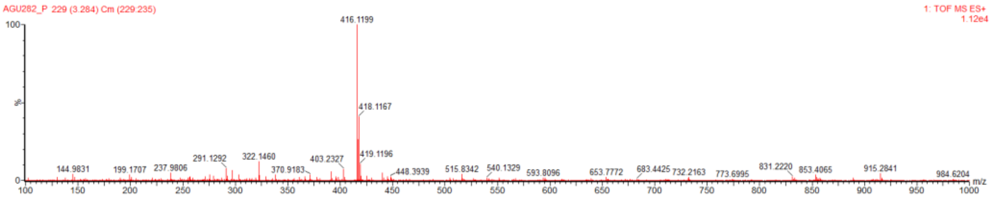

Single Mass Analysis

Tolerance = 5.0 PPM / DBE: min = -1.5, max = 50.0

Element prediction: Off

Number of isotope peaks used for i-FIT = 3

Monoisotopic Mass, Even Electron Ions

109 formula(e) evaluated with 1 results within limits (up to 50 closest results for each mass)

Elements Used:

Mass	Calc. Mass	mDa	PPM	DBE	Formula	i-FIT	i-FIT (Norm)	C	H	N	O	S	Cl
416.1199	416.1200	-0.1	-0.2	11.5	C21 H23 N3 O2 S Cl	199.8	0.0	21	23	3	2	1	1

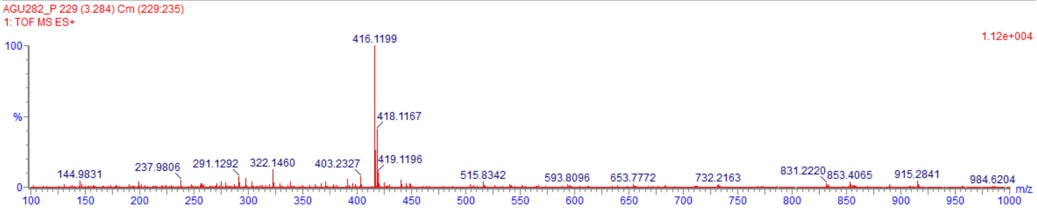

Figure S14. ^{1}H-NMR, ^{13}C-NMR and HRMS spectrums of 35

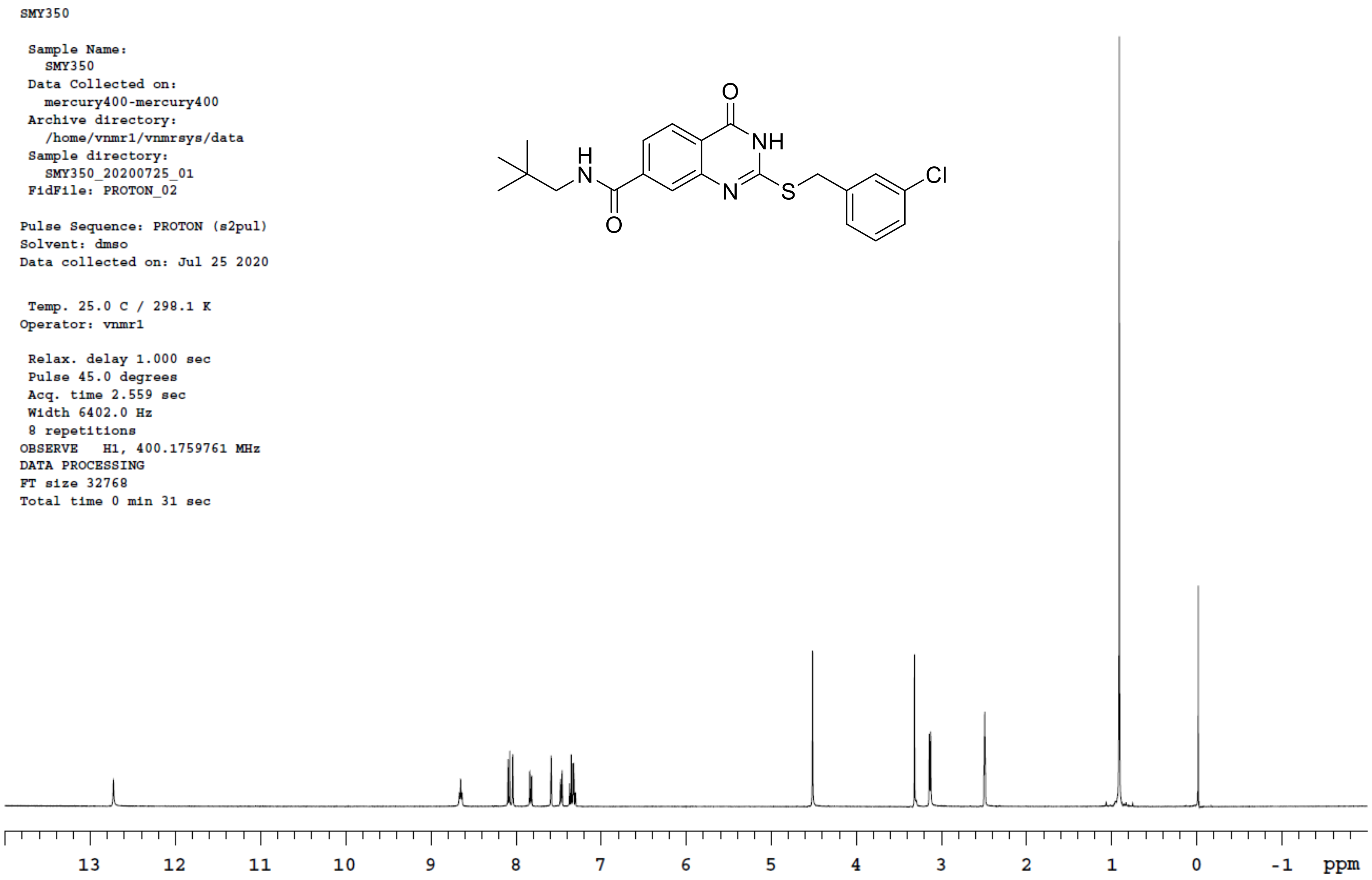

SMY350

Sample Name:
SMY350
Data Collected on:
mercury400-mercury400
Archive directory:
/home/vnmr1/vnmrsys/data
Sample directory:
SMY350_20200725_01
FidFile: CARBON_01

Pulse Sequence: CARBON (s2pul)
Solvent: dmso
Data collected on: Jul 25 2020

Temp. 25.0 C / 298.1 K
Operator: vnmr1

Relax. delay 1.000 sec
Pulse 45.0 degrees
Acq. time 1.478 sec
Width 22172.9 Hz
2000 repetitions
OBSERVE C13, 100.6243851 MHz
DECOUPLE H1, 400.1779555 MHz
Power 38 dB
continuously on
WALTZ-16 modulated
DATA PROCESSING
Line broadening 0.5 Hz
FT size 65536
Total time 1 hr, 25 min

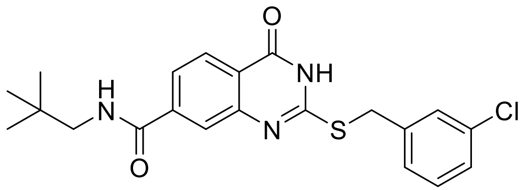

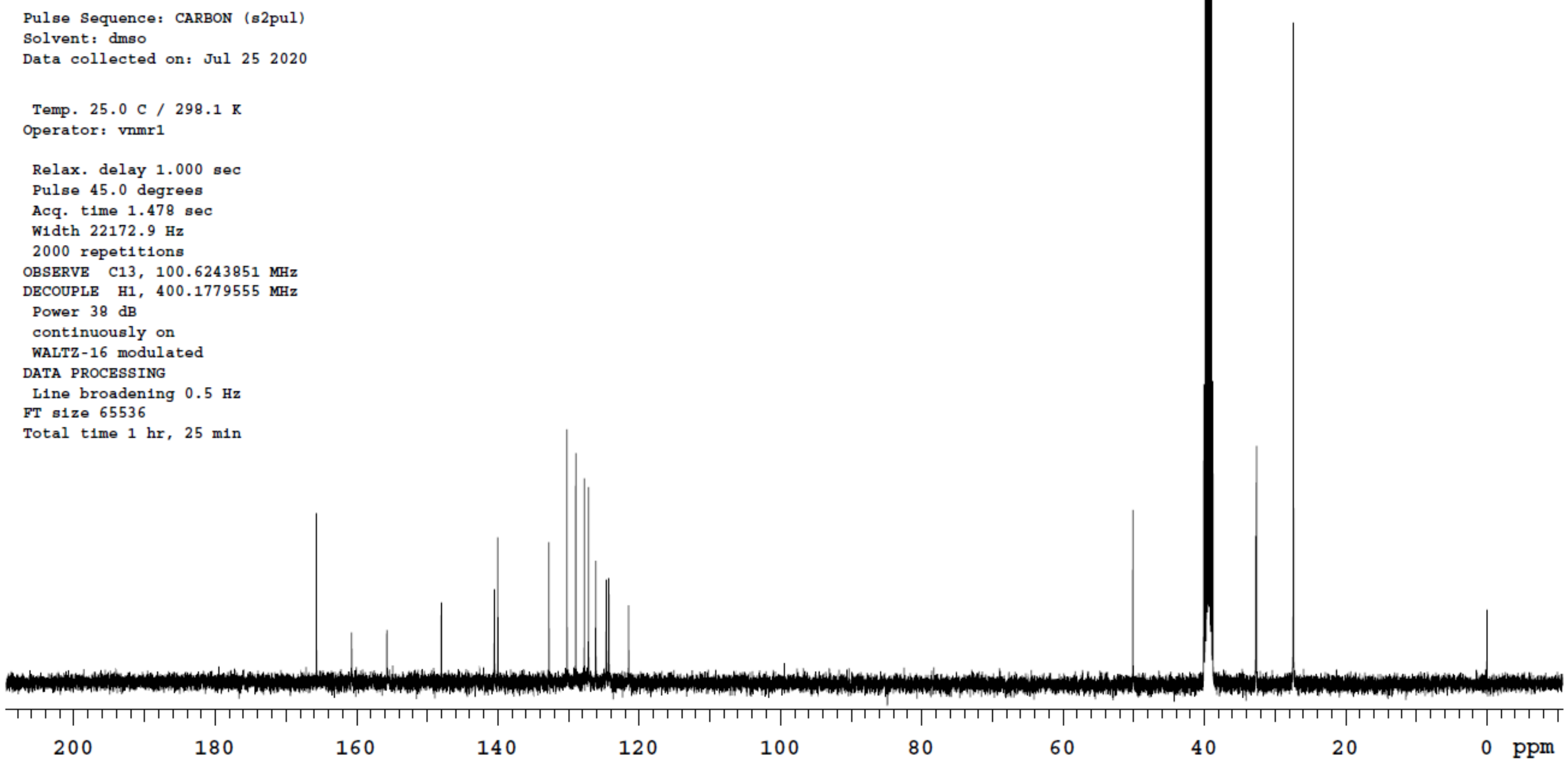

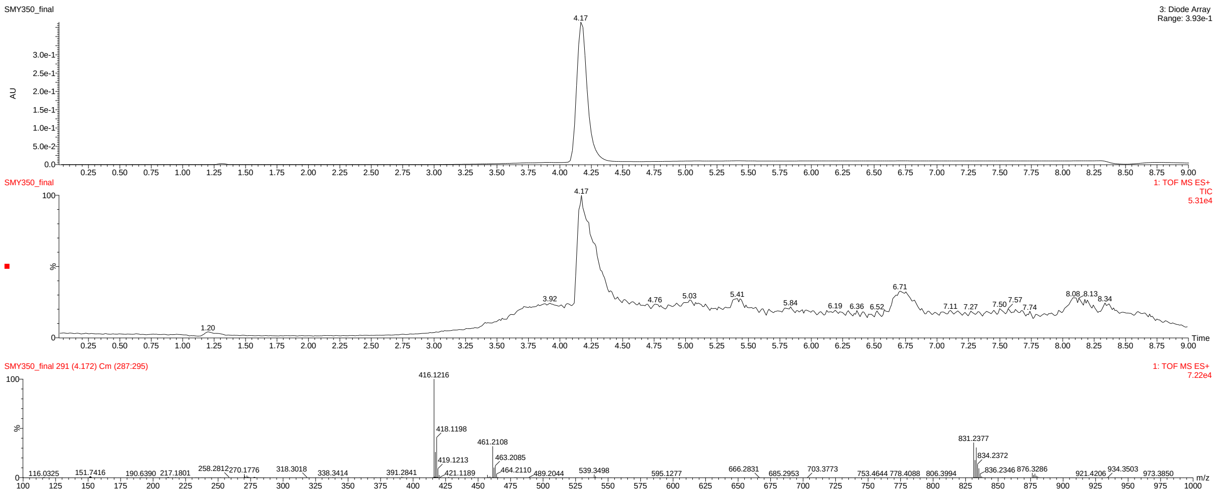

Single Mass Analysis

Tolerance = 5.0 PPM / DBE: min = -1.5, max = 50.0

Element prediction: Off

Number of isotope peaks used for i-FIT = 3

Monoisotopic Mass, Even Electron Ions

96 formula(e) evaluated with 1 results within limits (up to 50 closest results for each mass)

Elements Used:

Mass	Calc. Mass	mDa	PPM	DBE	Formula	i-FIT	i-FIT (Norm)	C	H	N	O	S	Cl
416.1216	416.1200	1.6	3.8	11.5	C21 H23 N3 O2 S Cl	378.9	0.0	21	23	3	2	1	1

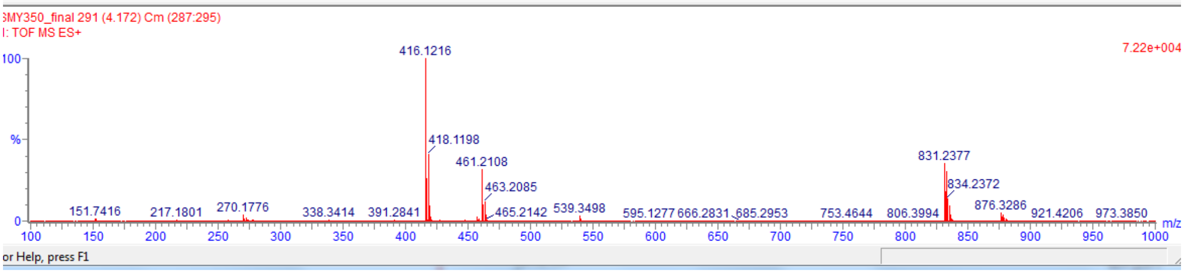

or Help, press F1

Figure S15. ^{1}H-NMR, ^{13}C-NMR and HRMS spectrums of 36

SMY504

Sample Name:
SMY504
Data Collected on:
mercury400-mercury400
Archive directory:
/home/vnmr1/vnmrsys/data
Sample directory:
SMY504_20220430_01
FidFile: PROTON_01

Pulse Sequence: PROTON (s2pul)
Solvent: dmso
Data collected on: Apr 30 2022

Temp. 35.0 C / 308.1 K
Operator: vnmr1

Relax. delay 1.000 sec
Pulse 45.0 degrees
Acq. time 2.559 sec
Width 6402.0 Hz
8 repetitions
OBSERVE H1, 400.1759761 MHz
DATA PROCESSING
FT size 32768
Total time 0 min 31 sec

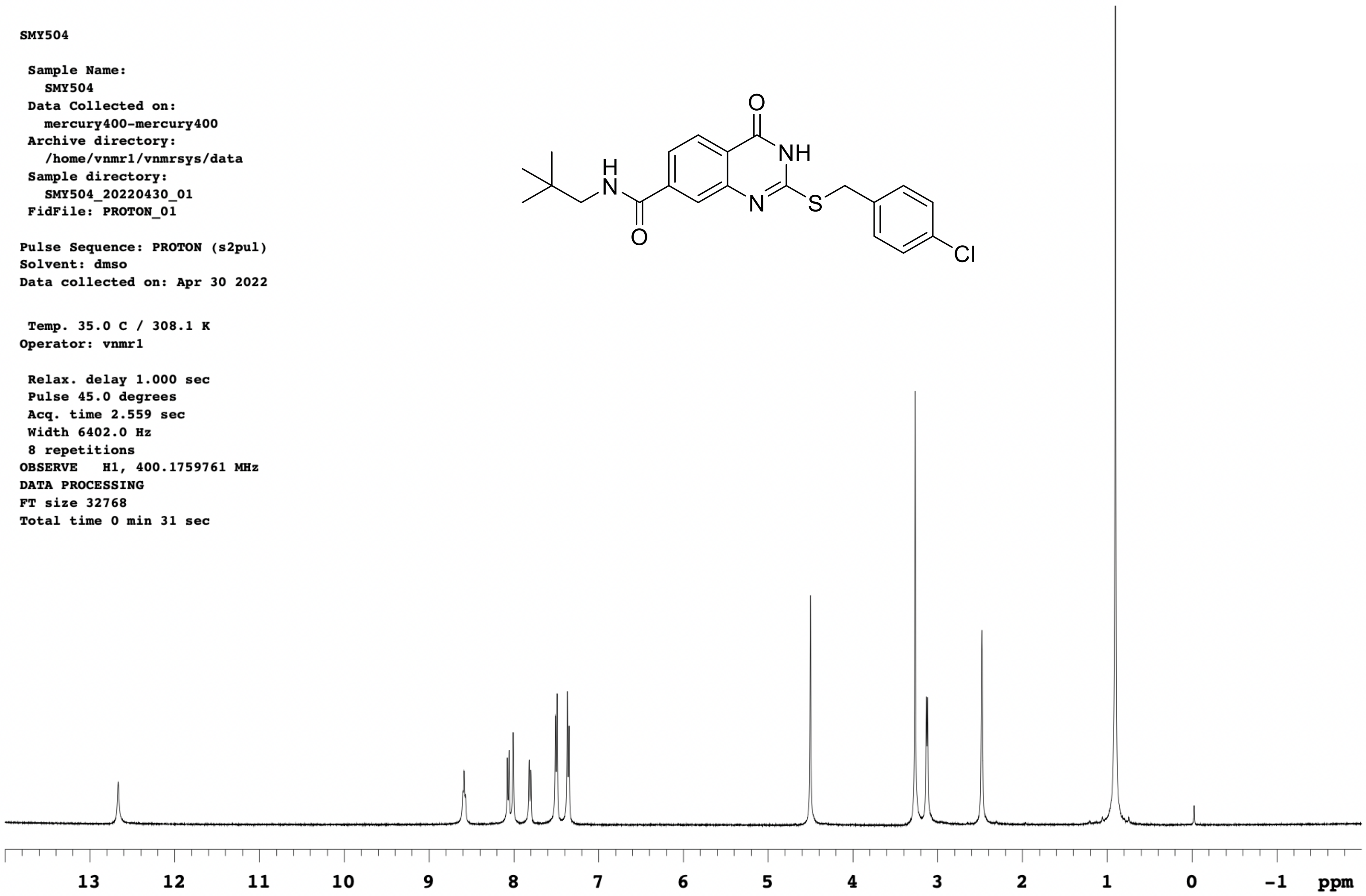

SMY504

Sample Name:
SMY504
Data Collected on:
mercury400-mercury400
Archive directory:
/home/vnmr1/vnmrsys/data
Sample directory:
SMY504_20220430_01
FidFile: CARBON_02

Pulse Sequence: CARBON (s2pul)
Solvent: dmso
Data collected on: Apr 30 2022

Temp. 35.0 C / 308.1 K
Operator: vnmr1

Relax. delay 1.000 sec
Pulse 45.0 degrees
Acq. time 1.550 sec
Width 21141.6 Hz
4000 repetitions
OBSERVE C13, 100.6243846 MHz
DECOUPLE H1, 400.1779555 MHz
Power 38 dB
continuously on
WALTZ-16 modulated
DATA PROCESSING
Line broadening 0.5 Hz
FT size 65536
Total time 2 hr, 56 min

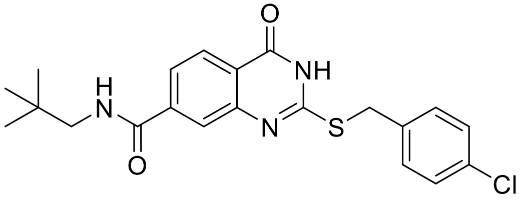

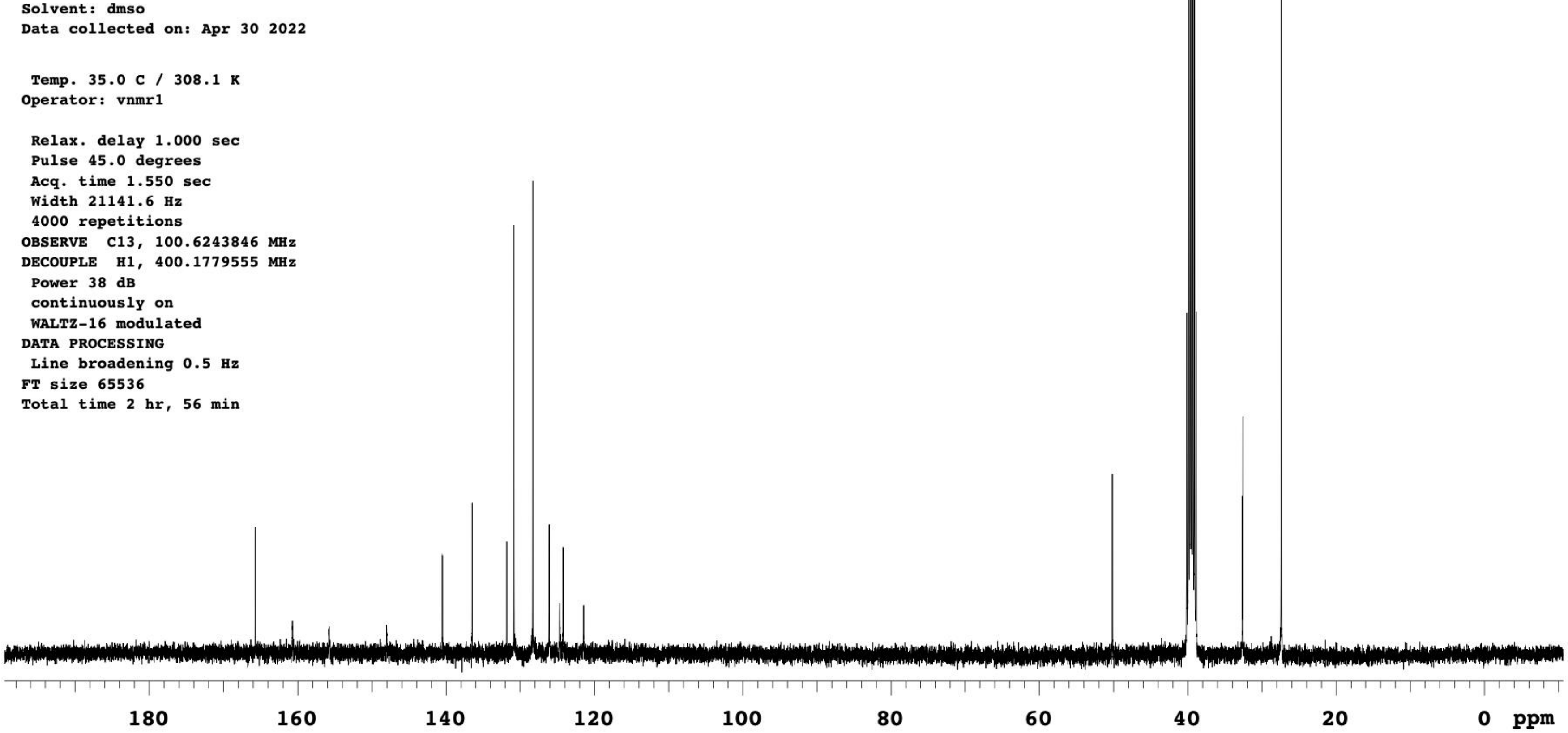

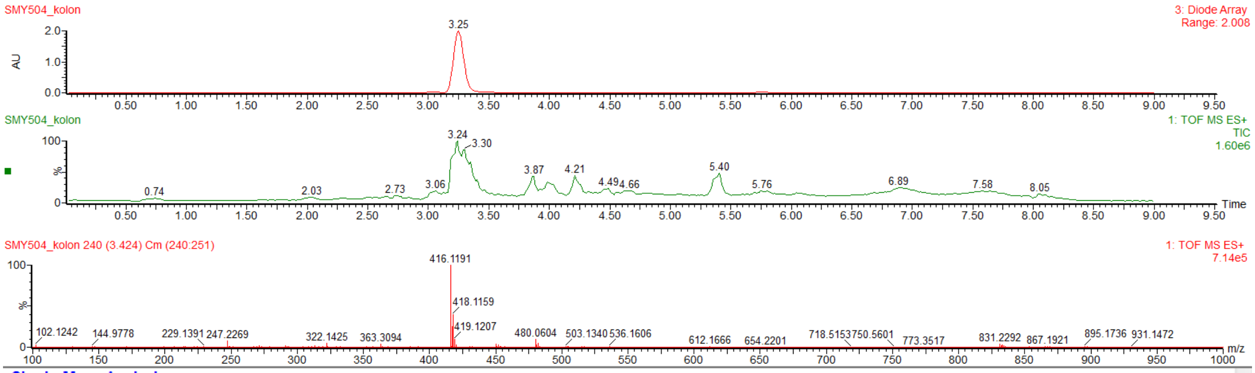

Single Mass Analysis

Tolerance = 50.0 PPM / DBE: min = -1.5, max = 50.0

Element prediction: Off

Number of isotope peaks used for i-FIT = 3

Monoisotopic Mass, Even Electron Ions

55 formula(e) evaluated with 1 results within limits (up to 50 closest results for each mass)

Elements Used:

Mass	Calc. Mass	mDa	PPM	DBE	Formula	i-FIT	i-FIT (Norm)	C	H	N	O	S	Cl
416.1191	416.1200	-0.9	-2.2	11.5	C21 H23 N3 O2 S Cl	607.3	0.0	21	23	3	2	1	1

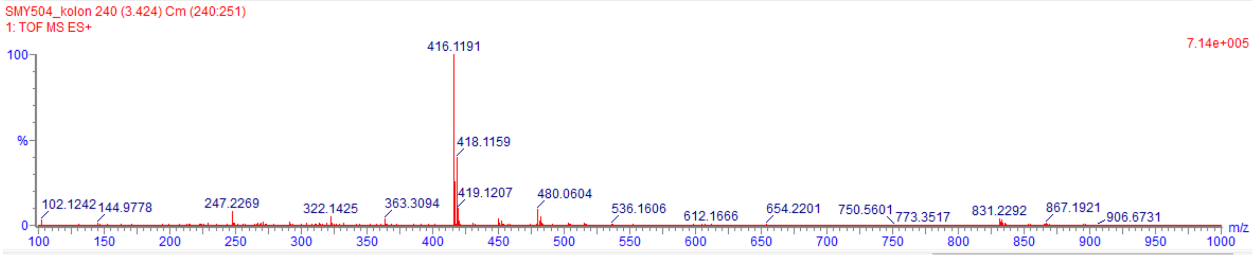

Figure S16. ^{1}H-NMR, ^{13}C-NMR and HRMS spectrums of 37

SMY352

Sample Name:
SMY352
Data Collected on:
mercury400-mercury400
Archive directory:
/home/vnmr1/vnmrsys/data
Sample directory:
SMY352_20200725_01
FidFile: PROTON_02

Pulse Sequence: PROTON (s2pul)
Solvent: dmso
Data collected on: Jul 25 2020

Temp. 25.0 C / 298.1 K
Operator: vnmr1

Relax. delay 1.000 sec
Pulse 45.0 degrees
Acq. time 2.559 sec
Width 6402.0 Hz
8 repetitions
OBSERVE H1, 400.1759761 MHz
DATA PROCESSING
FT size 32768
Total time 0 min 31 sec

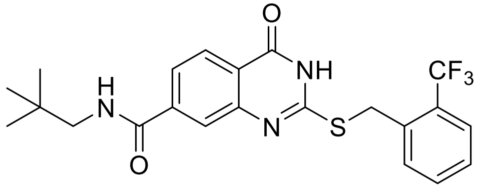

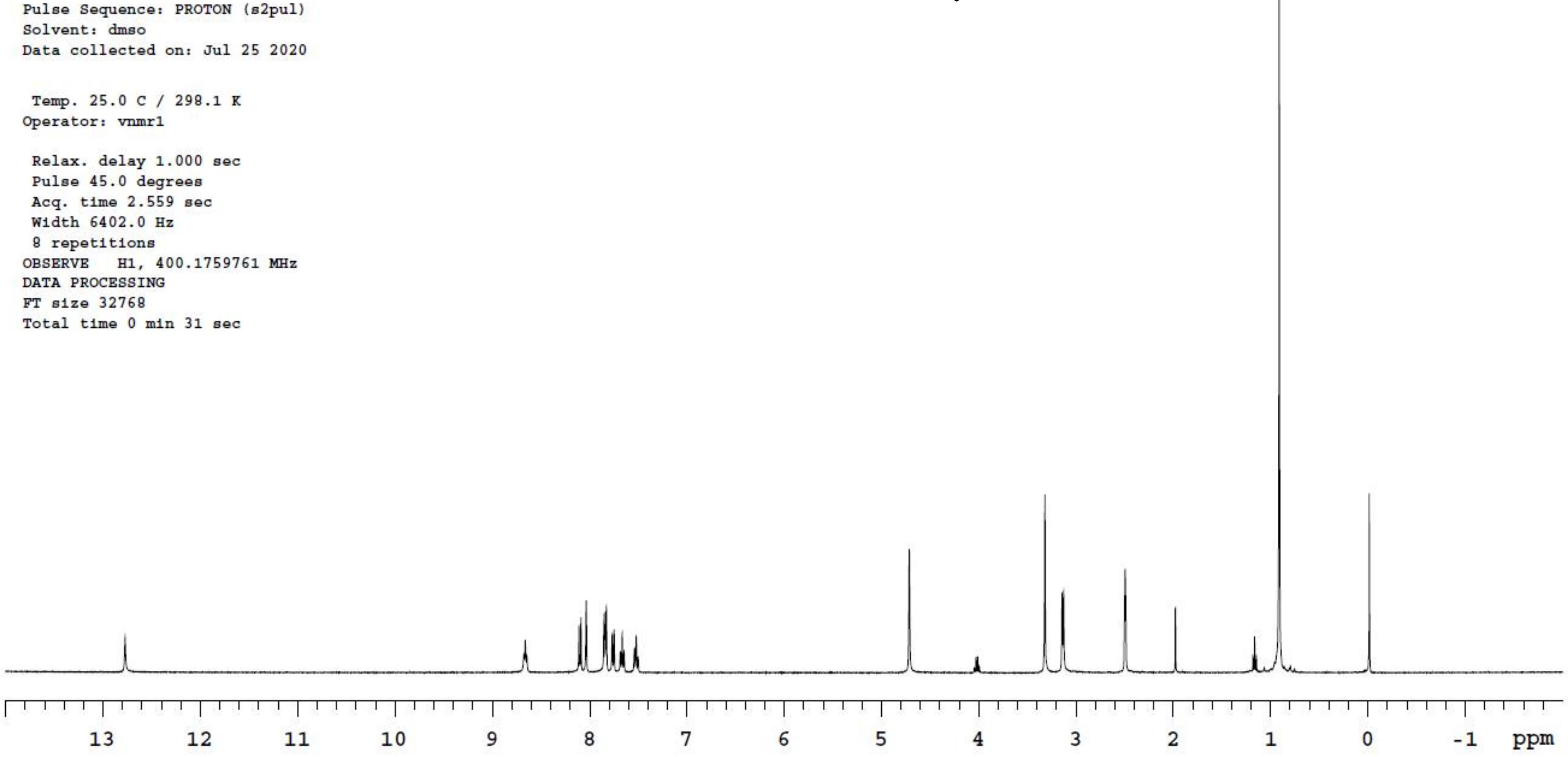

SMY352

Sample Name:
SMY352
Data Collected on:
mercury400-mercury400
Archive directory:
/home/vnmr1/vnmrsys/data
Sample directory:
SMY352_20200725_01
FidFile: CARBON_01

Pulse Sequence: CARBON (s2pul)
Solvent: dmso
Data collected on: Jul 25 2020

Temp. 25.0 C / 298.1 K
Operator: vnmr1

Relax. delay 1.000 sec
Pulse 45.0 degrees
Acq. time 1.550 sec
Width 21141.6 Hz
2000 repetitions
OBSERVE C13, 100.6243842 MHz
DECOUPLE H1, 400.1779555 MHz
Power 38 dB
continuously on
WALTZ-16 modulated
DATA PROCESSING
Line broadening 0.5 Hz
FT size 65536
Total time 1 hr, 28 min

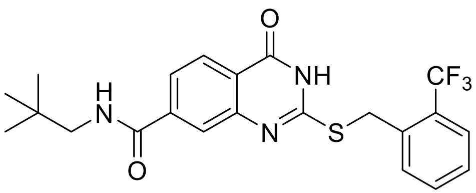

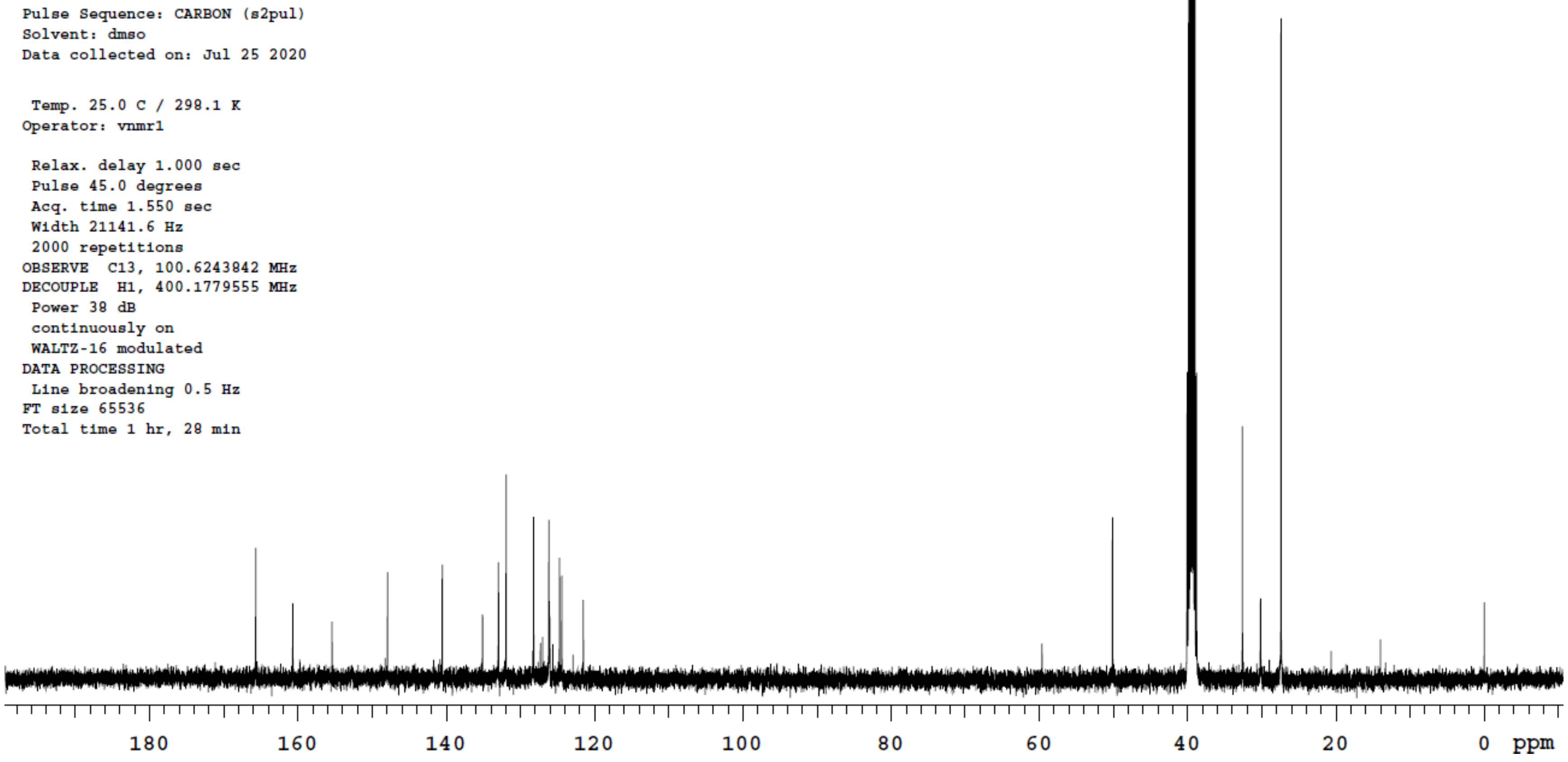

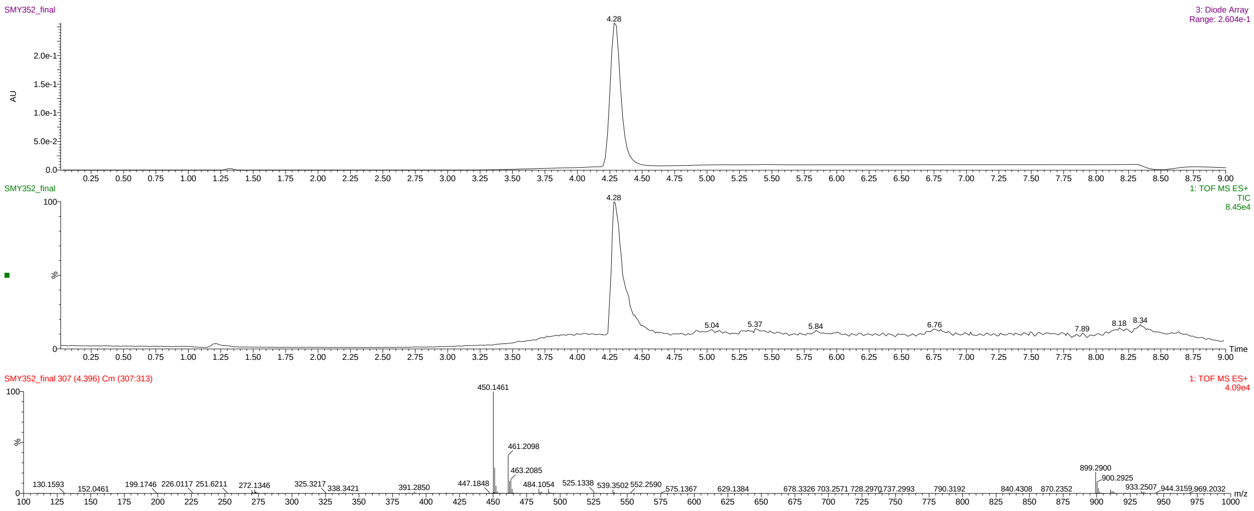

Single Mass Analysis
Tolerance = 5.0 PPM / DBE: min = -1.5, max = 50.0
Element prediction: Off
Number of isotope peaks used for i-FIT = 3
Monoisotopic Mass, Even Electron Ions
76 formula(e) evaluated with 1 results within limits (up to 50 closest results for each mass)
Elements Used:

Mass	Calc. Mass	mDa	PPM	DBE	Formula	i-FIT	i-FIT (Norm)	C	H	N	O	F	S
450.1461	450.1463	-0.2	-0.4	11.5	C22 H23 N3 O2 F3 S	357.5	0.0	22	23	3	2	3	1

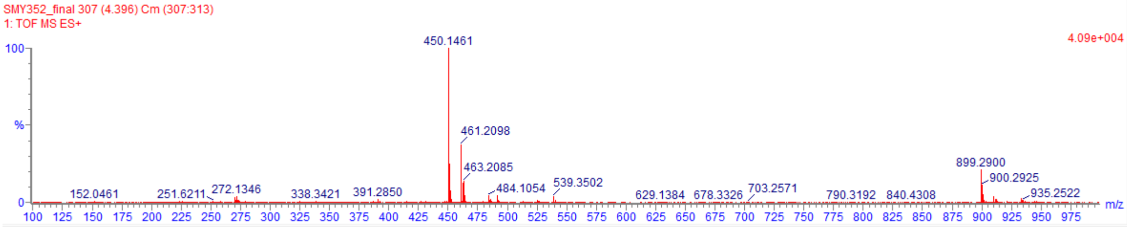

For Help, press F1

Figure S17. 1H-NMR, 13C-NMR and HRMS spectrums of 38

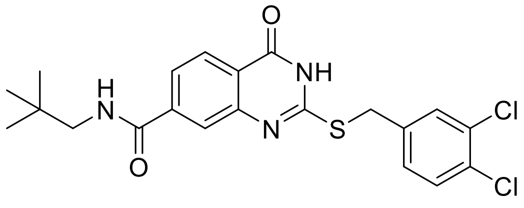

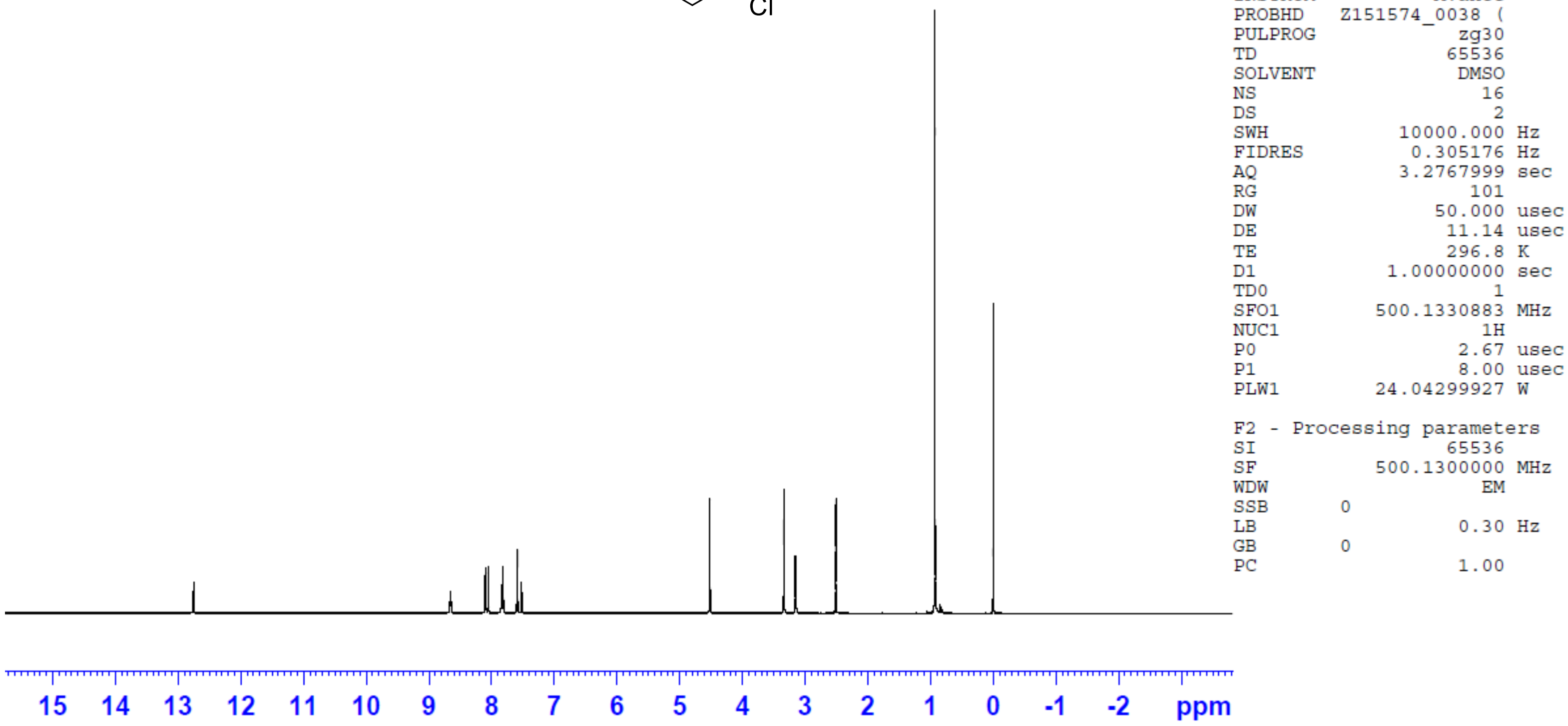


```
Current Data Parameters
NAME               SMY356
EXPNO                   1
PROCNO                  1

F2 - Acquisition Parameters
Date_            20200725
Time                16.52 h
INSTRUM            Avance
PROBHD   Z151574_0038 (
PULPROG              zg30
TD                  65536
SOLVENT              DMSO
NS                     16
DS                      2
SWH             10000.000 Hz
FIDRES           0.305176 Hz
AQ              3.2767999 sec
RG                    101
DW                 50.000 usec
DE                  11.14 usec
TE                  296.8 K
D1             1.00000000 sec
TD0                     1
SFO1          500.1330883 MHz
NUC1                   1H
P0                   2.67 usec
P1                   8.00 usec
PLW1          24.04299927 W

F2 - Processing parameters
SI                  65536
SF            500.1300000 MHz
WDW                    EM
SSB        0
LB                   0.30 Hz
GB         0
PC                   1.00
```

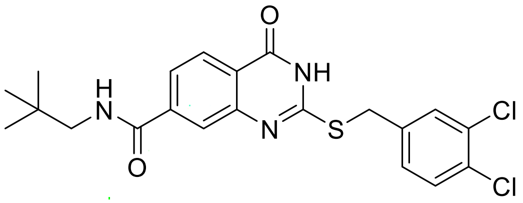

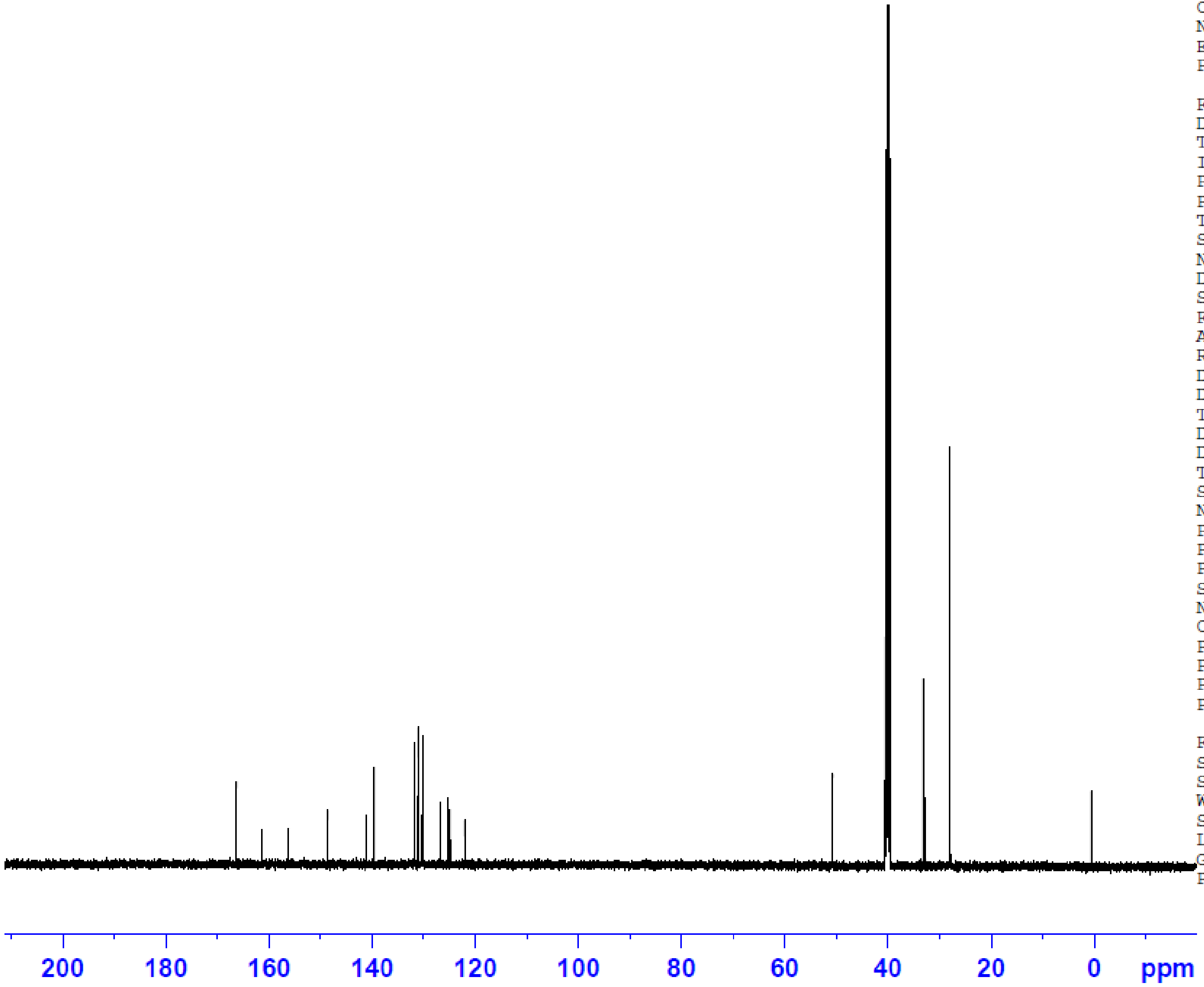


```
Current Data Parameters
NAME              SMY356
EXPNO                  2
PROCNO                 1

F2 - Acquisition Parameters
Date_           20200725
Time               18.12 h
INSTRUM           Avance
PROBHD   Z151574_0038 (
PULPROG           zgpg30
TD                 65536
SOLVENT             DMSO
NS                  1500
DS                     4
SWH            30120.482 Hz
FIDRES          0.919204 Hz
AQ             1.0878977 sec
RG                   101
DW                16.600 usec
DE                  6.50 usec
TE                 298.1 K
D1            2.00000000 sec
D11           0.03000000 sec
TD0                    1
SFO1         125.7703643 MHz
NUC1                 13C
P0                  3.33 usec
P1                 10.00 usec
PLW1         85.18099976 W
SFO2         500.1320005 MHz
NUC2                  1H
CPDPRG[2          waltz65
PCPD2              80.00 usec
PLW2         24.04299927 W
PLW12         0.24043000 W
PLW13         0.12093000 W

F2 - Processing parameters
SI                 32768
SF           125.7577885 MHz
WDW                   no
SSB       0
LB        0 Hz
GB        0
PC                  1.40
```

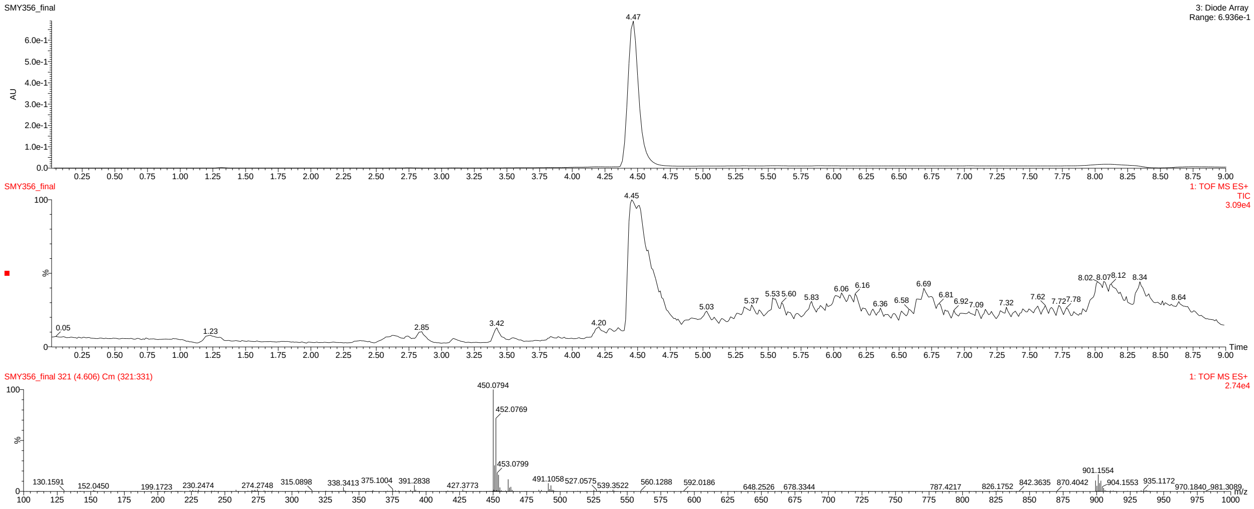

Single Mass Analysis
Tolerance = 5.0 PPM / DBE: min = -1.5, max = 50.0
Element prediction: Off
Number of isotope peaks used for i-FIT = 3
Monoisotopic Mass, Even Electron Ions
67 formula(e) evaluated with 1 results within limits (up to 50 closest results for each mass)
Elements Used:

Mass	Calc. Mass	mDa	PPM	DBE	Formula	i-FIT	i-FIT (Norm)	C	H	N	O	S	Cl
450.0794	450.0810	-1.6	-3.6	11.5	C21 H22 N3 O2 S Cl2	371.2	0.0	21	22	3	2	1	2

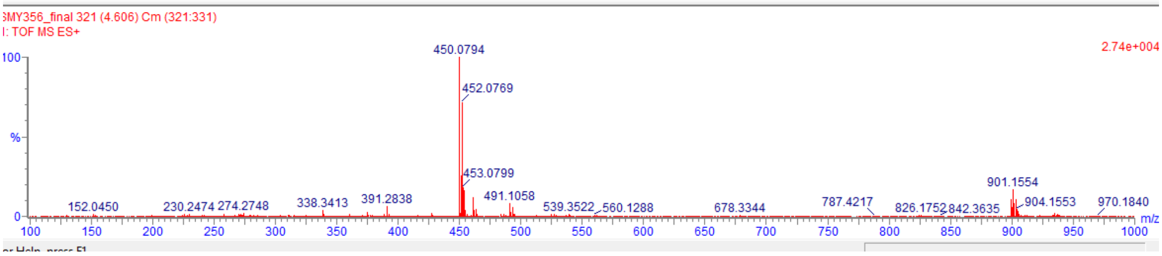

Figure S18. ^{1}H-NMR, ^{13}C-NMR and HRMS spectrums of 39

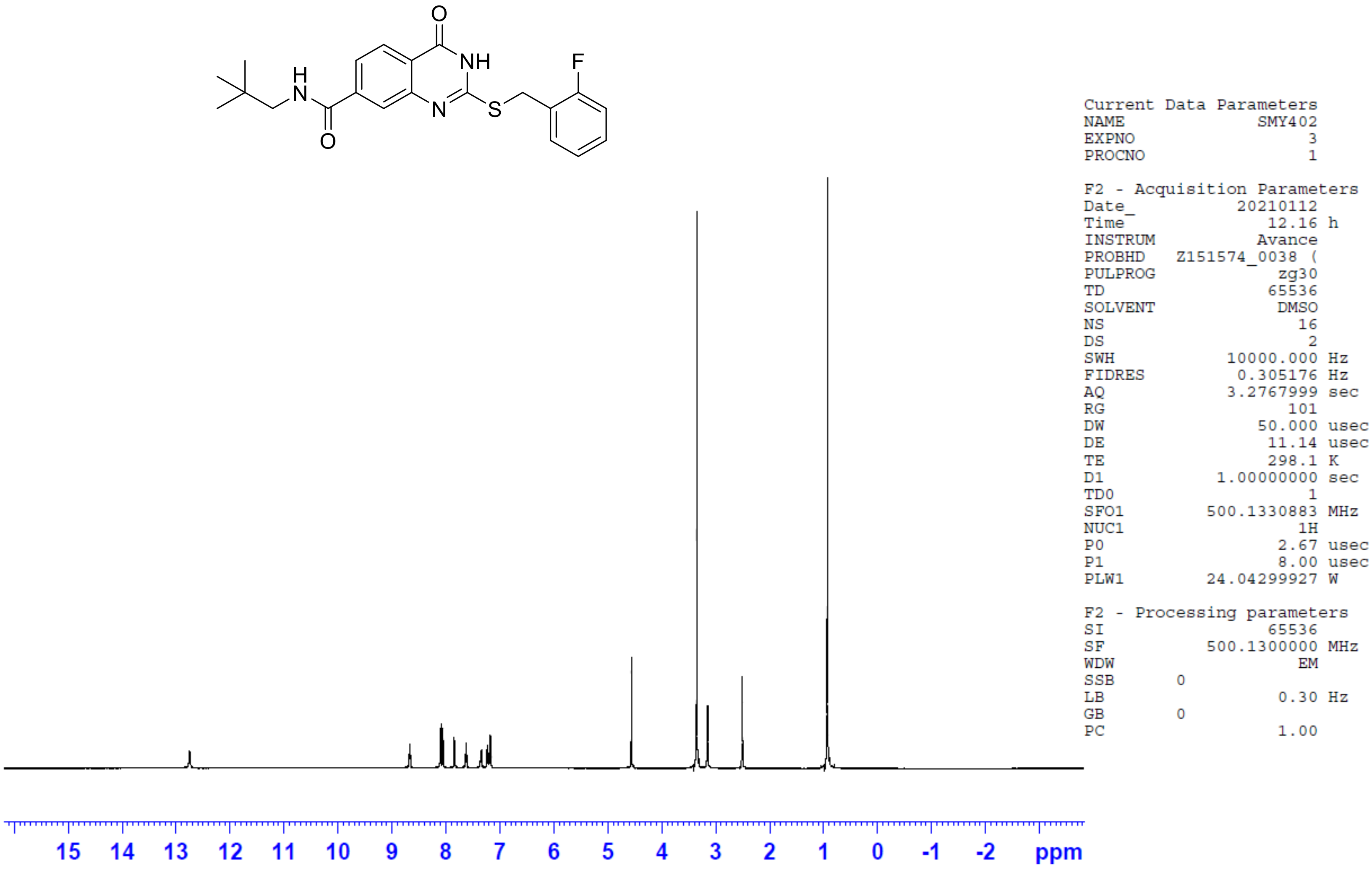

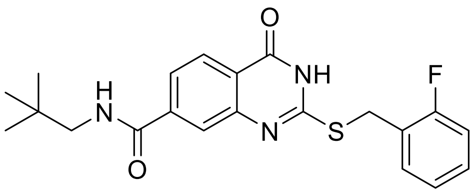

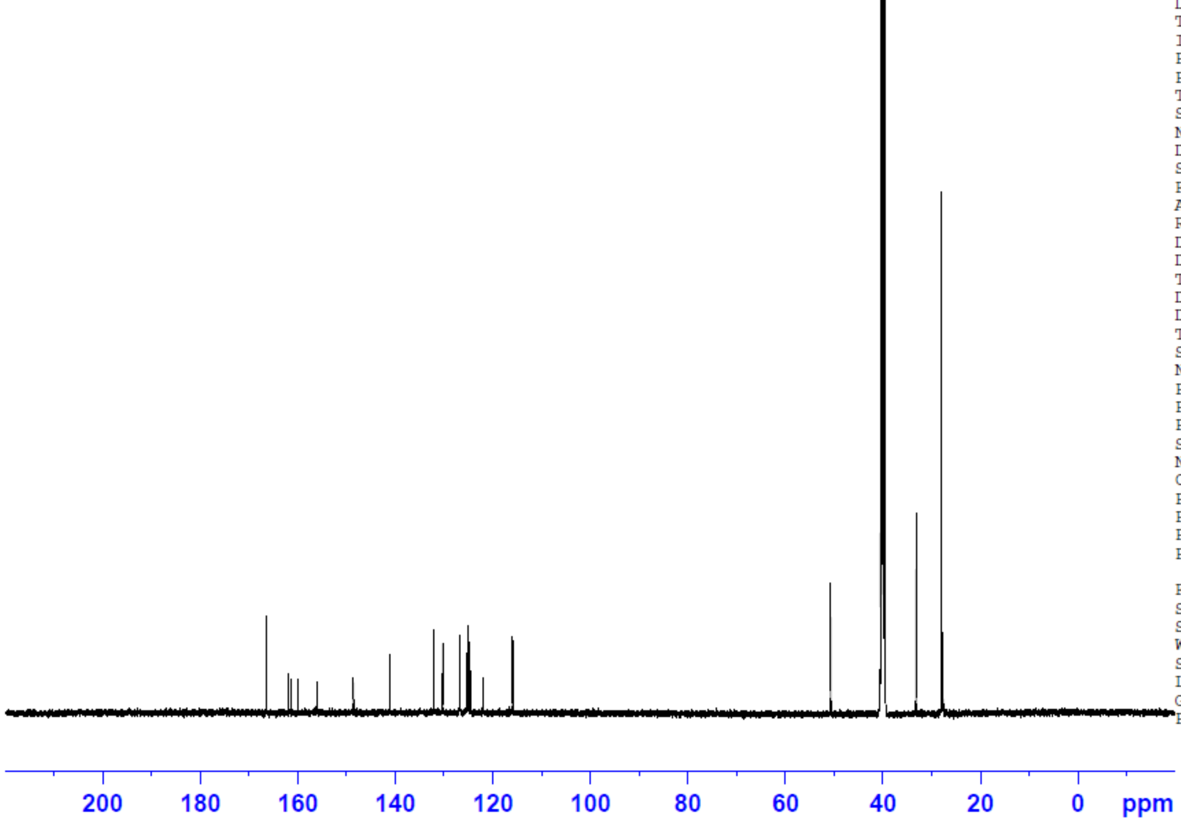


```
Current Data Parameters
NAME              SMY402
EXPNO                  2
PROCNO                 1

F2 - Acquisition Parameters
Date_           20210112
Time               12.10 h
INSTRUM           Avance
PROBHD   Z151574_0038 (
PULPROG          zgpg30
TD                 65536
SOLVENT             DMSO
NS                  1300
DS                     4
SWH            30120.482 Hz
FIDRES          0.919204 Hz
AQ             1.0878977 sec
RG                   101
DW                16.600 usec
DE                  6.50 usec
TE                 299.0 K
D1            2.00000000 sec
D11           0.03000000 sec
TD0                    1
SFO1         125.7703643 MHz
NUC1                 13C
P0                  3.33 usec
P1                 10.00 usec
PLW1         85.18099976 W
SFO2         500.1320005 MHz
NUC2                  1H
CPDPRG[2         waltz65
PCPD2              80.00 usec
PLW2         24.04299927 W
PLW12         0.24043000 W
PLW13         0.12093000 W

F2 - Processing parameters
SI                 32768
SF           125.7577885 MHz
WDW                   EM
SSB      0
LB                  1.00 Hz
GB       0
PC                  1.40
```

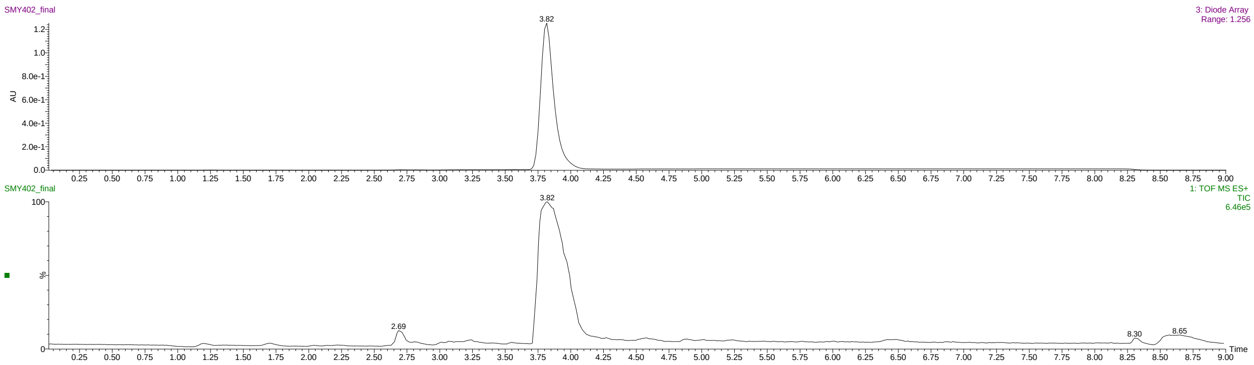

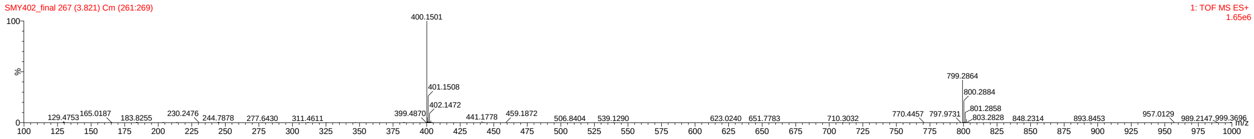

Single Mass Analysis
Tolerance = 5.0 PPM / DBE: min = -1.5, max = 50.0
Element prediction: Off
Number of isotope peaks used for i-FIT = 3
Monoisotopic Mass, Even Electron Ions
35 formula(e) evaluated with 1 results within limits (up to 50 closest results for each mass)
Elements Used:

Mass	Calc. Mass	mDa	PPM	DBE	Formula	i-FIT	i-FIT (Norm)	C	H	N	O	F	S
400.1501	400.1495	0.6	1.5	11.5	C21 H23 N3 O2 F S	594.8	0.0	21	23	3	2	1	1

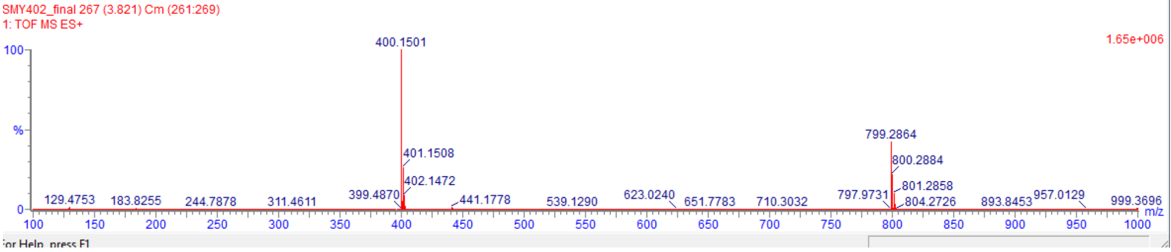

For Help, press F1

Figure S19. ^{1}H-NMR, ^{13}C-NMR and HRMS spectrums of 40

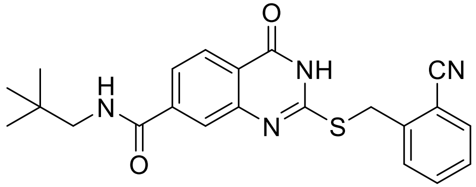

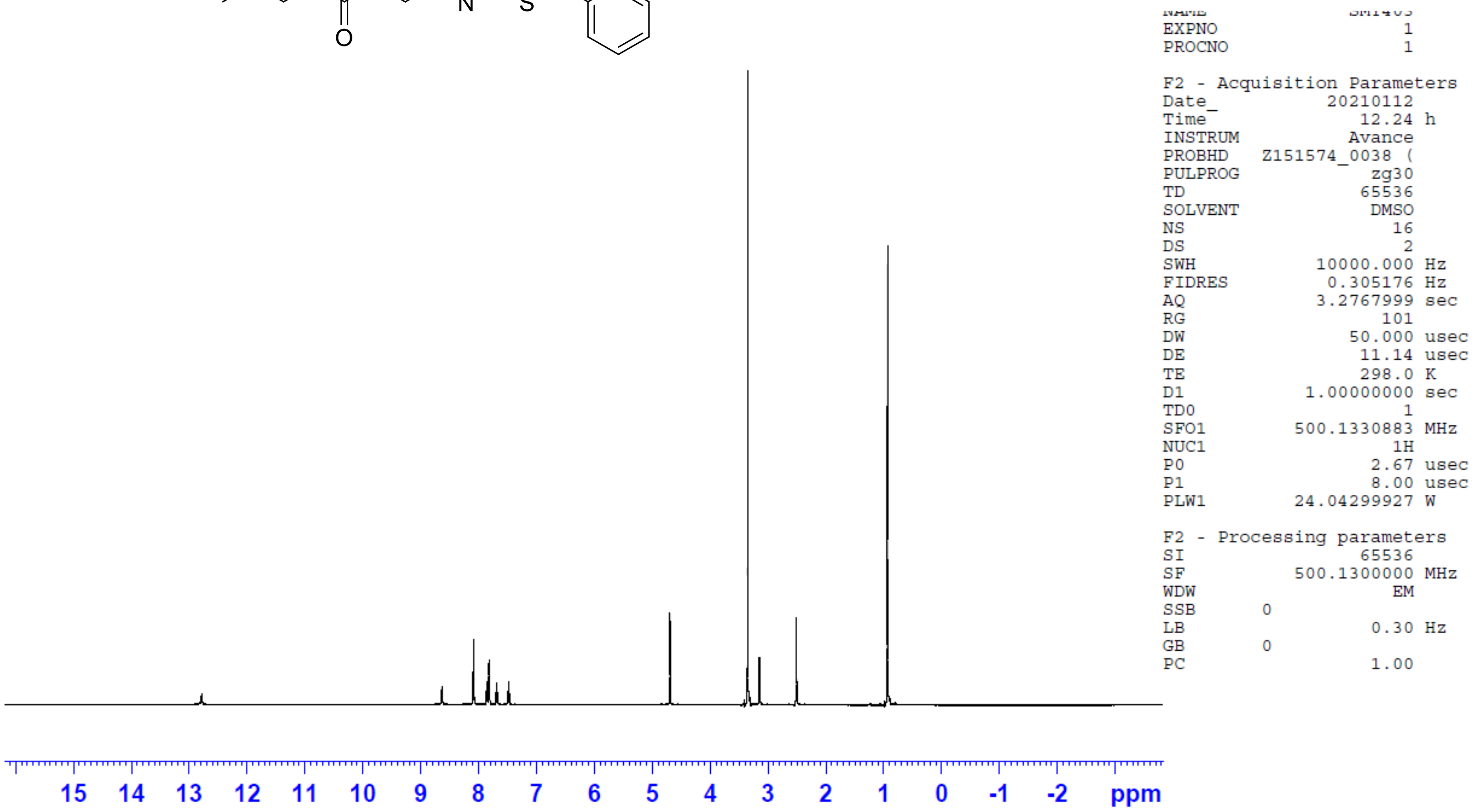

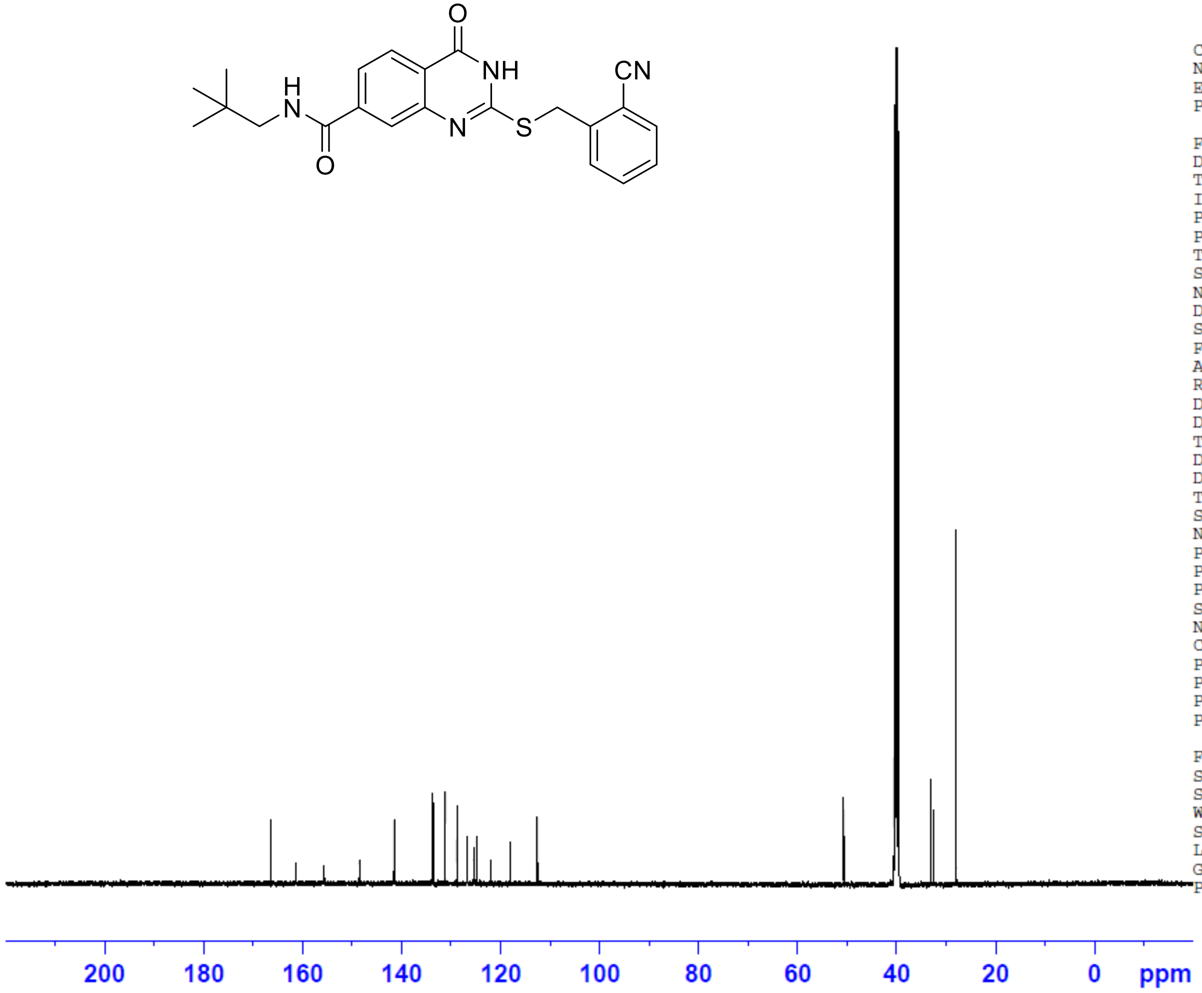

Current Data Parameters
NAME SMY403
EXPNO 2
PROCNO 1

F2 - Acquisition Parameters
Date_ 20210112
Time 13.46 h
INSTRUM Avance
PROBHD Z151574_0038 (
PULPROG zgpg30
TD 65536
SOLVENT DMSO
NS 1500
DS 4
SWH 30120.482 Hz
FIDRES 0.919204 Hz
AQ 1.0878977 sec
RG 101
DW 16.600 usec
DE 6.50 usec
TE 298.8 K
D1 2.00000000 sec
D11 0.03000000 sec
TD0 1
SFO1 125.7703643 MHz
NUC1 13C
P0 3.33 usec
P1 10.00 usec
PLW1 85.18099976 W
SFO2 500.1320005 MHz
NUC2 1H
CPDPRG[2 waltz65
PCPD2 80.00 usec
PLW2 24.04299927 W
PLW12 0.24043000 W
PLW13 0.12093000 W

F2 - Processing parameters
SI 32768
SF 125.7577885 MHz
WDW EM
SSB 0
LB 1.00 Hz
GB 0
PC 1.40

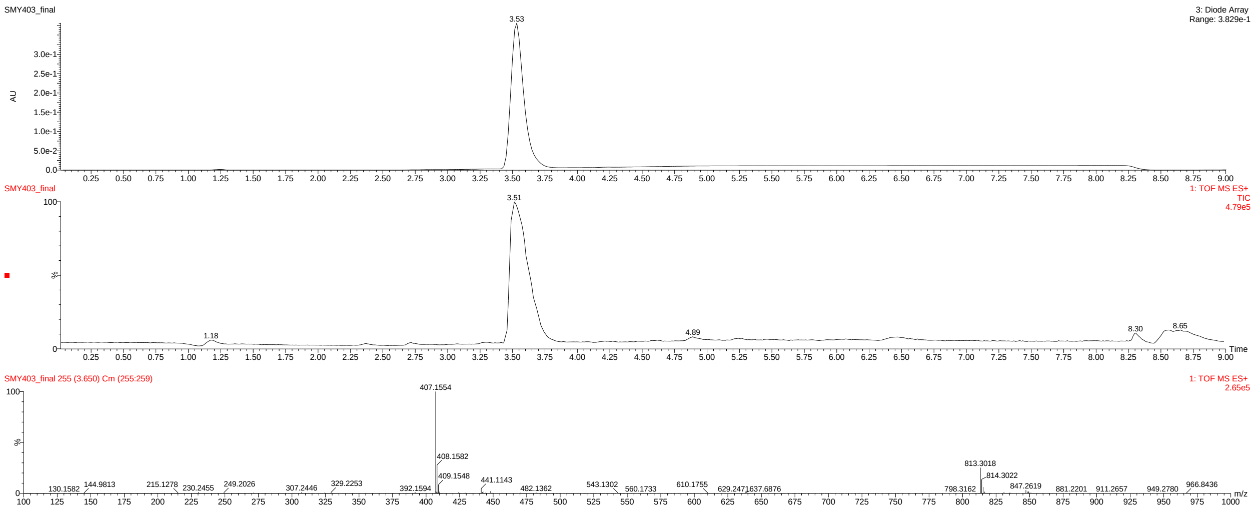

Single Mass Analysis
Tolerance = 5.0 PPM / DBE: min = -1.5, max = 50.0
Element prediction: Off
Number of isotope peaks used for i-FIT = 3
Monoisotopic Mass, Even Electron Ions
19 formula(e) evaluated with 1 results within limits (up to 50 closest results for each mass)
Elements Used:

Mass	Calc. Mass	mDa	PPM	DBE	Formula	i-FIT	i-FIT (Norm)	C	H	N	O	S
407.1554	407.1542	1.2	2.9	13.5	C22 H23 N4 O2 S	416.0	0.0	22	23	4	2	1

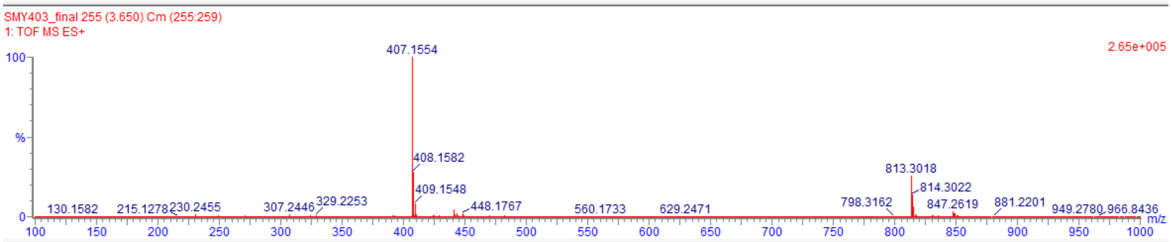

Figure S20. ^{1}H-NMR, ^{13}C-NMR and HRMS spectrums of 41

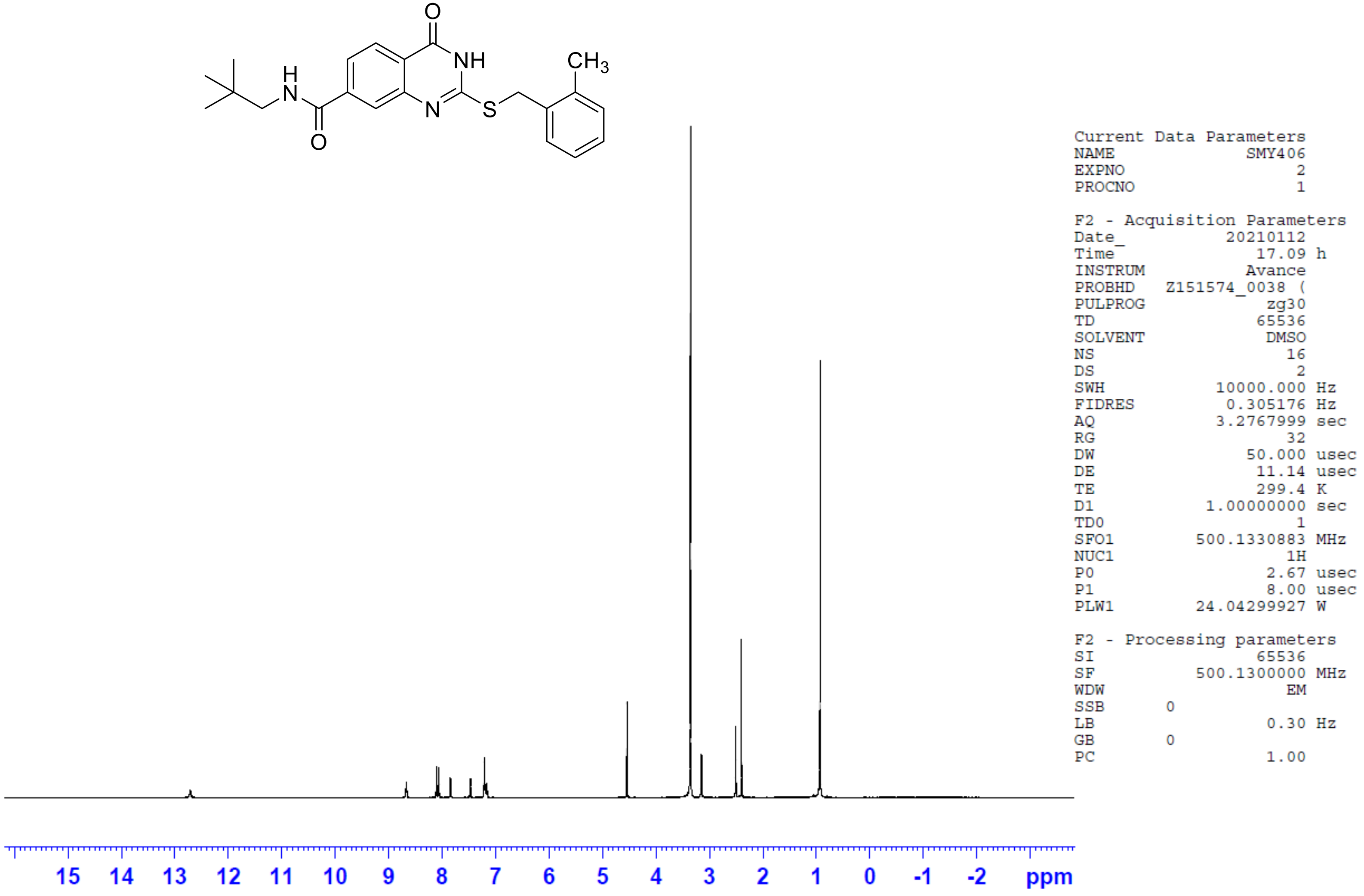

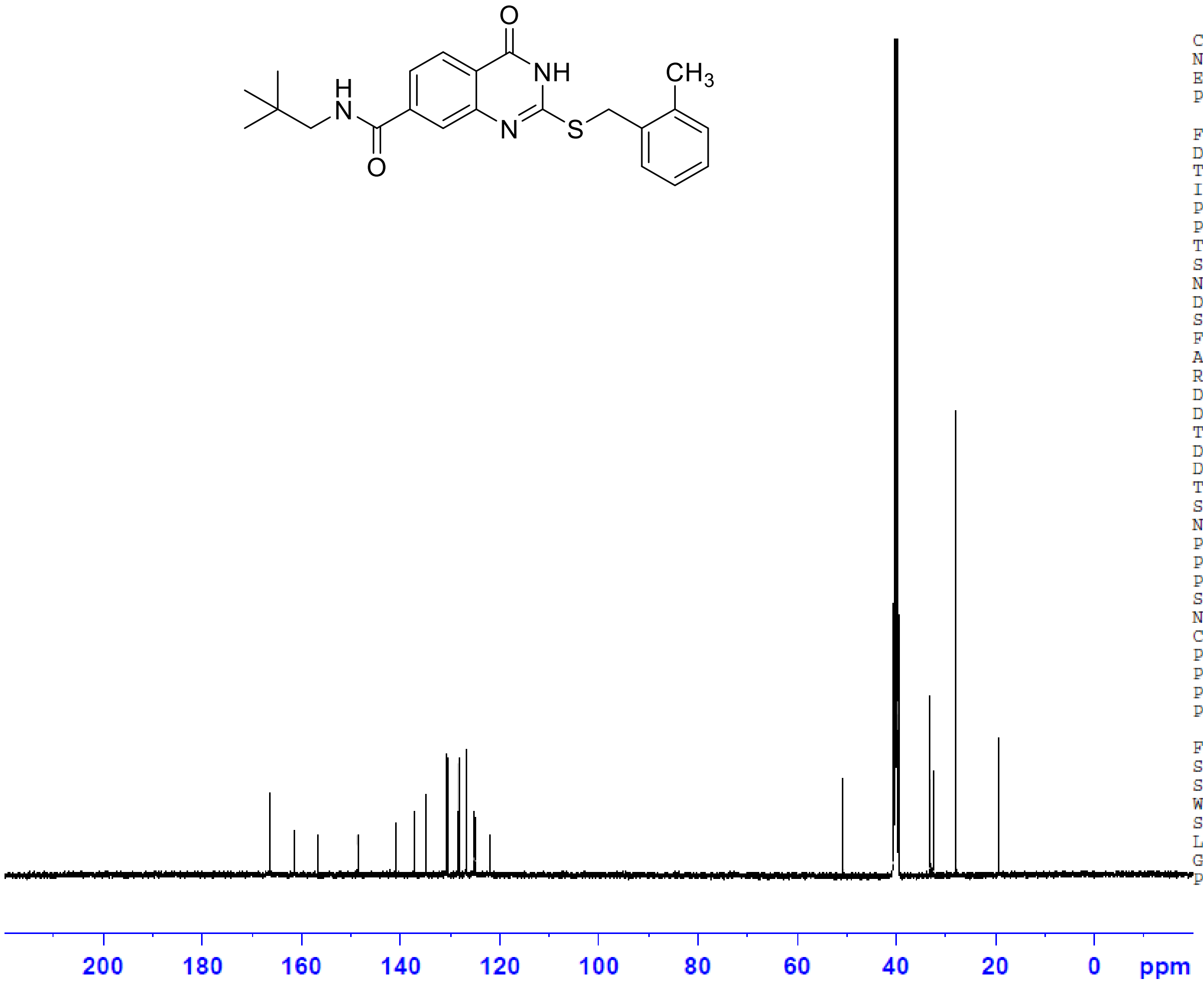


```
Current Data Parameters
NAME              SMY406
EXPNO                  3
PROCNO                 1

F2 - Acquisition Parameters
Date_           20210112
Time               18.20 h
INSTRUM           Avance
PROBHD   Z151574_0038 (
PULPROG          zgpg30
TD                 65536
SOLVENT             DMSO
NS                  1270
DS                     4
SWH            30120.482 Hz
FIDRES          0.919204 Hz
AQ             1.0878977 sec
RG                   101
DW                16.600 usec
DE                  6.50 usec
TE                 300.7 K
D1            2.00000000 sec
D11           0.03000000 sec
TD0                    1
SFO1         125.7703643 MHz
NUC1                 13C
P0                  3.33 usec
P1                 10.00 usec
PLW1         85.18099976 W
SFO2         500.1320005 MHz
NUC2                  1H
CPDPRG[2         waltz65
PCPD2              80.00 usec
PLW2         24.04299927 W
PLW12         0.24043000 W
PLW13         0.12093000 W

F2 - Processing parameters
SI                 32768
SF           125.7577885 MHz
WDW                   EM
SSB       0
LB                  1.00 Hz
GB        0
PC                  1.40
```

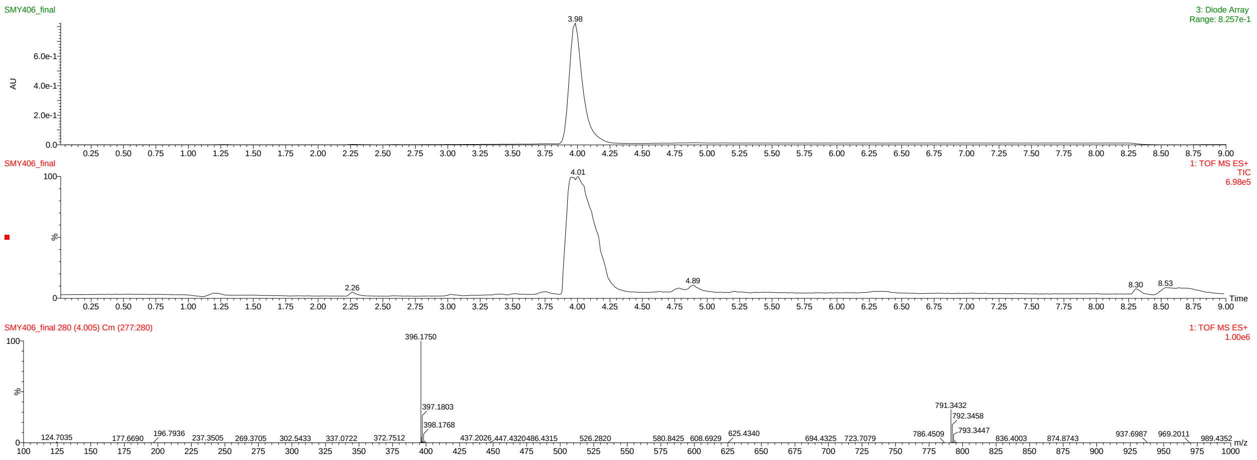

Single Mass Analysis
Tolerance = 5.0 PPM / DBE: min = -1.5, max = 50.0
Element prediction: Off
Number of isotope peaks used for i-FIT = 3
Monoisotopic Mass, Even Electron Ions
27 formula(e) evaluated with 1 results within limits (up to 50 closest results for each mass)
Elements Used:

Mass	Calc. Mass	mDa	PPM	DBE	Formula	i-FIT	i-FIT (Norm)	C	H	N	O	S
396.1750	396.1746	0.4	1.0	11.5	C22 H26 N3 O2 S	496.7	0.0	22	26	3	2	1

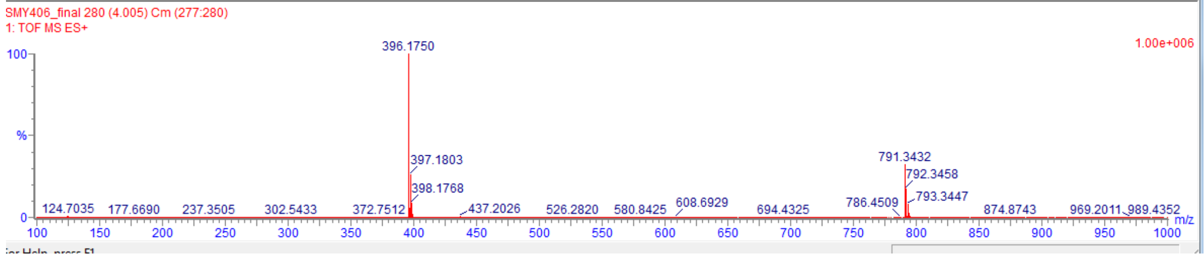

Figure S21. ^{1}H-NMR, ^{13}C-NMR and HRMS spectrums of 42

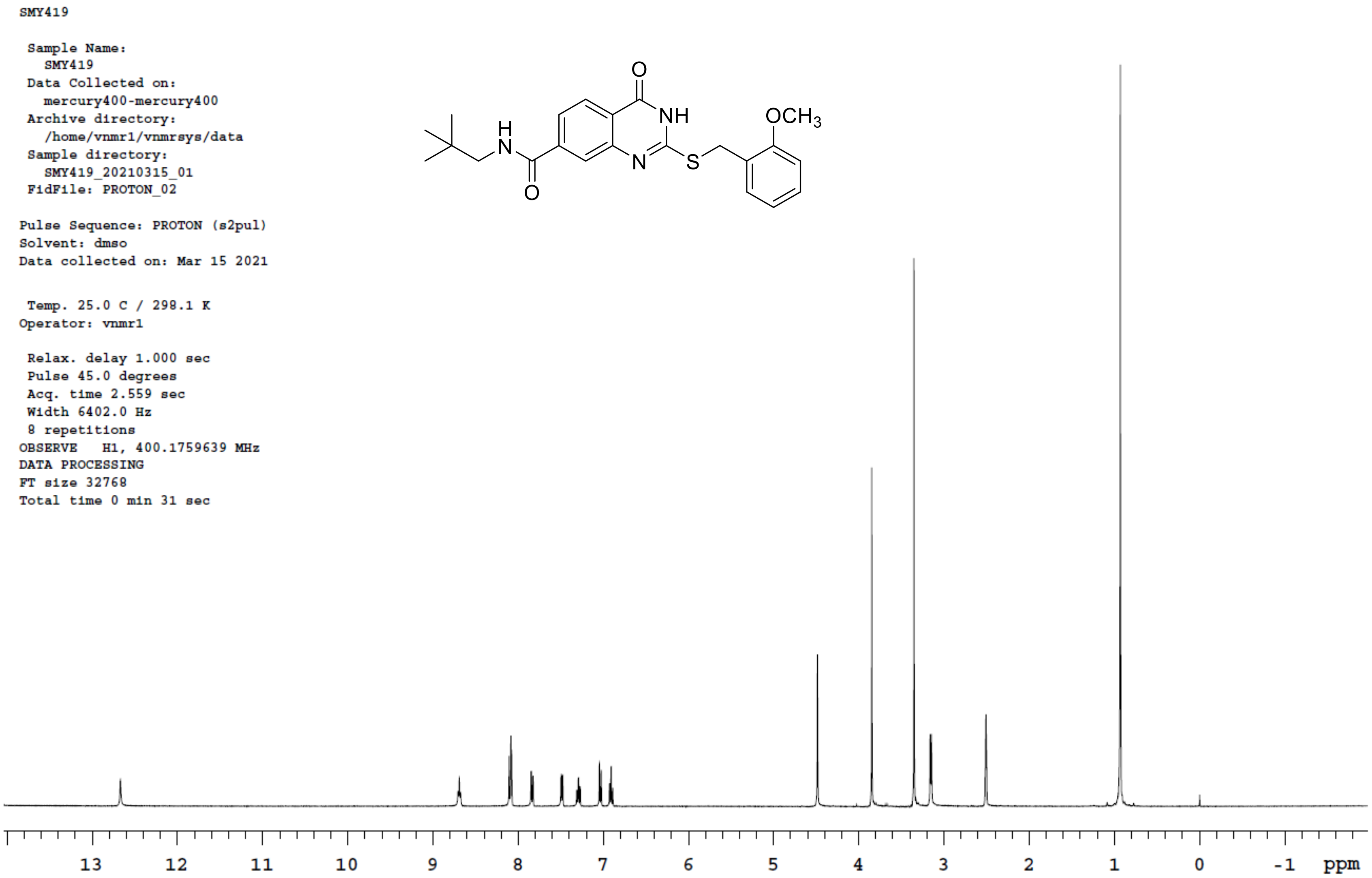

SMY419

Sample Name:
SMY419
Data Collected on:
mercury400-mercury400
Archive directory:
/home/vnmr1/vnmrsys/data
Sample directory:
SMY419_20210315_01
FidFile: CARBON_01

Pulse Sequence: CARBON (s2pul)
Solvent: dmso
Data collected on: Mar 15 2021

Temp. 25.0 C / 298.1 K
Operator: vnmr1

Relax. delay 1.000 sec
Pulse 45.0 degrees
Acq. time 1.550 sec
Width 21141.6 Hz
1512 repetitions
OBSERVE C13, 100.6243742 MHz
DECOUPLE H1, 400.1779555 MHz
Power 38 dB
continuously on
WALTZ-16 modulated
DATA PROCESSING
Line broadening 0.5 Hz
FT size 65536
Total time 1 hr, 6 min

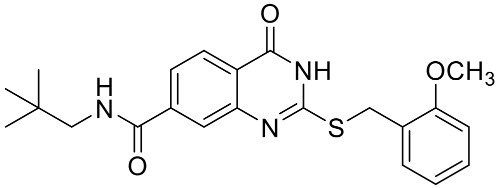

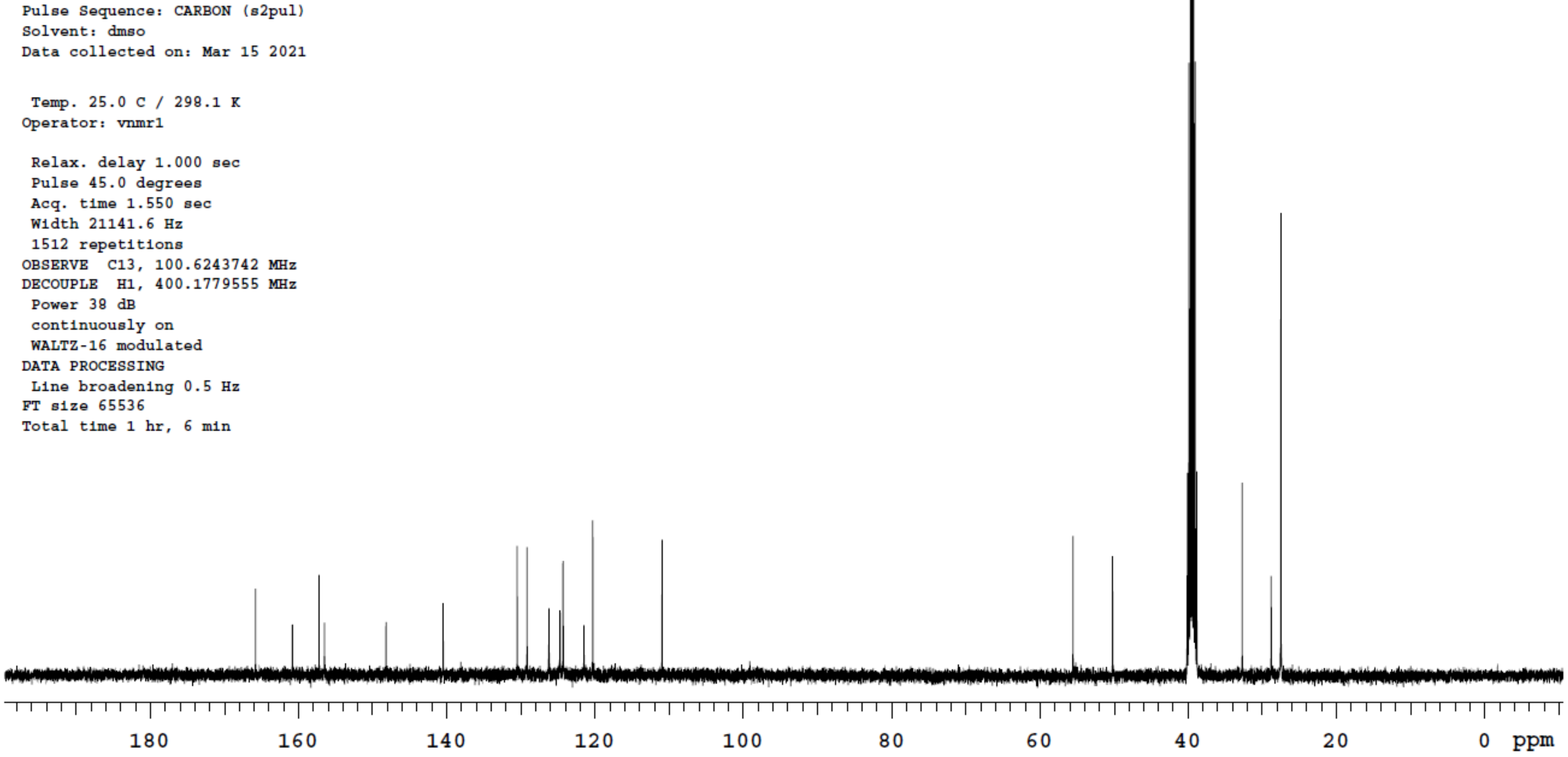

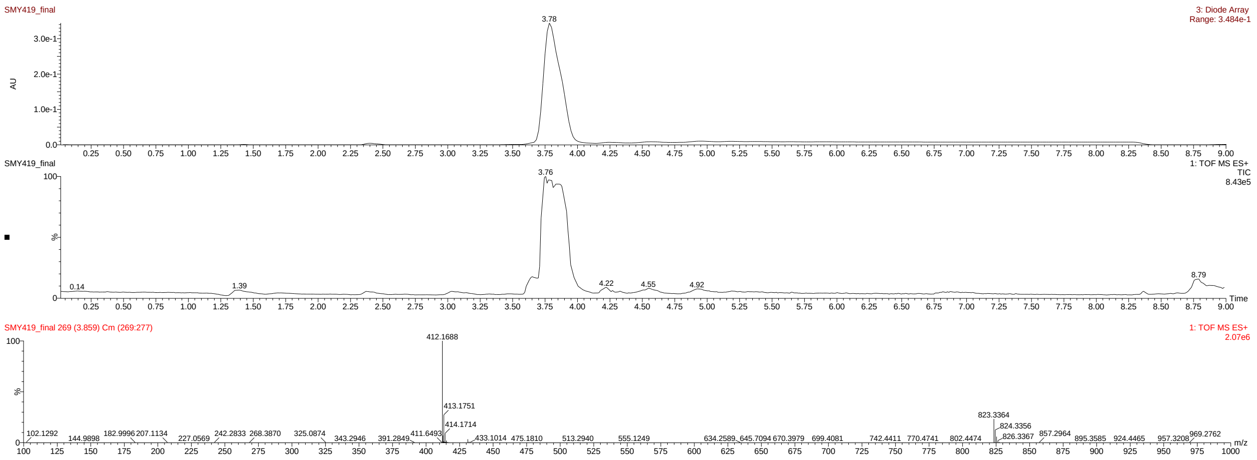

Single Mass Analysis
Tolerance = 5.0 PPM / DBE: min = -1.5, max = 50.0
Element prediction: Off
Number of isotope peaks used for i-FIT = 3
Monoisotopic Mass, Even Electron Ions
18 formula(e) evaluated with 1 results within limits (up to 50 closest results for each mass)
Elements Used:

Mass	Calc. Mass	mDa	PPM	DBE	Formula	i-FIT	i-FIT (Norm)	C	H	N	O	S
412.1688	412.1695	-0.7	-1.7	11.5	C22 H26 N3 O3 S	627.1	0.0	22	26	3	3	1

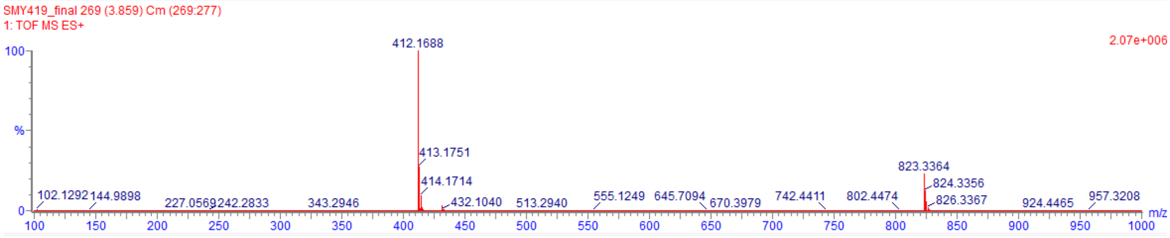

Figure S22. ^{1}H-NMR, ^{13}C-NMR and HRMS spectrums of 43

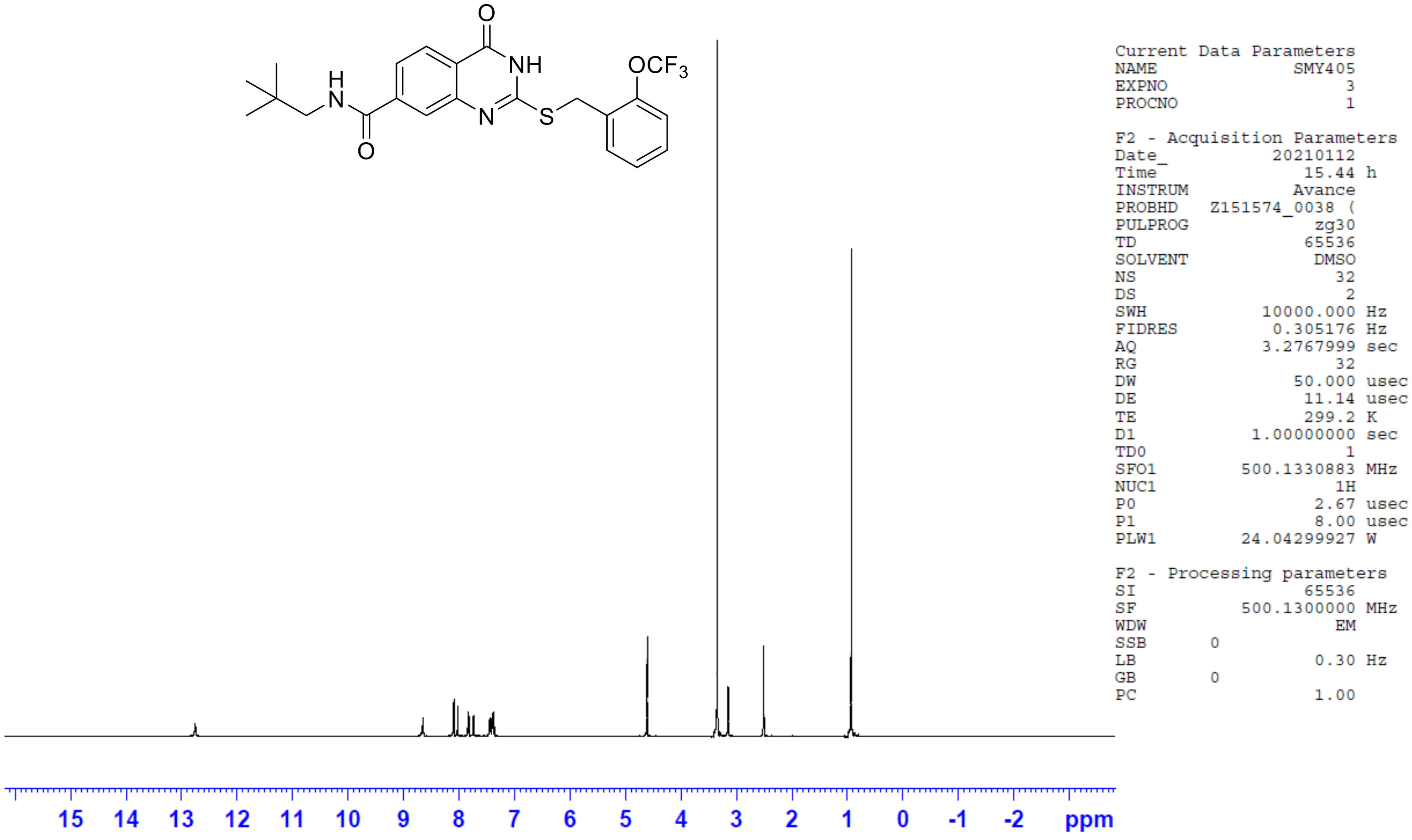


```
Current Data Parameters
NAME              SMY405
EXPNO                  3
PROCNO                 1

F2 - Acquisition Parameters
Date_           20210112
Time               15.44 h
INSTRUM           Avance
PROBHD    Z151574_0038 (
PULPROG             zg30
TD                 65536
SOLVENT             DMSO
NS                    32
DS                     2
SWH            10000.000 Hz
FIDRES          0.305176 Hz
AQ             3.2767999 sec
RG                    32
DW                50.000 usec
DE                 11.14 usec
TE                 299.2 K
D1            1.00000000 sec
TD0                    1
SFO1         500.1330883 MHz
NUC1                  1H
P0                  2.67 usec
P1                  8.00 usec
PLW1         24.04299927 W

F2 - Processing parameters
SI                 65536
SF           500.1300000 MHz
WDW                   EM
SSB      0
LB                  0.30 Hz
GB       0
PC                  1.00
```

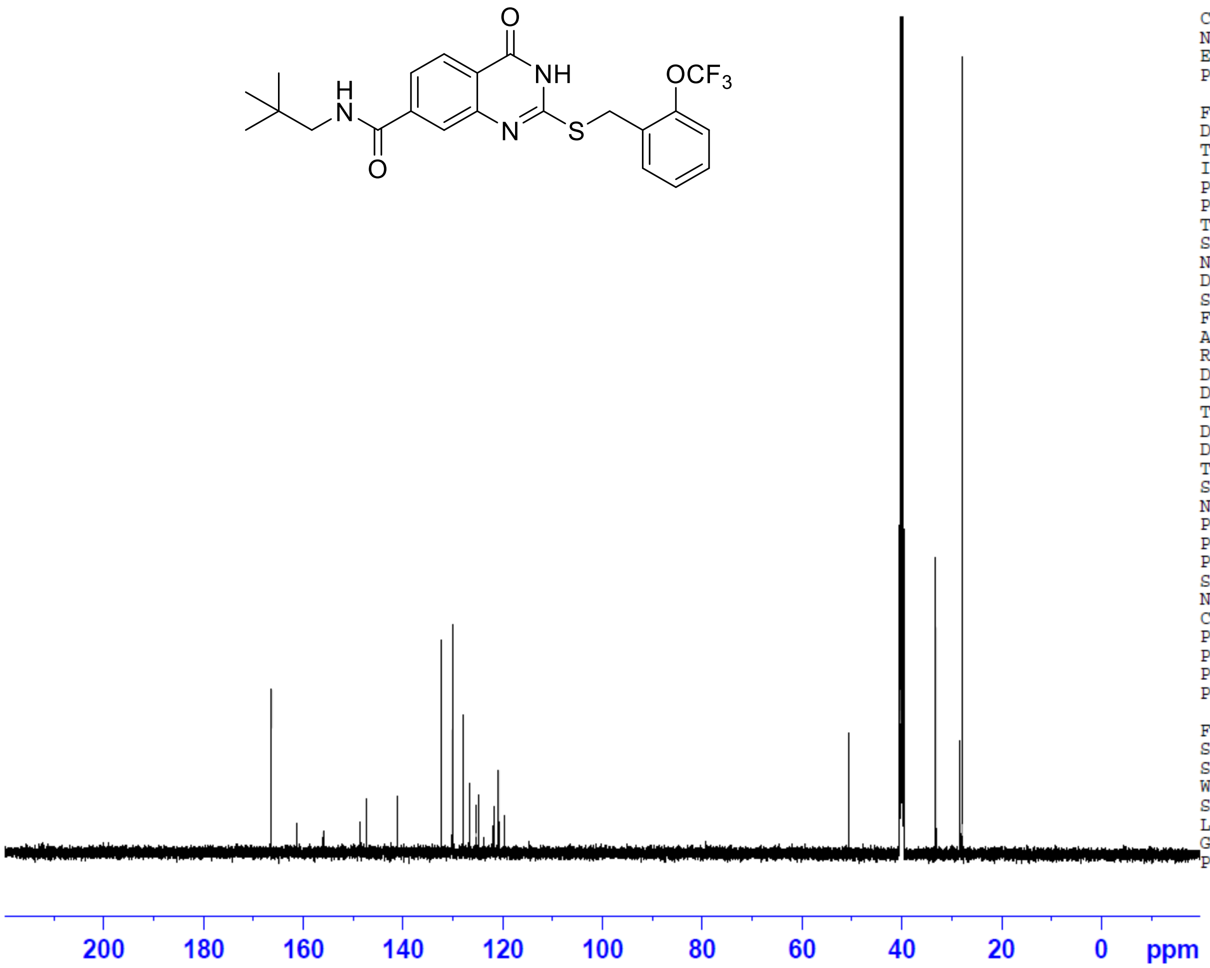

Current Data Parameters
NAME SMY405
EXPNO 1
PROCNO 1

F2 - Acquisition Parameters
Date_ 20210112
Time 15.37 h
INSTRUM Avance
PROBHD Z151574_0038 (
PULPROG zgpg30
TD 65536
SOLVENT DMSO
NS 1600
DS 4
SWH 30120.482 Hz
FIDRES 0.919204 Hz
AQ 1.0878977 sec
RG 101
DW 16.600 usec
DE 6.50 usec
TE 300.0 K
D1 2.00000000 sec
D11 0.03000000 sec
TD0 1
SFO1 125.7703643 MHz
NUC1 13C
P0 3.33 usec
P1 10.00 usec
PLW1 85.18099976 W
SFO2 500.1320005 MHz
NUC2 1H
CPDPRG[2 waltz65
PCPD2 80.00 usec
PLW2 24.04299927 W
PLW12 0.24043000 W
PLW13 0.12093000 W

F2 - Processing parameters
SI 32768
SF 125.7577885 MHz
WDW no
SSB 0
LB 0 Hz
GB 0
PC 1.40

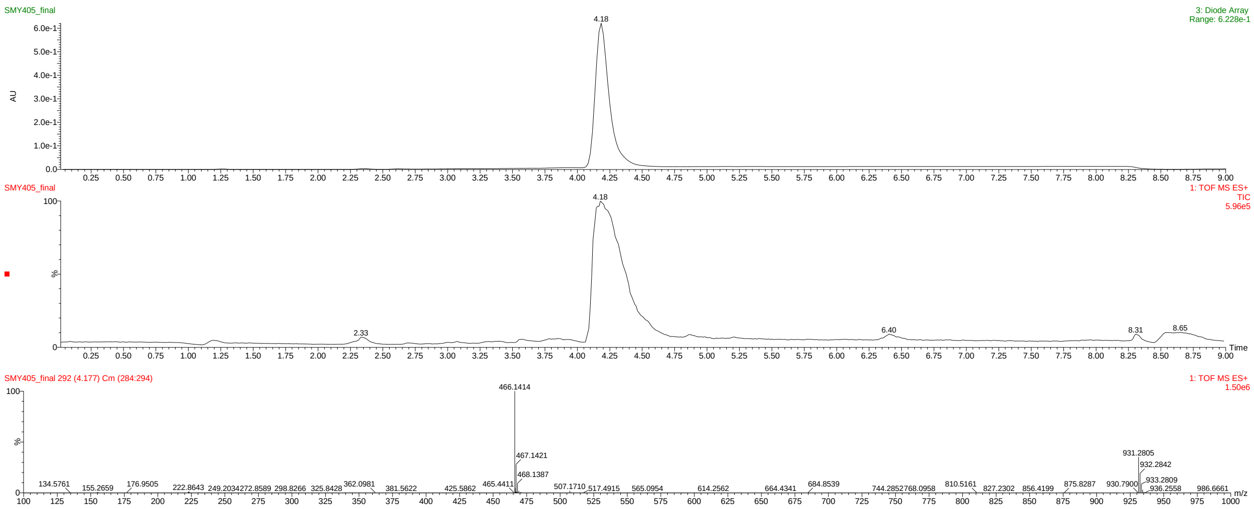

Single Mass Analysis
Tolerance = 5.0 PPM / DBE: min = -1.5, max = 50.0
Element prediction: Off
Number of isotope peaks used for i-FIT = 3
Monoisotopic Mass, Even Electron Ions
77 formula(e) evaluated with 1 results within limits (up to 50 closest results for each mass)
Elements Used:

Mass	Calc. Mass	mDa	PPM	DBE	Formula	i-FIT	i-FIT (Norm)	C	H	N	O	F	S
466.1414	466.1412	0.2	0.4	11.5	C22 H23 N3 O3 F3 S	522.1	0.0	22	23	3	3	3	1

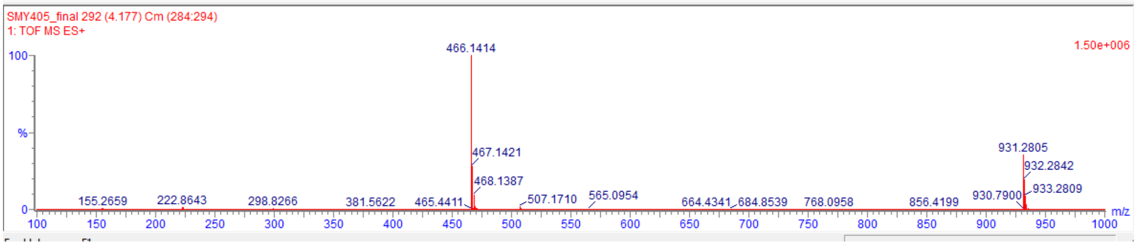

Figure S23. ^{1}H-NMR, ^{13}C-NMR and HRMS spectrums of 44

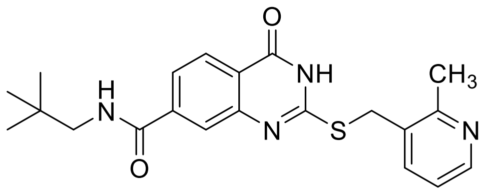

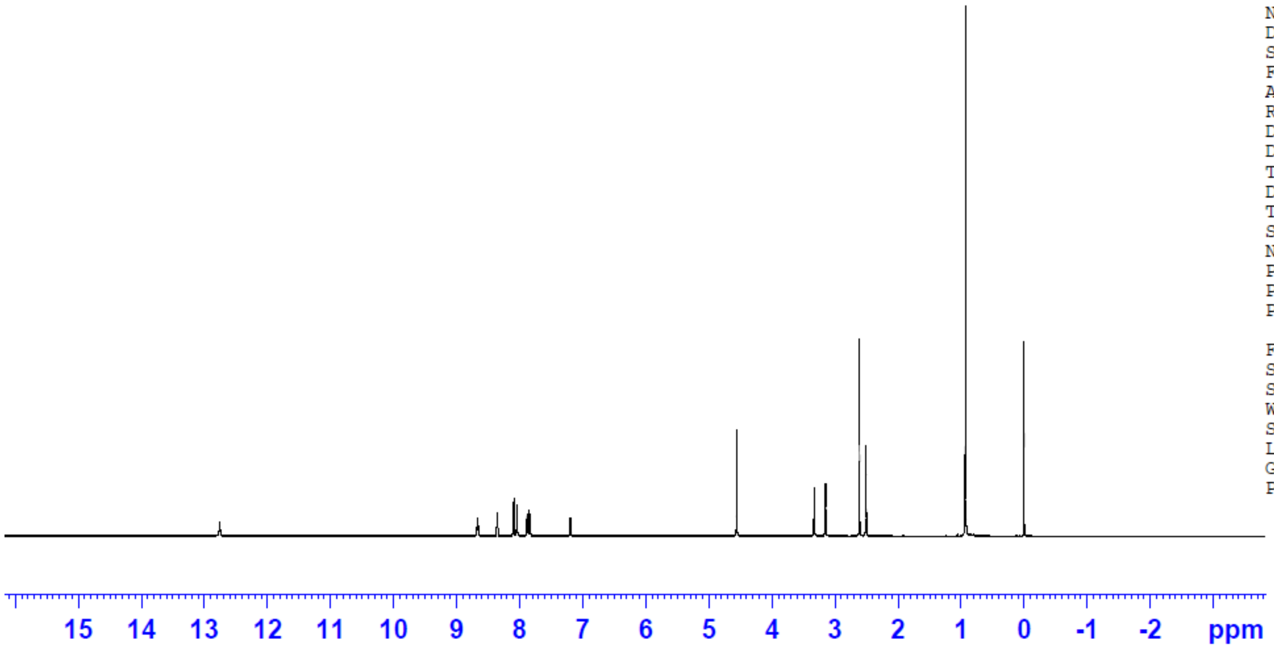

Current Data Parameters
NAME SMY407
EXPNO 1
PROCNO 1

F2 - Acquisition Parameters
Date_ 20210113
Time 10.30 h
INSTRUM Avance
PROBHD Z151574_0038 (
PULPROG zg30
TD 65536
SOLVENT DMSO
NS 16
DS 2
SWH 10000.000 Hz
FIDRES 0.305176 Hz
AQ 3.2767999 sec
RG 32
DW 50.000 usec
DE 11.14 usec
TE 298.5 K
D1 1.00000000 sec
TD0 1
SFO1 500.1330883 MHz
NUC1 1H
P0 2.67 usec
P1 8.00 usec
PLW1 24.04299927 W

F2 - Processing parameters
SI 65536
SF 500.1300000 MHz
WDW EM
SSB 0
LB 0.30 Hz
GB 0
PC 1.00

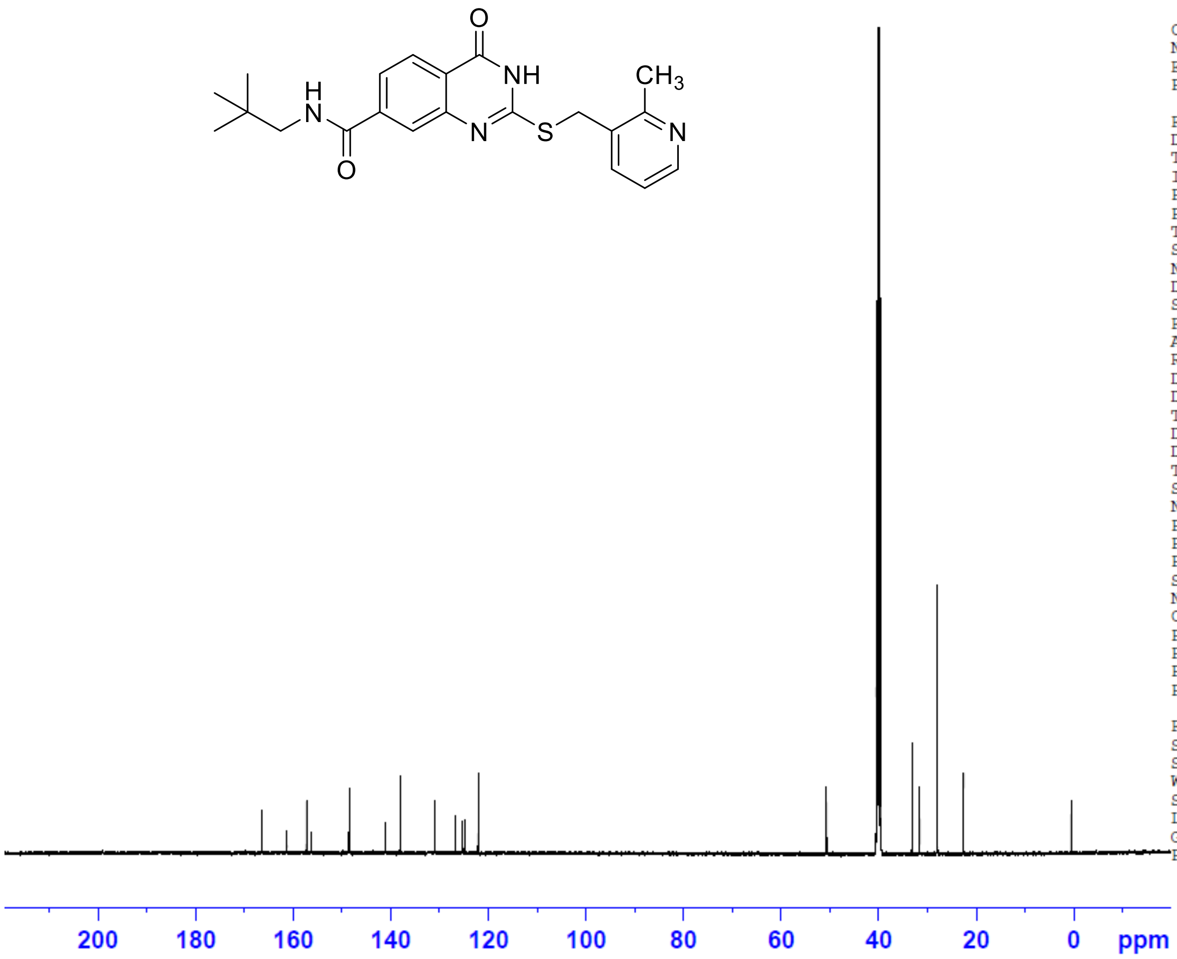


```
Current Data Parameters
NAME              SMY407
EXPNO                  2
PROCNO                 1

F2 - Acquisition Parameters
Date_           20210113
Time               12.18 h
INSTRUM           Avance
PROBHD   Z151574_0038 (
PULPROG          zgpg30
TD                65536
SOLVENT             DMSO
NS                  2000
DS                     4
SWH            30120.482 Hz
FIDRES          0.919204 Hz
AQ             1.0878977 sec
RG                   101
DW                16.600 usec
DE                  6.50 usec
TE                 299.0 K
D1            2.00000000 sec
D11           0.03000000 sec
TD0                    1
SFO1         125.7703643 MHz
NUC1                 13C
P0                  3.33 usec
P1                 10.00 usec
PLW1         85.18099976 W
SFO2         500.1320005 MHz
NUC2                  1H
CPDPRG[2         waltz65
PCPD2              80.00 usec
PLW2         24.04299927 W
PLW12         0.24043000 W
PLW13         0.12093000 W

F2 - Processing parameters
SI                 32768
SF           125.7577885 MHz
WDW                   EM
SSB      0
LB                  1.00 Hz
GB       0
PC                  1.40
```

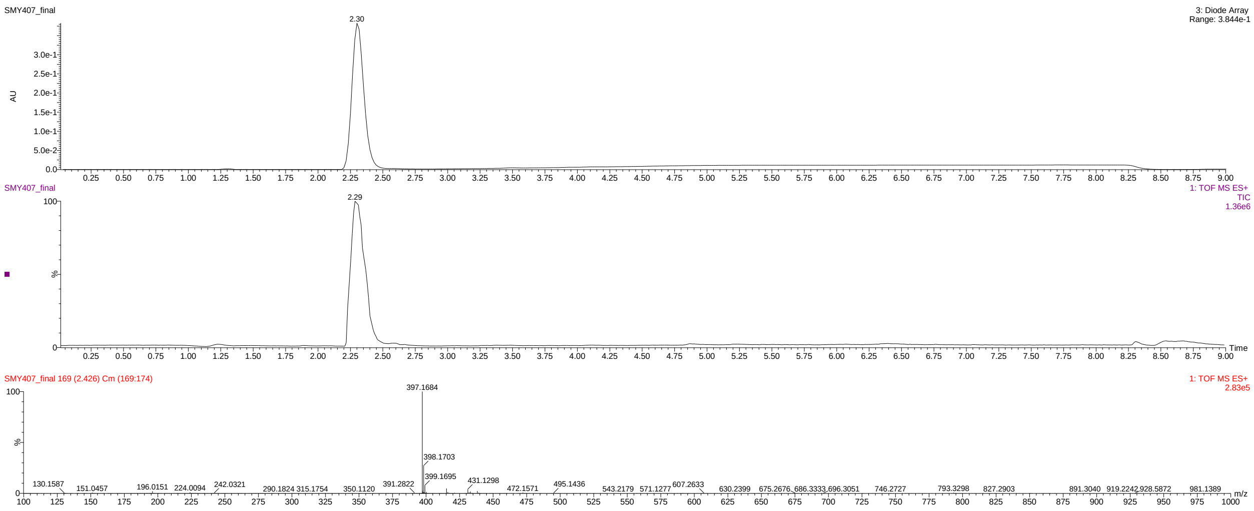

Single Mass Analysis
Tolerance = 5.0 PPM / DBE: min = -1.5, max = 50.0
Element prediction: Off
Number of isotope peaks used for i-FIT = 3
Monoisotopic Mass, Even Electron Ions
26 formula(e) evaluated with 1 results within limits (up to 50 closest results for each mass)
Elements Used:

Mass	Calc. Mass	mDa	PPM	DBE	Formula	i-FIT	i-FIT (Norm)	C	H	N	O	S
397.1684	397.1698	-1.4	-3.5	11.5	C21 H25 N4 O2 S	438.3	0.0	21	25	4	2	1

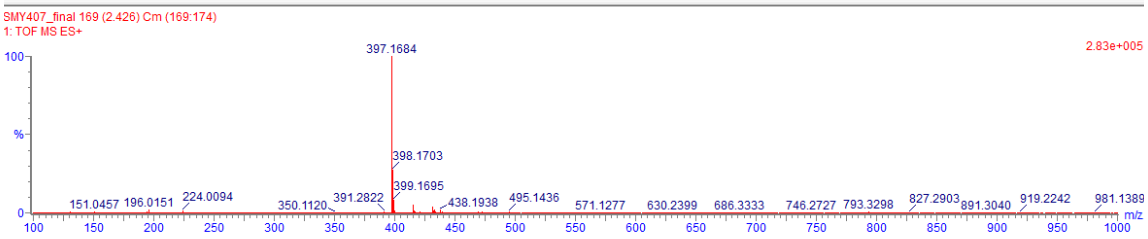

Figure S24. ^{1}H-NMR, ^{13}C-NMR and HRMS spectrums of 45

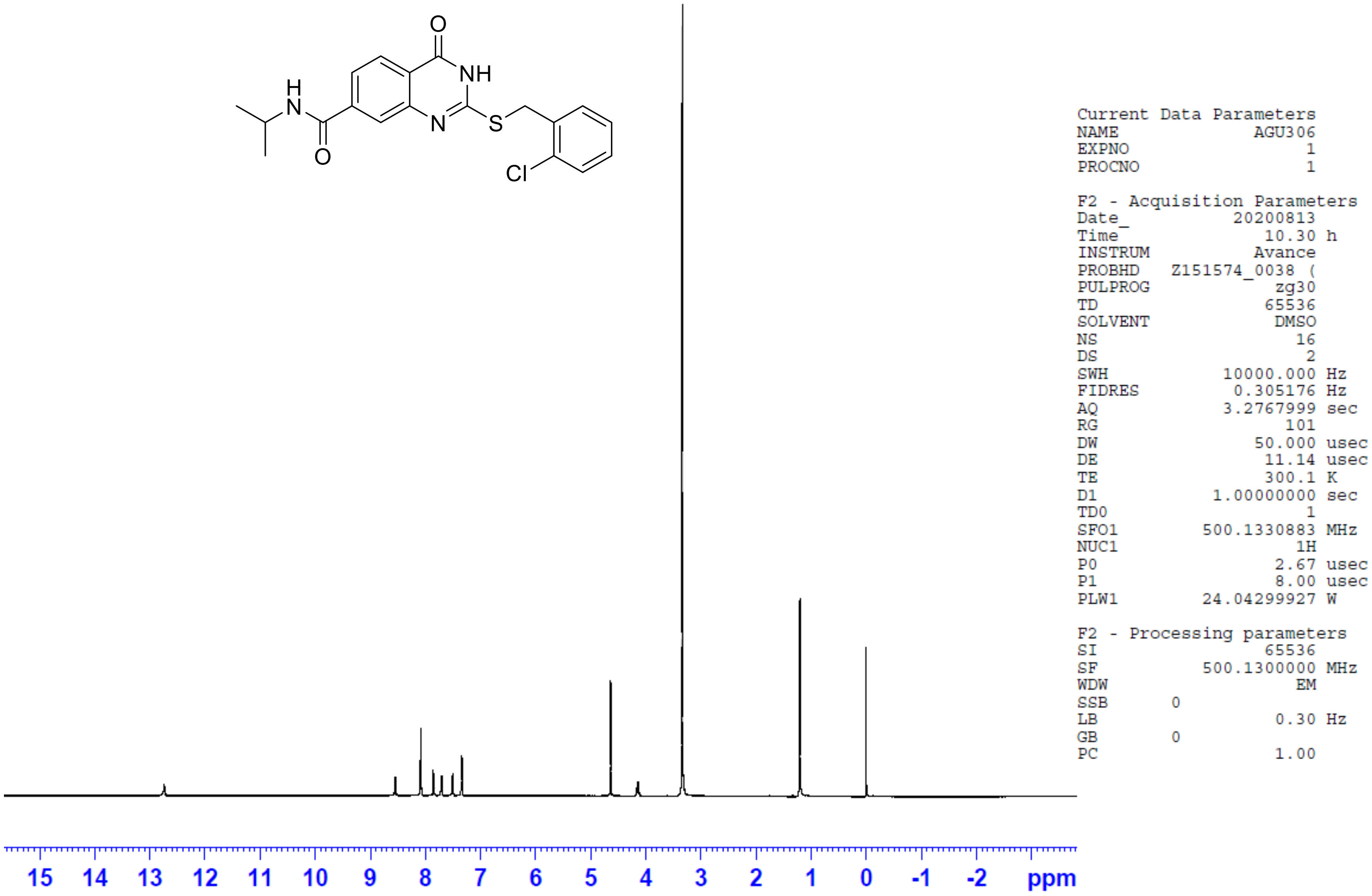


```
Current Data Parameters
NAME              AGU306
EXPNO                  1
PROCNO                 1

F2 - Acquisition Parameters
Date_           20200813
Time               10.30 h
INSTRUM           Avance
PROBHD   Z151574_0038 (
PULPROG             zg30
TD                 65536
SOLVENT             DMSO
NS                    16
DS                     2
SWH            10000.000 Hz
FIDRES          0.305176 Hz
AQ             3.2767999 sec
RG                   101
DW                50.000 usec
DE                 11.14 usec
TE                 300.1 K
D1            1.00000000 sec
TD0                    1
SFO1         500.1330883 MHz
NUC1                  1H
P0                  2.67 usec
P1                  8.00 usec
PLW1         24.04299927 W

F2 - Processing parameters
SI                 65536
SF           500.1300000 MHz
WDW                   EM
SSB      0
LB                  0.30 Hz
GB       0
PC                  1.00
```

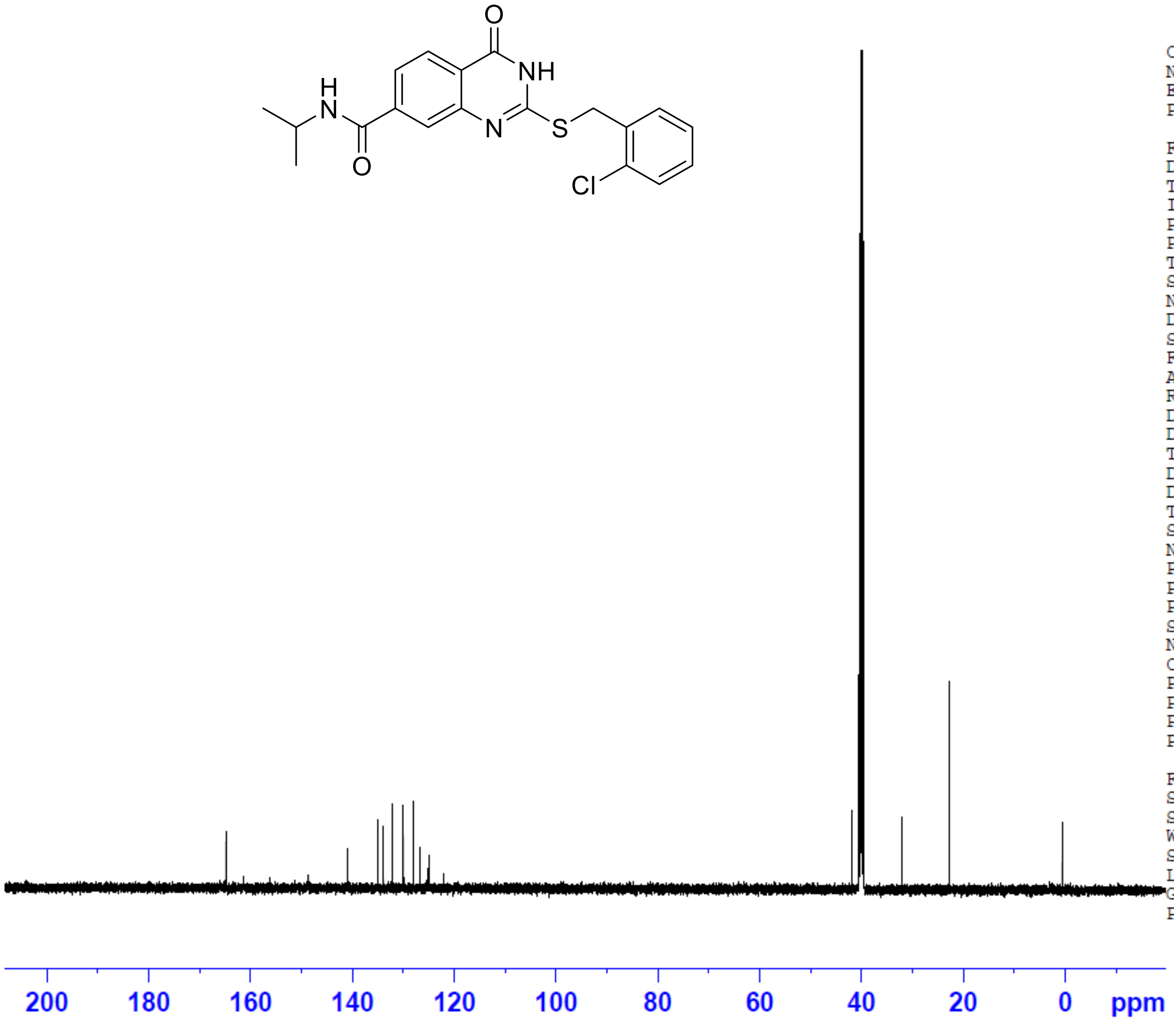


```
Current Data Parameters
NAME            AGU306
EXPNO                2
PROCNO               1

F2 - Acquisition Parameters
Date_          20200813
Time              11.53 h
INSTRUM          Avance
PROBHD   Z151574_0038 (
PULPROG          zgpg30
TD                65536
SOLVENT            DMSO
NS                 1500
DS                    4
SWH           30120.482 Hz
FIDRES         0.919204 Hz
AQ            1.0878977 sec
RG                  101
DW               16.600 usec
DE                 6.50 usec
TE                299.2 K
D1           2.00000000 sec
D11          0.03000000 sec
TD0                   1
SFO1        125.7703643 MHz
NUC1                13C
P0                 3.33 usec
P1                10.00 usec
PLW1        85.18099976 W
SFO2        500.1320005 MHz
NUC2                 1H
CPDPRG[2        waltz65
PCPD2             80.00 usec
PLW2        24.04299927 W
PLW12        0.24043000 W
PLW13        0.12093000 W

F2 - Processing parameters
SI                32768
SF          125.7577885 MHz
WDW                  no
SSB      0
LB       0 Hz
GB       0
PC                 1.40
```

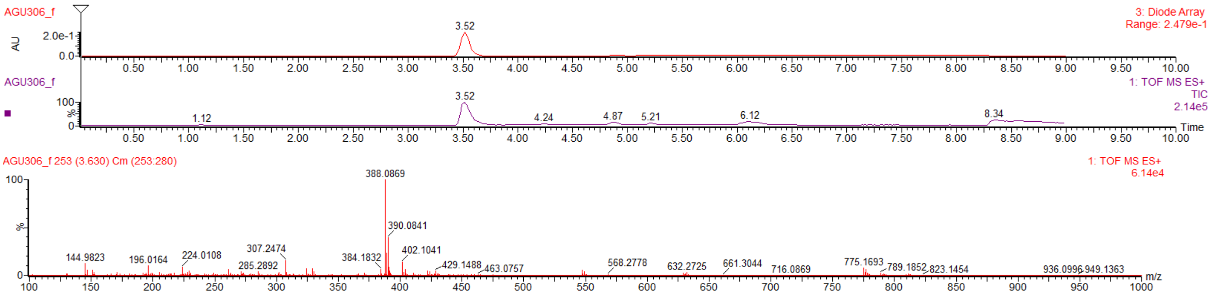

Single Mass Analysis

Tolerance = 5.0 PPM / DBE: min = -1.5, max = 50.0

Element prediction: Off

Number of isotope peaks used for i-FIT = 3

Monoisotopic Mass, Even Electron Ions

133 formula(e) evaluated with 1 results within limits (up to 50 closest results for each mass)

Elements Used:

Mass	Calc. Mass	mDa	PPM	DBE	Formula	i-FIT	i-FIT (Norm)	C	H	N	O	S	Cl
388.0869	388.0887	-1.8	-4.6	11.5	C19 H19 N3 O2 S Cl	427.6	0.0	19	19	3	2	1	1

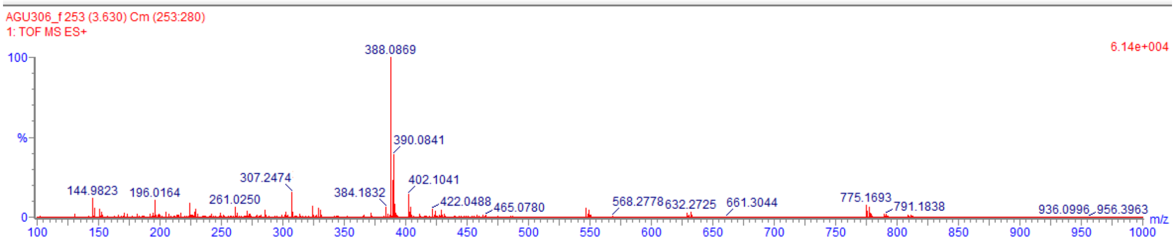

Figure S25. ^{1}H-NMR, ^{13}C-NMR and HRMS spectrums of 46

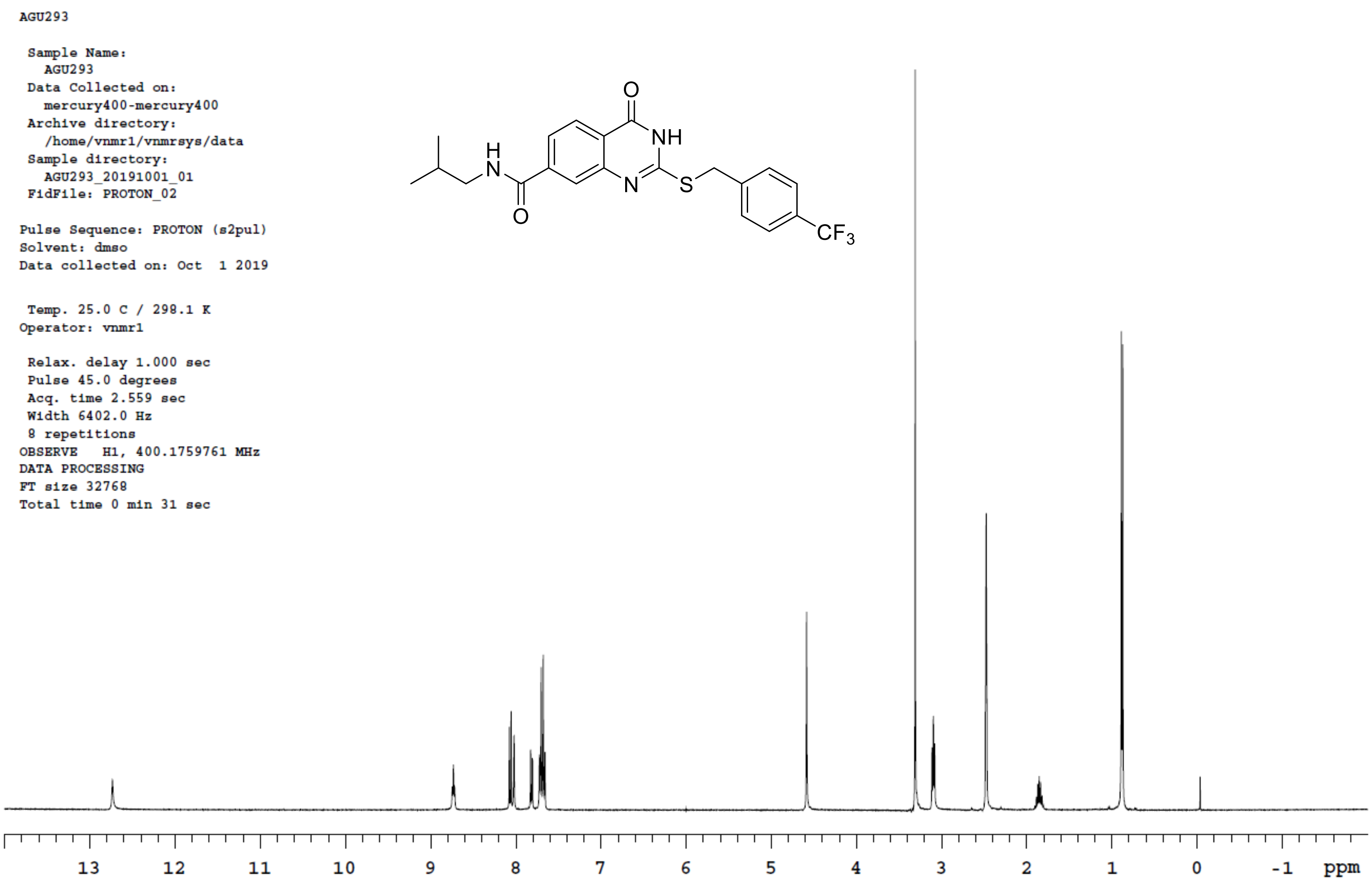

AGU293

Sample Name:
AGU293
Data Collected on:
mercury400-mercury400
Archive directory:
/home/vnmr1/vnmrsys/data
Sample directory:
AGU293_20191001_01
FidFile: CARBON_01

Pulse Sequence: CARBON (s2pul)
Solvent: dmso
Data collected on: Oct 1 2019

Temp. 25.0 C / 298.1 K
Operator: vnmr1

Relax. delay 1.000 sec
Pulse 45.0 degrees
Acq. time 1.550 sec
Width 21141.6 Hz
5000 repetitions
OBSERVE C13, 100.6243768 MHz
DECOUPLE H1, 400.1779555 MHz
Power 38 dB
continuously on
WALTZ-16 modulated
DATA PROCESSING
Line broadening 0.5 Hz
FT size 65536
Total time 3 hr, 40 min

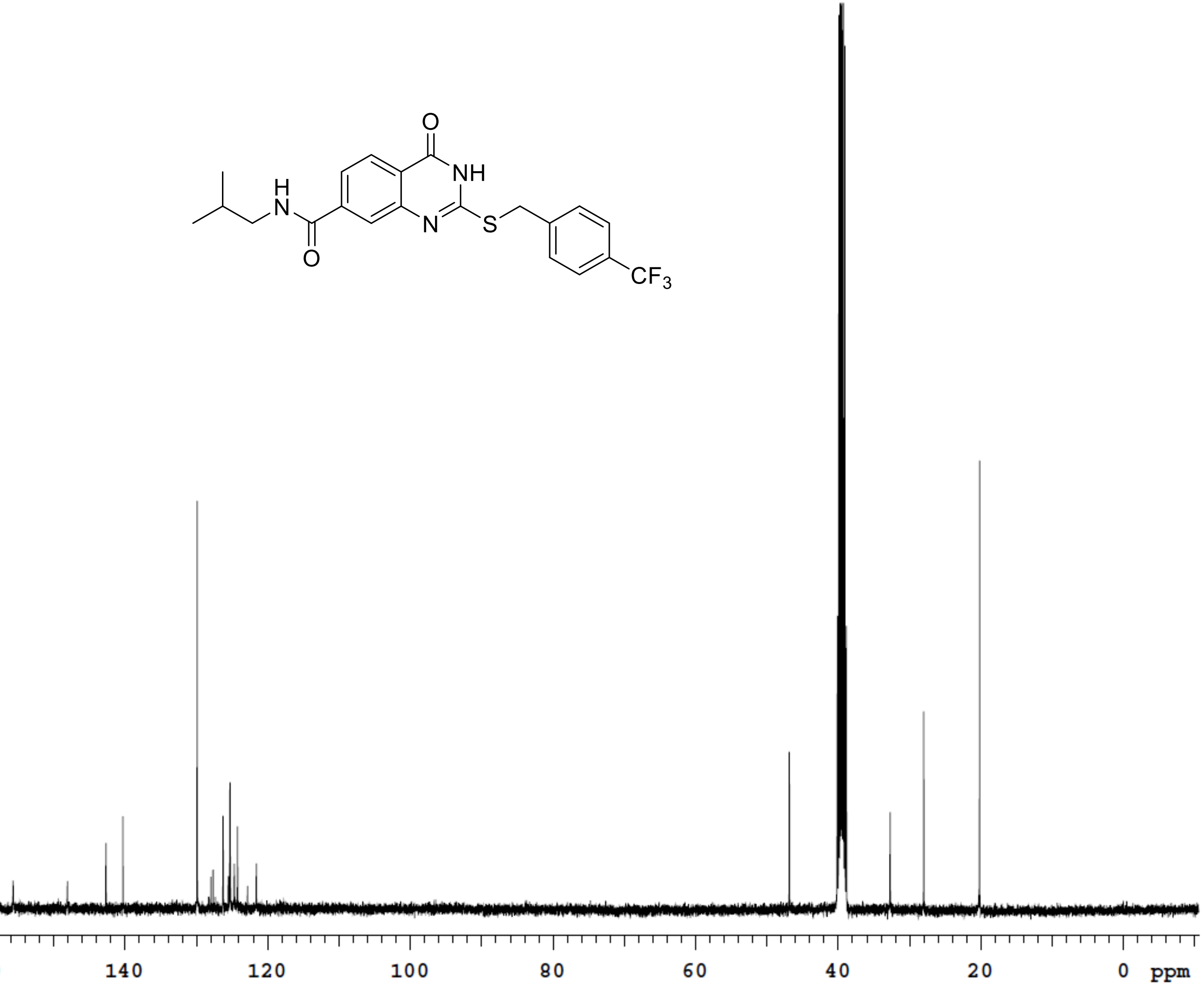

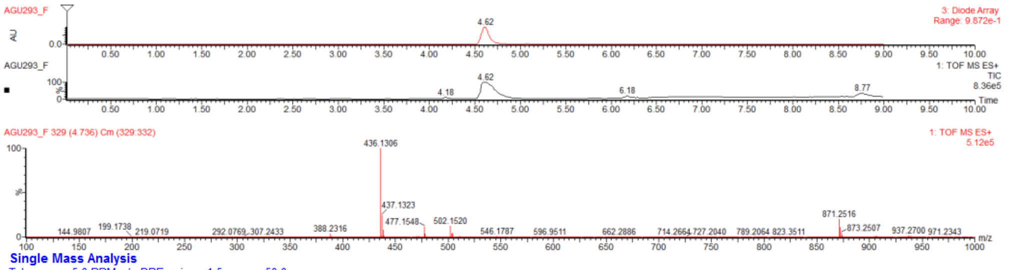

Single Mass Analysis
Tolerance = 5.0 PPM / DBE: min = -1.5, max = 50.0
Element prediction: Off
Number of isotope peaks used for i-FIT = 3

Monoisotopic Mass, Even Electron Ions
3 formula(e) evaluated with 1 results within limits (up to 50 closest results for each mass)
Elements Used:

Mass	Calc. Mass	mDa	PPM	DBE	Formula	i-FIT	i-FIT (Norm)	C	H	N	O	S	F
436.1306	436.1307	-0.1	-0.2	11.5	C21 H21 N3 O2 S F3	519.5	0.0	21	21	3	2	1	3

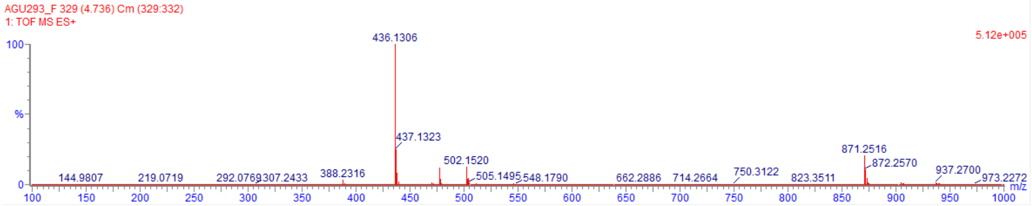

Figure S26. ^{1}H-NMR, ^{13}C-NMR and HRMS spectrums of 48

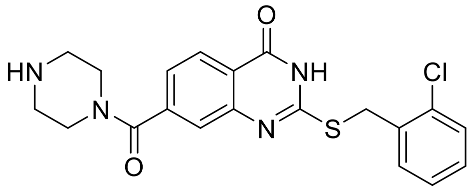

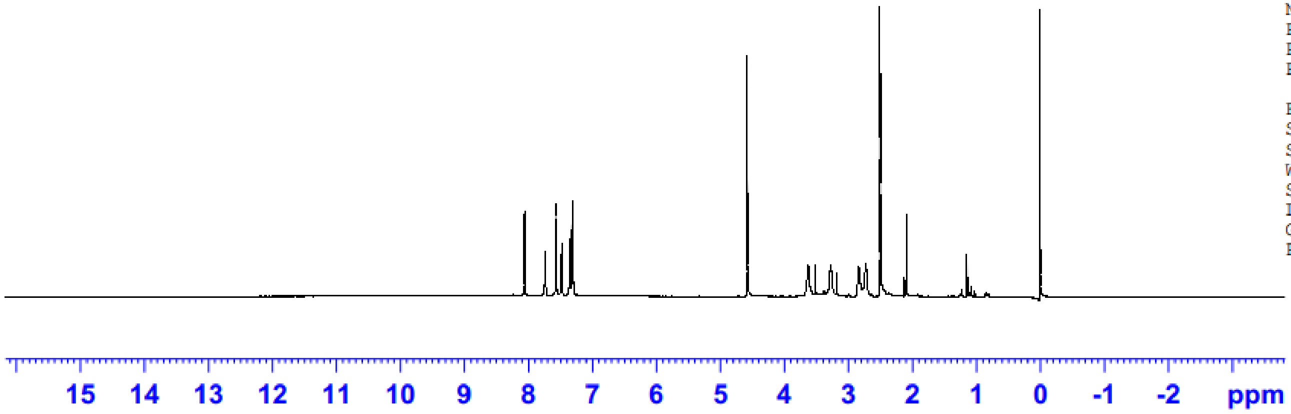

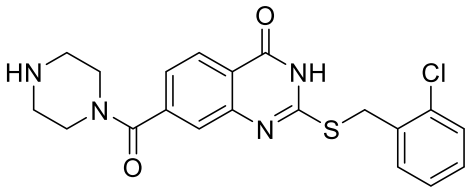

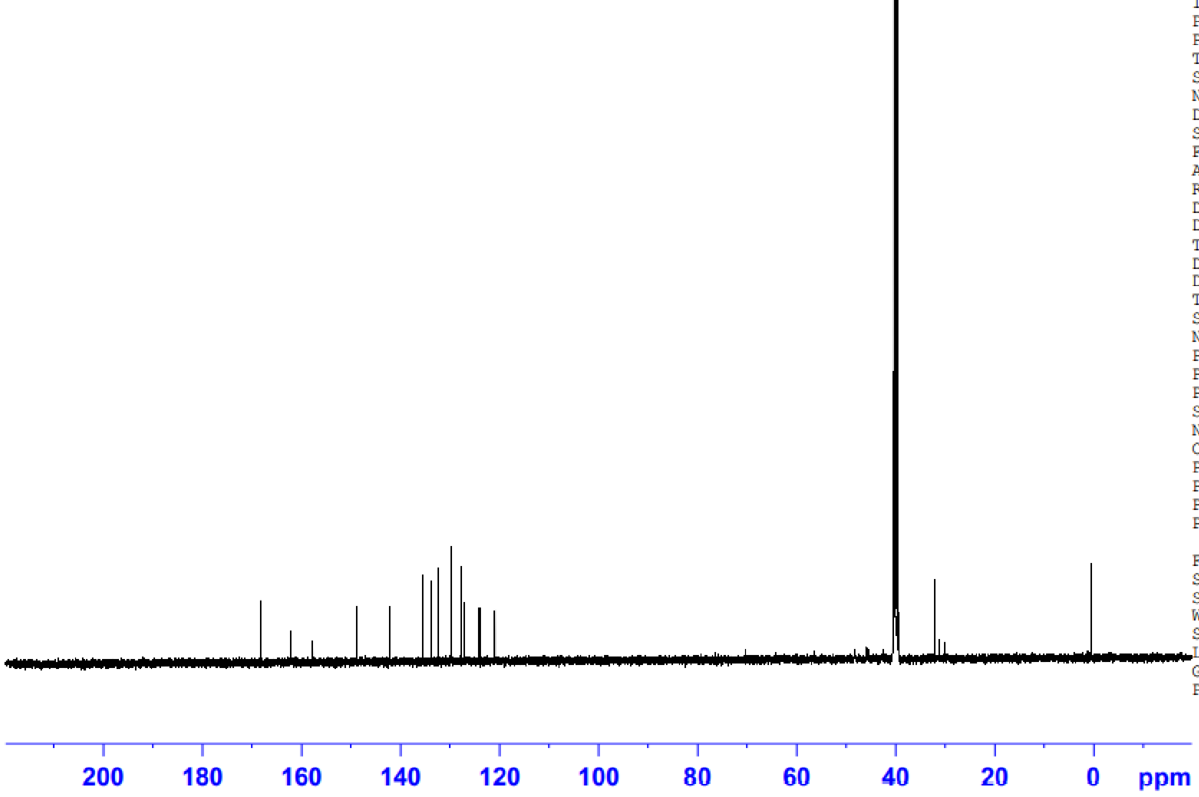


```
Current Data Parameters
NAME              SMY411
EXPNO                  1
PROCNO                 1

F2 - Acquisition Parameters
Date_           20210114
Time               15.01 h
INSTRUM           Avance
PROBHD   Z151574_0038 (
PULPROG          zgpg30
TD                 65536
SOLVENT             DMSO
NS                  3000
DS                     4
SWH            30120.482 Hz
FIDRES          0.919204 Hz
AQ             1.0878977 sec
RG                   101
DW                16.600 usec
DE                  6.50 usec
TE                 298.1 K
D1            2.00000000 sec
D11           0.03000000 sec
TD0                    1
SFO1         125.7703643 MHz
NUC1                 13C
P0                  3.33 usec
P1                 10.00 usec
PLW1         85.18099976 W
SFO2         500.1320005 MHz
NUC2                  1H
CPDPRG[2         waltz65
PCPD2              80.00 usec
PLW2         24.04299927 W
PLW12         0.24043000 W
PLW13         0.12093000 W

F2 - Processing parameters
SI                 32768
SF           125.7577885 MHz
WDW                   no
SSB      0
LB       0 Hz
GB       0
PC                  1.40
```

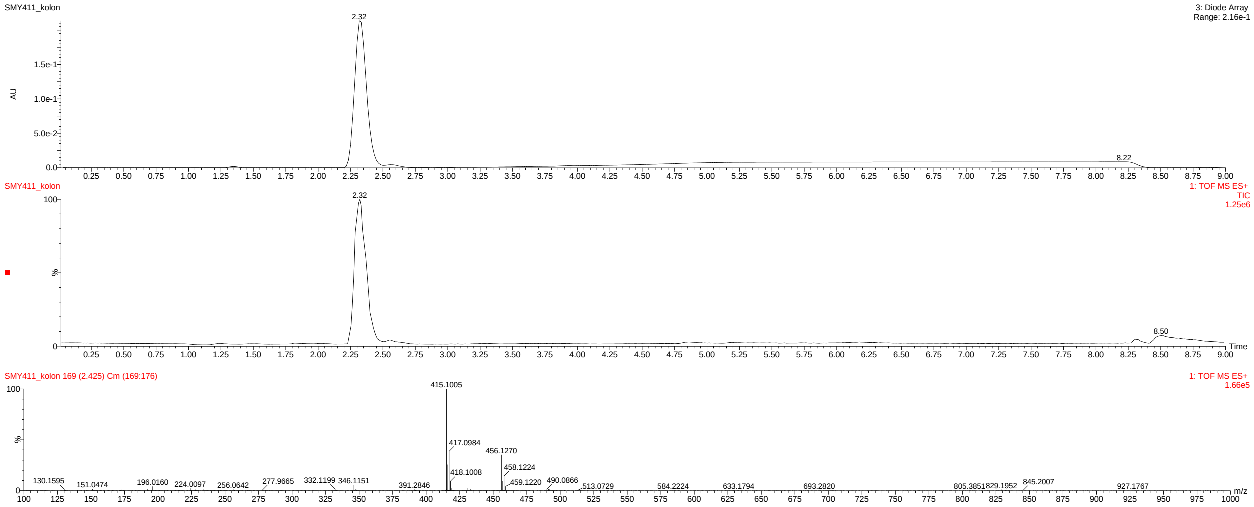

Single Mass Analysis
Tolerance = 5.0 PPM / DBE: min = -1.5, max = 50.0
Element prediction: Off
Number of isotope peaks used for i-FIT = 3
Monoisotopic Mass, Even Electron Ions
71 formula(e) evaluated with 1 results within limits (up to 50 closest results for each mass)
Elements Used:

Mass	Calc. Mass	mDa	PPM	DBE	Formula	i-FIT	i-FIT (Norm)	C	H	N	O	S	Cl
415.1005	415.0996	0.9	2.2	12.5	C20 H20 N4 O2 S Cl	367.2	0.0	20	20	4	2	1	1

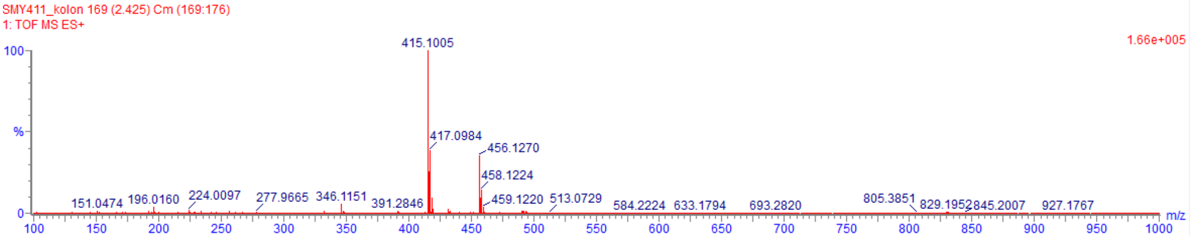

Figure S27. ^{1}H-NMR, ^{13}C-NMR and HRMS spectrums of 49

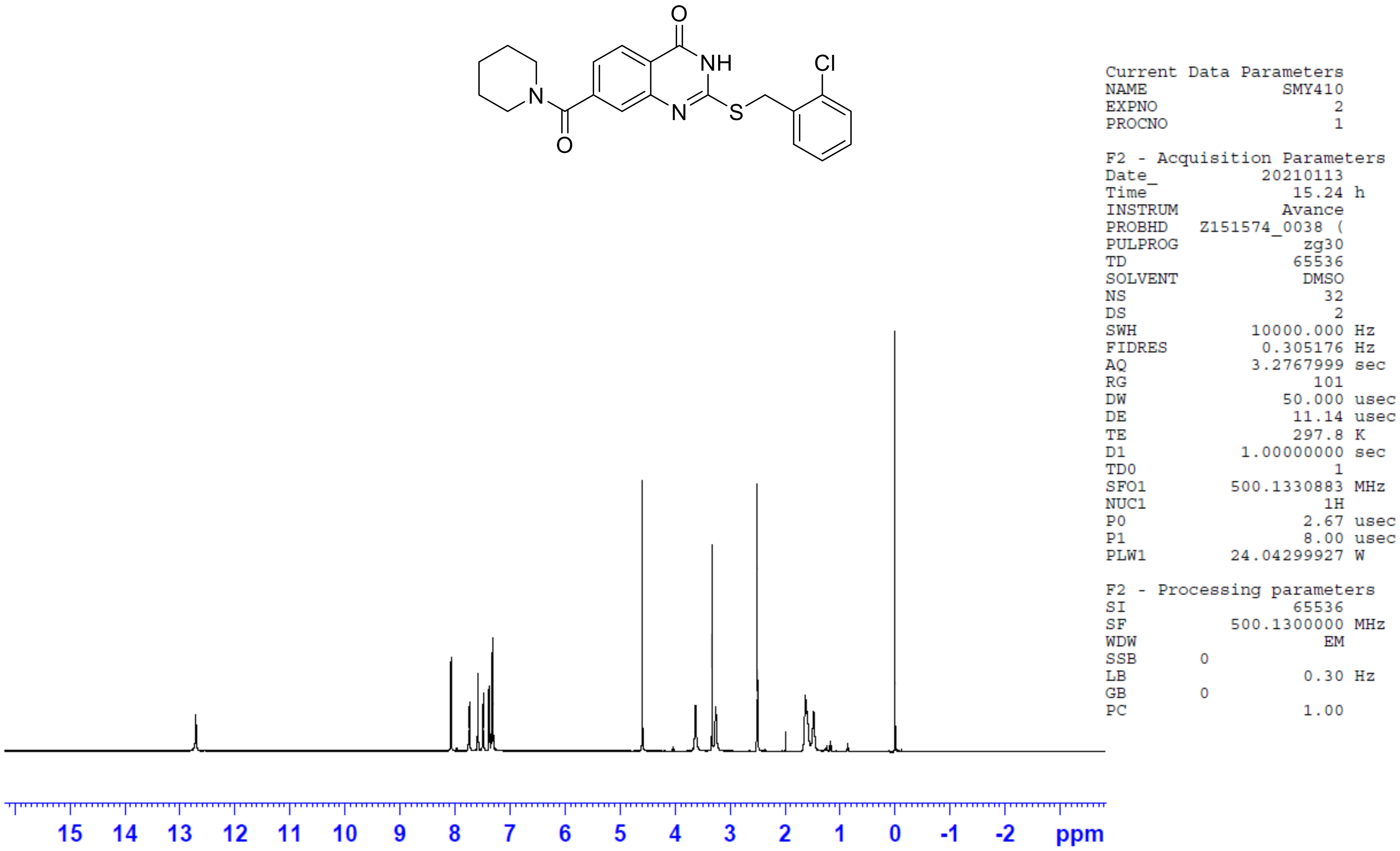

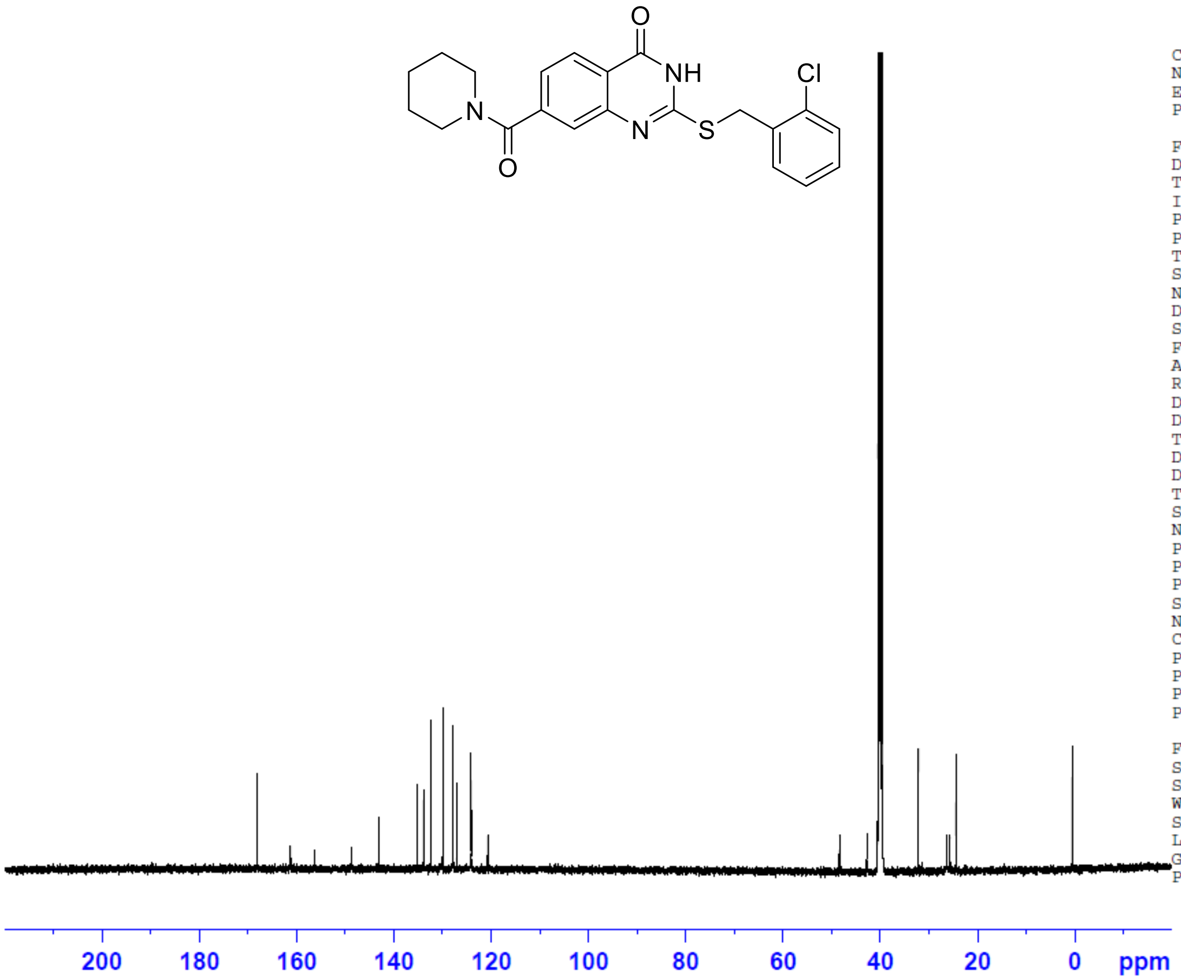


```
Current Data Parameters
NAME              SMY410
EXPNO                  1
PROCNO                 1

F2 - Acquisition Parameters
Date_           20210113
Time               14.31 h
INSTRUM           Avance
PROBHD   Z151574_0038 (
PULPROG          zgpg30
TD                 65536
SOLVENT             DMSO
NS                  2000
DS                     4
SWH            30120.482 Hz
FIDRES          0.919204 Hz
AQ             1.0878977 sec
RG                   101
DW                16.600 usec
DE                  6.50 usec
TE                 298.9 K
D1            2.00000000 sec
D11           0.03000000 sec
TD0                    1
SFO1         125.7703643 MHz
NUC1                 13C
P0                  3.33 usec
P1                 10.00 usec
PLW1         85.18099976 W
SFO2         500.1320005 MHz
NUC2                  1H
CPDPRG[2         waltz65
PCPD2              80.00 usec
PLW2         24.04299927 W
PLW12         0.24043000 W
PLW13         0.12093000 W

F2 - Processing parameters
SI                 32768
SF           125.7577885 MHz
WDW                   EM
SSB      0
LB                  1.00 Hz
GB       0
PC                  1.40
```

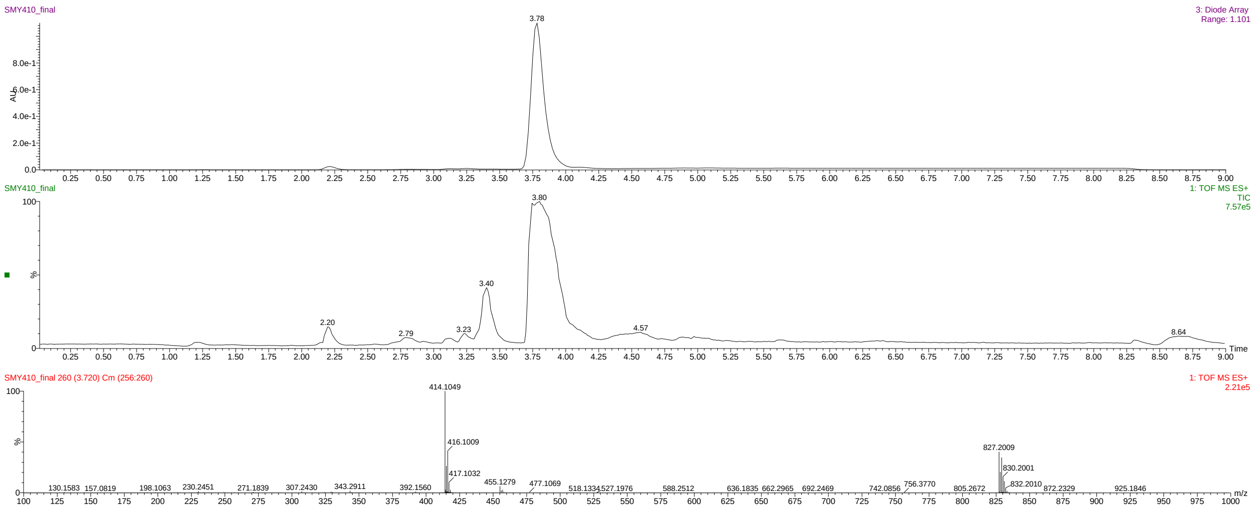

Single Mass Analysis
Tolerance = 5.0 PPM / DBE: min = -1.5, max = 50.0
Element prediction: Off
Number of isotope peaks used for i-FIT = 3
Monoisotopic Mass, Even Electron Ions
47 formula(e) evaluated with 1 results within limits (up to 50 closest results for each mass)
Elements Used:

Mass	Calc. Mass	mDa	PPM	DBE	Formula	i-FIT	i-FIT (Norm)	C	H	N	O	S	Cl
414.1049	414.1043	0.6	1.4	12.5	C21 H21 N3 O2 S Cl	405.6	0.0	21	21	3	2	1	1

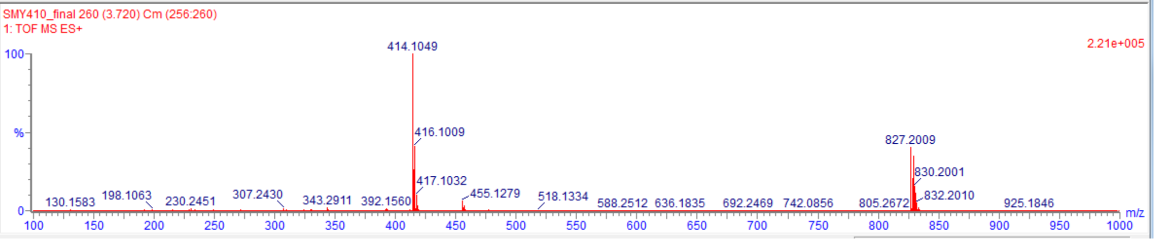

Figure S28. ^{1}H-NMR, ^{13}C-NMR and HRMS spectrums of 50

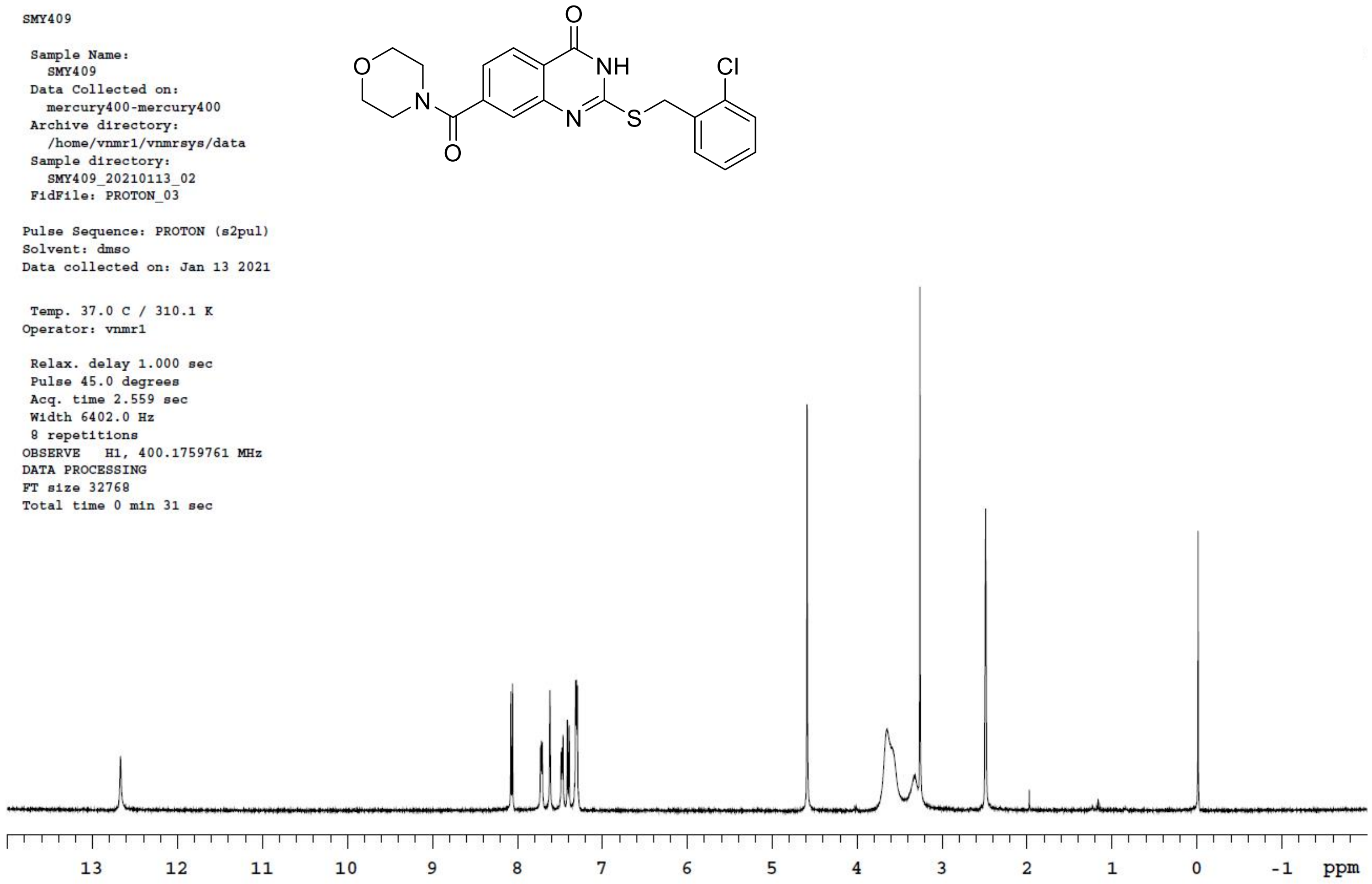

SMY409

Sample Name:
SMY409
Data Collected on:
mercury400-mercury400
Archive directory:
/home/vnmr1/vnmrsys/data
Sample directory:
SMY409_20210113_02
FidFile: CARBON_01

Pulse Sequence: CARBON (s2pul)
Solvent: dmso
Data collected on: Jan 13 2021

Temp. 37.0 C / 310.1 K
Operator: vnmr1

Relax. delay 1.000 sec
Pulse 45.0 degrees
Acq. time 1.550 sec
Width 21141.6 Hz
4000 repetitions
OBSERVE C13, 100.6243842 MHz
DECOUPLE H1, 400.1779555 MHz
Power 38 dB
continuously on
WALTZ-16 modulated
DATA PROCESSING
Line broadening 0.5 Hz
FT size 65536
Total time 2 hr, 56 min

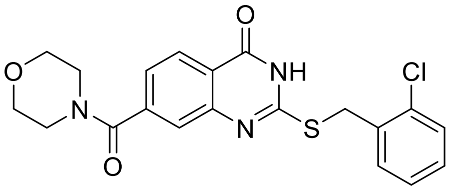

Agilent Technologies

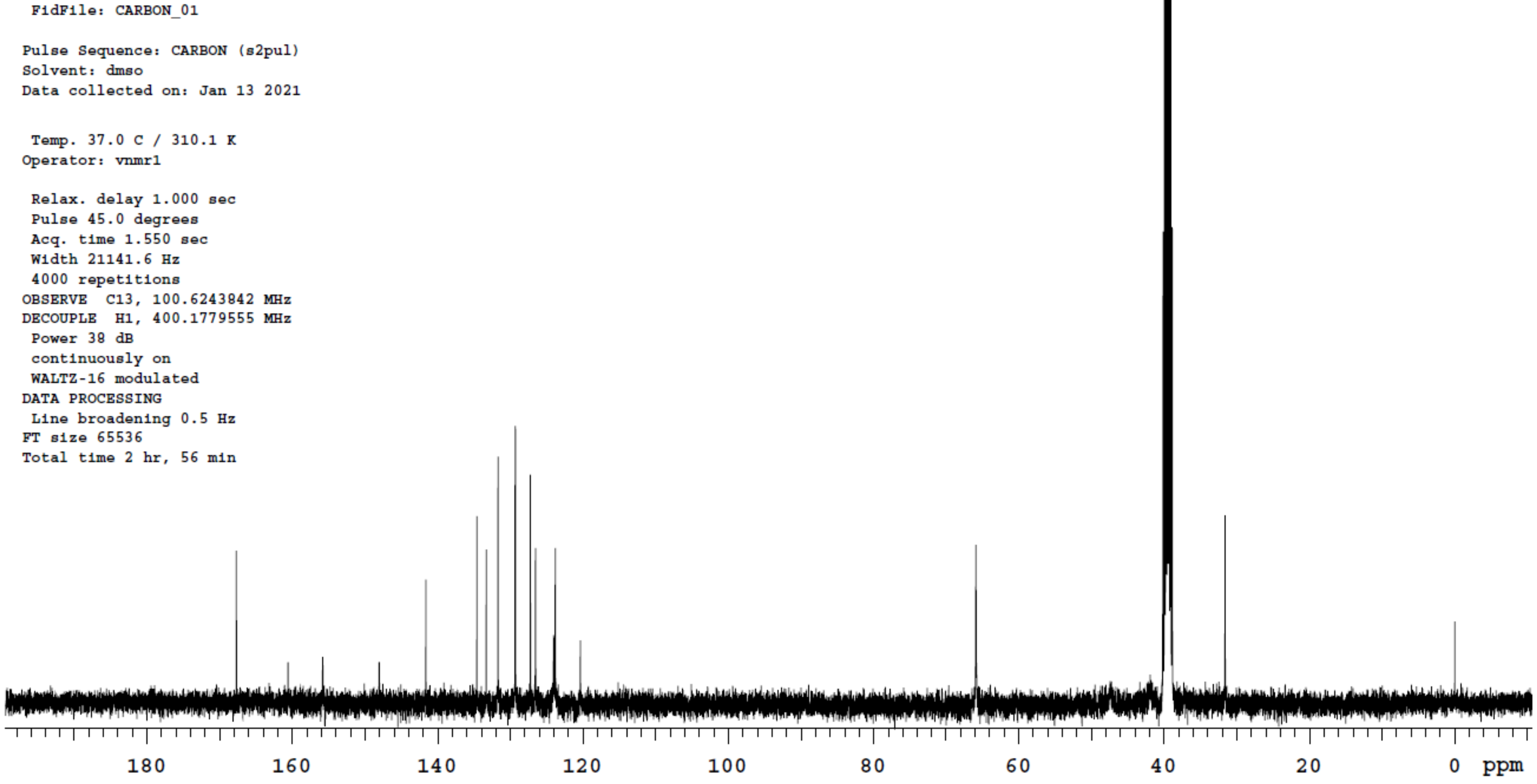

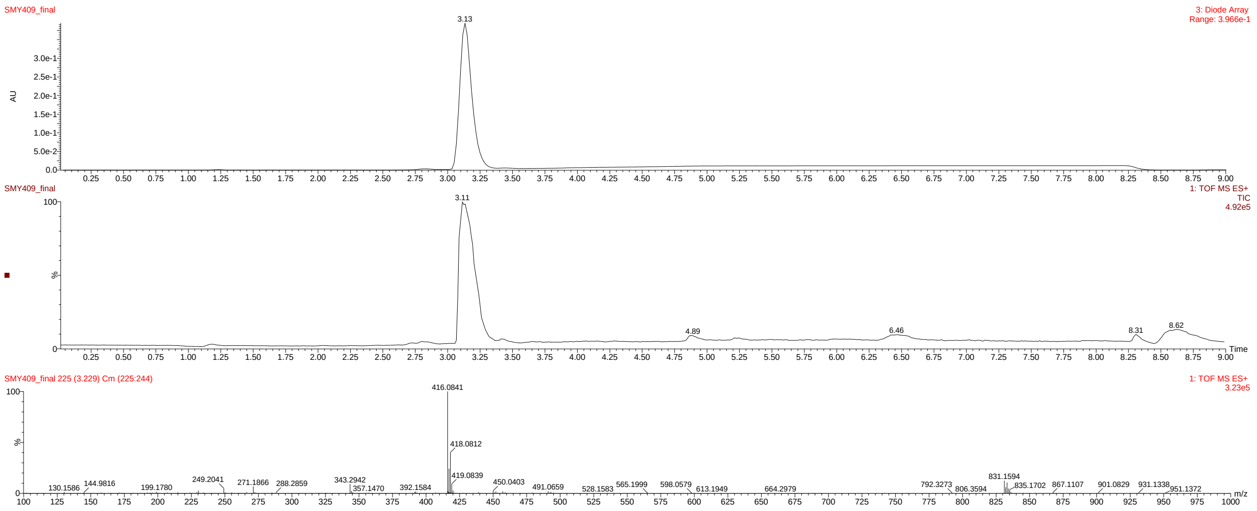

Single Mass Analysis
Tolerance = 5.0 PPM / DBE: min = -1.5, max = 50.0
Element prediction: Off
Number of isotope peaks used for i-FIT = 3
Monoisotopic Mass, Even Electron Ions
21 formula(e) evaluated with 1 results within limits (up to 50 closest results for each mass)
Elements Used:

Mass	Calc. Mass	mDa	PPM	DBE	Formula	i-FIT	i-FIT (Norm)	C	H	N	O	S	Cl
416.0841	416.0836	0.5	1.2	12.5	C20 H19 N3 O3 S Cl	536.6	0.0	20	19	3	3	1	1

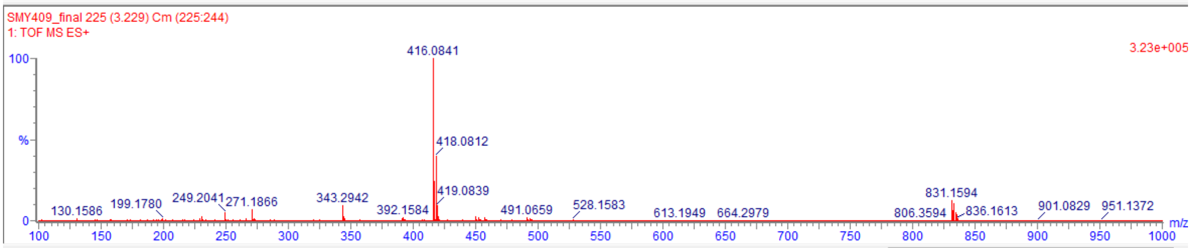

Figure S29. ^{1}H-NMR, ^{13}C-NMR and HRMS spectrums of 55

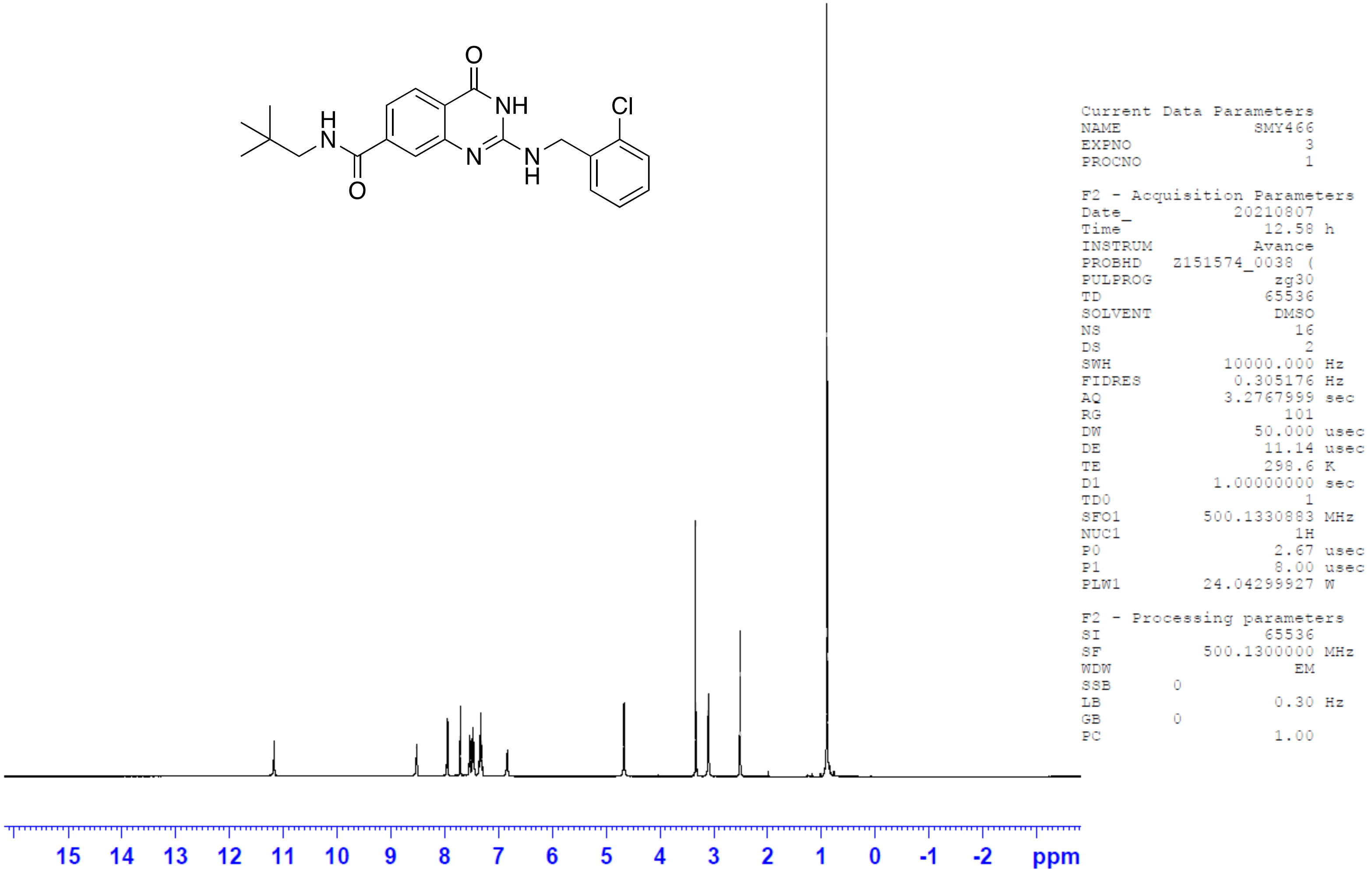

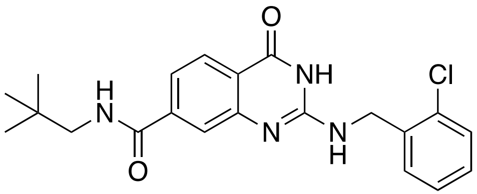

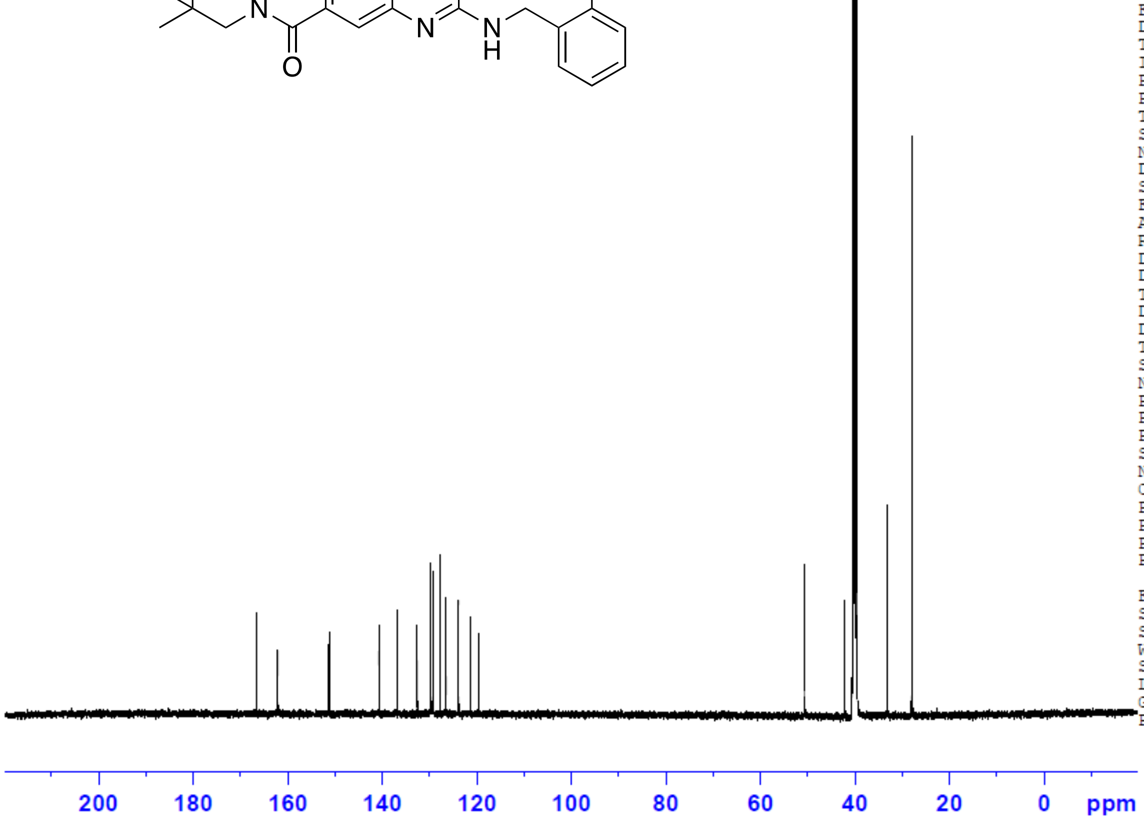


```
Current Data Parameters
NAME              SMY466
EXPNO                  2
PROCNO                 1

F2 - Acquisition Parameters
Date_           20210807
Time_              12.14 h
INSTRUM           Avance
PROBHD   Z151574_0038 (
PULPROG          zgpg30
TD                 65536
SOLVENT             DMSO
NS                  2000
DS                     4
SWH            30120.482 Hz
FIDRES          0.919204 Hz
AQ             1.0878977 sec
RG                   101
DW                16.600 usec
DE                  6.50 usec
TE                 299.7 K
D1            2.00000000 sec
D11           0.03000000 sec
TD0                    1
SFO1         125.7703643 MHz
NUC1                 13C
P0                  3.33 usec
P1                 10.00 usec
PLW1         85.18099976 W
SFO2         500.1320005 MHz
NUC2                  1H
CPDPRG[2         waltz65
PCPD2              80.00 usec
PLW2         24.04299927 W
PLW12         0.24043000 W
PLW13         0.12093000 W

F2 - Processing parameters
SI                 32768
SF           125.7577885 MHz
WDW                   EM
SSB      0
LB                  1.00 Hz
GB       0
PC                  1.40
```

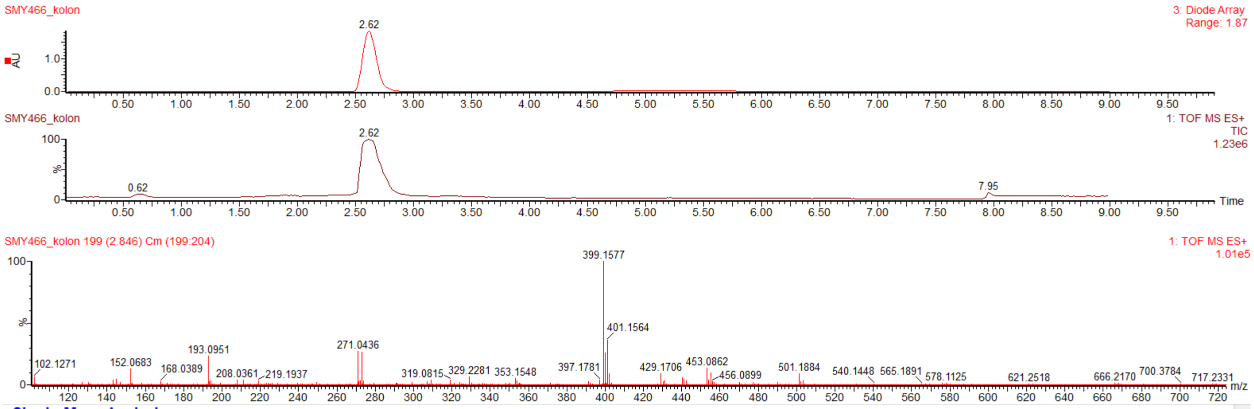

Single Mass Analysis

Tolerance = 5.0 PPM / DBE: min = -1.5, max = 50.0

Element prediction: Off

Number of isotope peaks used for i-FIT = 3

Monoisotopic Mass, Even Electron Ions

16 formula(e) evaluated with 1 results within limits (up to 50 closest results for each mass)

Elements Used:

Mass	Calc. Mass	mDa	PPM	DBE	Formula	i-FIT	i-FIT (Norm)	C	H	N	O	Cl
399.1577	399.1588	-1.1	-2.8	11.5	C21 H24 N4 O2 Cl	406.6	0.0	21	24	4	2	1

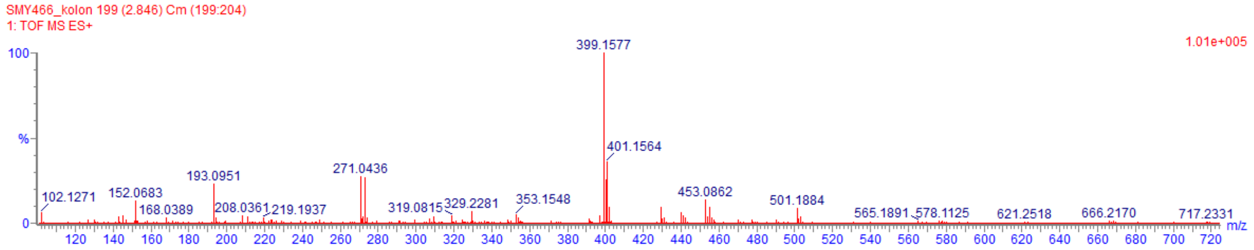

References

(1) Schrödinger Release 2022-2: QikProp, Schrödinger, LLC, New York, NY, **2022.**

(2) Bairoch, A.; Apweiler, R. The SWISS-PROT protein sequence database and its supplement TrEMBL in 2000. *Nucleic Acids Res* **2000**, *28* (1), 45-48. DOI: 10.1093/nar/28.1.45 From NLM Medline.

(3) Notredame, C.; Higgins, D. G.; Heringa, J. T-Coffee: A novel method for fast and accurate multiple sequence alignment. *J Mol Biol* **2000**, *302* (1), 205-217. DOI: 10.1006/jmbi.2000.4042.

(4) Waterhouse, A. M.; Procter, J. B.; Martin, D. M.; Clamp, M.; Barton, G. J. Jalview Version 2--a multiple sequence alignment editor and analysis workbench. *Bioinformatics* **2009**, *25* (9), 1189-1191. DOI: 10.1093/bioinformatics/btp033.
